# Small RNA Profile in Moso Bamboo Root and Leaf Obtained by High Definition Adapters

**DOI:** 10.1371/journal.pone.0103590

**Published:** 2014-07-31

**Authors:** Ping Xu, Irina Mohorianu, Li Yang, Hansheng Zhao, Zhimin Gao, Tamas Dalmay

**Affiliations:** 1 School of Biological Sciences, University of East Anglia, Norwich, United Kingdom; 2 State Forestry Administration Key Open Laboratory on the Science and Technology of Bamboo and Rattan, International Centre for Bamboo, Rattan, Beijing, China; The Ohio State University, United States of America

## Abstract

Moso bamboo (*Phyllostachy heterocycla cv. pubescens L.*) is an economically important fast-growing tree. In order to gain better understanding of gene expression regulation in this important species we used next generation sequencing to profile small RNAs in leaf and roots of young seedlings. Since standard kits to produce cDNA of small RNAs are biased for certain small RNAs, we used High Definition adapters that reduce ligation bias. We identified and experimentally validated five new microRNAs and a few other small non-coding RNAs that were not microRNAs. The biological implication of microRNA expression levels and targets of microRNAs are discussed.

## Introduction

Small non-coding RNAs (sRNAs) with the size of 20–24 nucleotides (nts) that are generated by one of the Dicer-like proteins function in transcriptional or post-transcriptional regulation of gene expression [1]. Plant sRNAs show high diversity and one of the best characterized group of sRNAs is the microRNAs (miRNAs), which are mainly 21 nts although some are 20, 22 and 24 mers. Some miRNAs are conserved across all plant species but many newly identified miRNAs are species or family specific. In addition to miRNAs, there are several groups of small interfering RNAs (siRNAs) involved in the regulation of gene expression such as heterochromatic small interfering RNAs (hc-siRNAs) that are usually 24 mers [2], trans-acting small interfering RNAs (ta-siRNAs) that are 21 mers [3] and anti-sense origin siRNAs that are produced from overlapping anti sense transcripts [4]. sRNAs are involved in diverse biological processes including developments and responses to environmental changes [5] thus it is important to characterize sRNAs in non-model but economically important plants.

Bamboo is one of the most important forest resources and belongs to the grass family. There are more than 1500 species of bamboo within 87 genera [6]. Bamboo has relatively long period of vegetative growth varying from a few years to decades, which makes it hard for traditional genetic improvement. The advance in next generation sequencing (NGS) technology opens new opportunities for studying the molecular biology of bamboo. The genomic sequence of Moso bamboo (*Phyllostachy heterocycla cv. pubescens L.*) has been reported recently and is becoming a valuable resource [7]. Moso bamboo is a large fast-growing woody bamboo and a major bamboo species for timber and food production in Asia. mRNA and miRNA analysis has been carried out using developing culms during fast growing stage [8], where RNA-seq libraries were constructed using the Illumina method. Many conserved miRNAs were found and putative new miRNAs were predicted. However, their expression levels were not confirmed experimentally. In addition, some miRNAs were found in the leaf tissues of ma bamboo (*Dendrocalamus latiflorus L.*) [9], but the genomic sequence is not available for this species therefore the analysis could not be completed.

High throughput sRNA studies rely on NGS results; however, different NGS platforms often produce different sRNA profiles [10]. The difference has been attributed to ligation bias due to the preference of RNA ligases to join molecules (in this case sRNAs and adapters) that can anneal to each other and form a structure favoured by the ligase [11]–[13]. Therefore libraries generated with different adapters produce different sRNA profiles. Since different NGS platforms or even different versions of the library generation kit for the same platform (such as Illumina v. 1.0, v. 1.5 and TruSeq) contain different adapters, these platforms and kits produce different sRNA profiles because they all rely on adapters with fixed sequences that determine the ligation bias. Adapters with degenerated nucleotides at the ligating ends (called High Definition (HD) adapters) can reduce the ligation bias because sRNAs can anneal to a pool of different sequences instead of a single adapter sequence [12], [13]. sRNA libraries generated with HD adapters were found to recover more different sRNA sequences and the abundance of a sRNA sequence read correlated more quantitatively with the real expression level [13]. Here, the HD adapters were used for cloning the sRNAs in the leaves and roots of moso bamboo seedlings. The sRNA profiles were analysed and the expression of the new miRNAs with expressed miRNA* sequences were confirmed by Northern blot analysis.

## Materials and Methods

### Plant materials

The seeds of Moso bamboo were soaked in 0.4% KMnSO_4_ solution for 6 hrs, rinsed with distilled water five times, and further soaked in distilled water overnight. The soaked seeds were planted in moist vermiculite in a closed container and kept at 25°C under light with intensity of 100–200 µmol·m^−2^·s^−1^ for two weeks followed by removing the cover film of the container so that the seeds were kept in the same container for about 2 months under the same growing conditions. The seedlings were transferred to pots with turfy soil and grown under the same conditions. The leaf, stem and root tissues were harvested and stored in liquid nitrogen when the seedlings were at 5–6 leaf stage.

### RNA extraction and small RNA library construction

Total RNA was extracted by using TRI Reagent Solution (Ambion) following the manufacturer’s protocol. Small RNA fractions of the leaf and root total RNA samples were further isolated by using *mir*VanamiRNA Isolation Kit (Ambion) following the protocol provided by the manufacturer. 2 µg of sRNA from each sample was ligated to 3′ and 5′ HD adapters [13] by using the ScriptMiner Small RNA-seq Library preparation Kit (Epicenter) following its protocol (but replacing the adapters provided in the kit with HD adapters with identical sequences but containing the four degenerated nucleotides HD tag). The ligated products were then reverse transcribed and PCR amplified. The PCR products expected to contain 20–24 bp cDNA inserts were gel-purified and sequenced on the Illumina HiSeq2500 sequencer.

### Northern blot analysis

Northern blot analysis was used to confirm the expression levels of known miRNAs and verify the new miRNAs and some other new sRNAs following the previously published protocol (Lopez-Gomollon et al. 2012). Briefly, 5 µg of total RNA from stem, root and leaf tissues were denatured and loaded into denaturing 16% polyacrylamide gel. The RNA was transferred to Hybond NX (Amersham) nylon membrane through semi-dry electrophoresis transfer system (Bio-Rad), and chemical cross-linking was done at 60°C for 90 minutes by using 1-ethyl-3-(dimethylaminopropyl) carbodiimide (Sigma). The probes were generated by labelling the oligonucleotides that were reverse complementary to the sRNAs of interest with γP^32^-ATP. The membranes were hybridized with the probes at 37°C overnight. The list of the probe sequences used for northern blot is provided in supplementary Table 1.

**Table 1:** General Information of the libraries.

		leaf					root				
		total reads	proportion	unique reads	proportion	complexity	total reads	proportion	unique reads	proportion	complexity
total		9225007		1835808		0.199	12790713		2929630		0.23
chloroplast genome		2334231	0.2530	161696	0.0880	0.0692	44400	0.0035	8454	0.0029	0.1900
mitochondria genome		899938	0.0975	68652	0.0374	0.0763	144661	0.0113	25302	0.0086	0.1749
genome match*		7645564	0.8287	1242905	0.677	0.1626	9507995	0.7434	1650282	0.5633	0.1736
	coding region of mRNAs	398533	0.0521	129660	0.1043	0.3253	330006	0.0347	157789	0.0956	0.4781
	rRNAs	2883079	0.3771	78906	0.0635	0.0274	4713899	0.4958	73908	0.0448	0.0157
	tRNA	527747	0.069	12673	0.0102	0.024	1388039	0.146	9738	0.0059	0.007
	snRNA	12709	0.0017	3654	0.0029	0.2875	36269	0.0038	4269	0.0026	0.1177
	Transposable elements	608844	0.0796	372443	0.2996	0.6117	808457	0.085	556477	0.3372	0.6883
	known microRNAs	241378	0.0316	937	0.0008	0.0039	182688	0.0192	1065	0.0006	0.0058
	new microRNAs	11138	0.0015	341	0.0003	0.0306	16381	0.0017	301	0.0002	0.0183

*the current genome sequence does not separate chloroplast and mitochondrion genome sequences from the nuclear genome.

### Bioinformatics analysis

We used version 1.0 of the bamboo genome [7] and the annotations available on the ICBR-CAFNET website (http://202.127.18.221/bamboo/down.php). We downloaded the bamboo chloroplast genome, accession NC_015817 from (http://chloroplast.ocean.washington.edu) and used the publicly available annotations [14]. First, the fastq files from NGS were converted to fasta files and sequence reads with no Ns were kept for further analysis. Next, the first 8 nt of the 3′ adapters were identified and removed and then four nucleotides on the 5′ and 3′ ends of the reads were trimmed (which corresponded to the NNNN tags on the HD adapters; see: Sorefan et al. 2012) using the UEA sRNA Workbench [15]. The sequence reads were mapped to the bamboo genome and the bamboo chloroplast genome with 0 mis-matches, in non-redundant format, using PatMaN [16]. The abundance of sequenced reads was normalized to the total number of reads in the sample, as indicated in [17]. The differentially expressed reads were identified using offset fold change as described in [18], using an offset of 20. The miRNAs were identified using the miRCat and miRProf tools within the sRNA Workbench [15] and custom made Perl scripts (Strawberry Perl v 5.18.2.1). The miRNA targets were predicted using the target prediction tool within the UEA sRNA Workbench [15]. The rules used for target prediction are based on those suggested by Allen et al. and Schwab et al. [19], [20]. Specifically, no more than four mismatches between miRNA and target (G-U bases count as 0.5 mismatches) are allowed, no more than two adjacent mismatches in the miRNA/target duplex are permitted, no adjacent mismatches in positions 2–12 of the miRNA/target duplex (5′ of miRNA) and no more than 2.5 mismatches in positions 1–12 are accepted, no mismatches in positions 10–11 of miRNA/target duplex are permitted. In addition, the minimum free energy (MFE) of the miRNA/target duplex should be > = 74% of the MFE of the miRNA bound to its perfect complement.

## Results

### sRNA libraries from bamboo leaf and root

About 10 million (M) and 18 M raw reads were obtained from deep sequencing of bamboo leaf and root sRNA libraries, respectively. About 9 M (leaf) and 12 M (root) reads contained the 3′ adapter preceded with potential sRNAs that were 17–33 nts (Table 1). The root library contained ∼2.9 M unique sequences while the leaf library was made up of ∼1.8 M unique sequences, thus the overall sequence complexities (defined as the ratio of unique reads to all reads) for the two libraries were 16–17%. About 83% and 74% of the total reads in leaf and root library were mapped to the published moso bamboo genome (Peng, 2013 #60). Among the genome matching sRNAs, 5.2% of the reads in leaf library and 3.5% of the reads in root library were mapped to coding region. Distribution of read mapping to other genomic regions is shown in Table 1.

**Figure 1:**
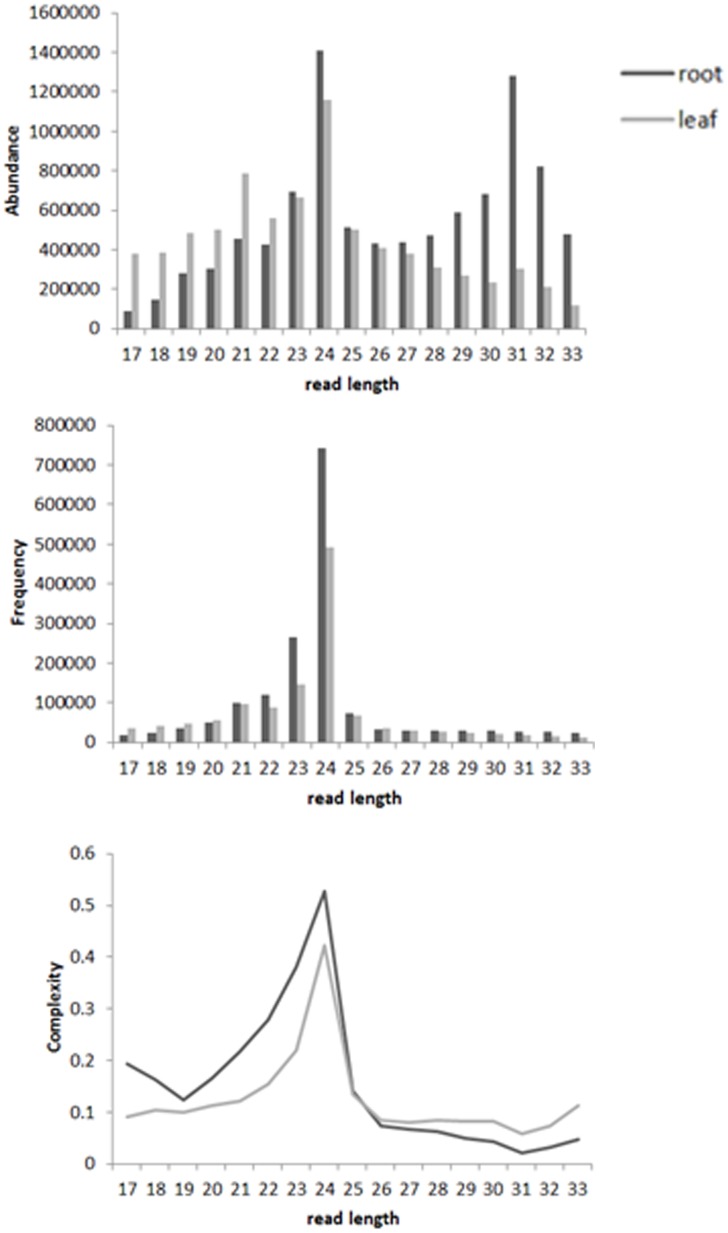
Overall summary of genome matching reads. (**a**) Size class distribution for total reads (redundant reads); (**b**) size class distribution for unique reads (non-redundant reads); (**c**) Complexity distribution. Complexity is defined as the ratio between unique reads and redundant reads.

Similar to other plant species, size class distribution was bimodal where the most abundant sequences were 24 mers followed by the 21 mers in leaf library. The root library showed a slightly different size class distribution because the most abundant 24 mers were followed by 31 mers and even the 23 mer sRNAs were slightly more abundant than the 21 mers (Figure 1a). The 24 mer reads showed the highest complexity while the 21 and 31 mer sequences had very low complexity (Figure 1b and 1c). The majority of transposon mapping sRNAs were 24 mers and 40% of unique 24 mer reads mapped to transposons (Supplementary Figure 1).

### Chloroplast mapping reads

The sRNAs in leaf and root libraries mapped to the chloroplast genome exhibit very strong sequence, sequence abundance and distribution pattern differences. We found that 25% of the total reads in leaf library were mapped to the chloroplast genome while this percentage was only 0.3% in root libraries (Table 1). Nearly 50% of the 100 most abundant sRNAs were mapped to the chloroplast genome in leaf library but none in root library. Approximately 70% of the chloroplast genome matching reads were mapped to rRNA and tRNA genes, ∼23% of the reads mapped to intergenic regions and 5.6% to protein coding regions. The remaining reads were mapped to the junction sequences crossing coding and non-coding sequences and repeat sequences (Table 2).

**Table 2:** General information of the sRNAs mapped to bamboo plastid genome.

	leaf					root				
	total reads	proportion	unique reads	proportion	complexity	total reads	proportion	unique reads	proportion	complexity
total	2334231		161696		0.0692	44400		8454		0.1904
coding region	130511	0.0559	54194	0.3351	0.4152	2617	0.0589	1529	0.1809	0.5843
rRNA and tRNA	1634486	0.7002	66485	0.4111	0.0406	35157	0.7918	4738	0.5604	0.1348
repeat sequence	1006	0.0004	358	0.0022	0.3558	37	0.0008	12	0.0014	0.3243
intergenic region	543609	0.2328	34898	0.2158	0.0642	6082	0.1370	1939	0.2294	0.3188
junction*	24619	0.0105	5761	0.0356	0.2340	507	0.0114	236	0.0279	0.4655

*the junctional sequence between coding and non-coding region.

**Figure 2:**
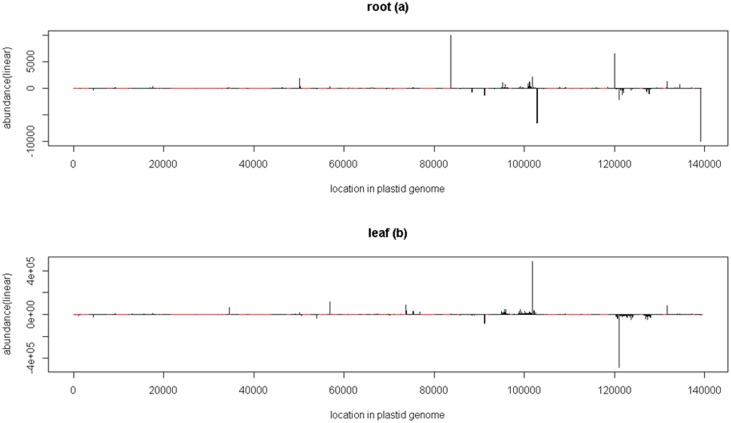
sRNA presence plot on the plastid genome for the root (a) and leaf (b) libraries. The abundance of the sRNAs is represented in linear scale. The reads mapping to the positive and negative strands are indicated by the sign of the abundance (positive and negative, respectively). The location of the chloroplast CDSs is indicated in red.

The size class distribution of chloroplast matching reads does not show enrichment for any particular size sRNAs (Supplementary Figure 1), but complexity is very low for the rRNA, tRNA and intergenic region mapping sRNAs (Table 2). The highly accumulated sRNAs were mapped to a few specific loci, which are shown in the sRNA presence plots for the chloroplast genome (Figure 2). Many sRNA loci were less than 100 bp away from the coding regions such as atpH, rbcL, psaJ, clpP, psbH, rpoA and rps3, and some were about 100–500 bp away from the coding regions of psbZ, ndhJ, atpE, rpl33, rpl22, rpl23 and rps7. Some of these sequences were the footprints of pentatricopeptide repeat (PPR) or PPR-like protein binding sites that were previously experimentally identified or predicted in barley and *Arabidopsis* [21].

**Figure 3:**
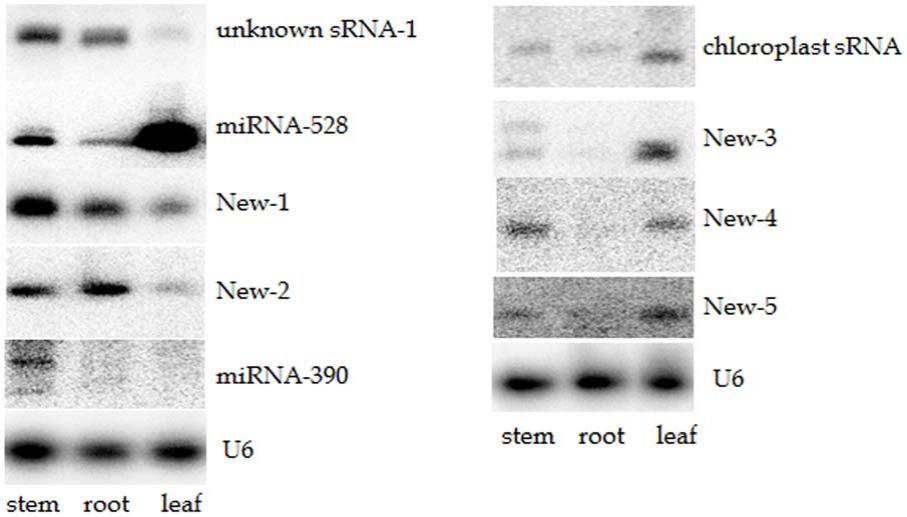
Detection of bamboo miRNAs by Northern blot analysis. The bottom panel shows the top half of the PAGE gel stained with ethidium bromide showing equal loading of total RNA.

The most abundant sequence among the chloroplast genome matching sRNAs is a 31 nt long sequence: TATCGAGTAGACCCTGTCGTTGTGAGAATTC. The first nucleotide of this sRNA maps to a position that is 59 nt upstream of the translational start site of rbcL. In barley, that position is the start transcriptional start site and the first ∼30 nts of the 5′UTR was the predicted binding site of the barley MRL1-PPR protein [21]. This 31 nt sRNA was further detected by northern blot analysis (Figure 3) only in leaf tissue and it is most likely a PPR protected RNA fragment.

### Known miRNAs

Based on sequence similarity to miRNAs in miRBase (http://www.mirbase.org/; release 20) [22], 22 miRNA families with normalized abundance above 100 were identified (Table 3). In total, 75 miRNA and 24 miRNA* variants of these families were found (Supplementary Table 2). Among them, three miRNAs miR528, miR444 and miR1432 are likely to be monocot specific [23], [24]. These miRNA variants were mapped to the bamboo genome and the sequences of their flanking regions were further investigated for potential hairpin structure. This analysis resulted in 141 precursor loci, out of which 13 had both mature and miRNA* sequences (Figure S2 and Table S3).

**Table 3:** Known miRNAs.

			expression levels^a^		genomic loci^b^		predicted targets
miR variants	miR sequence	length	root	leaf	miR	miR*	
phe-miRNA-156a	TGACAGAAGAGAGTGAGCAC	20	32094.04	3676.6418	9	2	**SBP-box gene family members**, putative NAM and protein homolog of At4g06744 precursor, and a hypothetical protein
phe-miRNA-156b	TTGACAGAAGAGAGTGAGCAC	21	32116.13	10555.14	3		
phe-miRNA-156c	TGACAGAAGAGAGTGGGCAC	20	1114.85	1.30795	1		
phe-miRNA-159a	TTTGGATTGAAGGGAGCTCTG	21	9014.519	6646.9916	4		**putative MYB transcription factors**, an unknown protein, and putative dnaJ protein and NB-ARC domain containing protein
phe-miRNA-159b	TTGGATTGAAGGGAGCTCTC	20	2612.538	396.30824	2	1	**putative MYB transcription factor** and phospholipase C
phe-miRNA-160	TGCCTGGCTCCCTGTATGCCA	21	480.6481	36.622543	10		**putative auxin response factors**, RNA polymerase sigma factor, histone-lysine N-methyltransferase, hydrolase and flap endonuclease, and hsp20/alpha crystallin family protein
phe-miRNA-162a	TCGATAAACCTCTGCATCCGG	21	3461.298	6721.5447	2		**putative Dicer** and phosphoesterase family protein, and B3 DNA binding domain containing protein
phe-miRNA-162b	TCGATAAACCTCTGCATCCAG	21	258.7296	642.20246	1		**putative Dicer** and C-5 cytosine-specific DNA methylase
phe-miRNA-164a	TGGAGAAGCAGGGCACGTGCA	21	491.1656	321.7552	8		**putative NAM protein**, Deg protease homologue and oligomeric Golgi complex component 3, PPR repeat domain containing protein, and an unknown protein
phe-miRNA-164b	TGGAGAAGCAGGGTACGTGCA	21	54.69082	1391.6567	4		**putative NAM protein,** GYF domain containing protein, Deg protease homologue and oligomeric Golgi complex component 3, and an unknown protein,
phe-miRNA-164c	TGGAGAAGCAGGGCACGTGAA	21	478.5446	2.615896	2		**putative NAM protein** and Deg protease homologue
phe-miRNA-166a	TCGGACCAGGCTTCATTCCCC	21	2155.028	527.10304	8	3	putative MATE efflux family protein and **homeodomain-leucine zipper family protei**n.
phe-miRNA-166b	TCTCGGACCAGGCTTCATTCC	21	512.2005	24.851012	3		target not found
phe-miRNA-166c	TCTCGGACTAGGCTTCATTCC	21	235.5912	3.9238439	1		target not found
phe-miRNA-167a	TGAAGCTGCCAGCATGATCTGA	22	1106.437	22981.954	5		**putative auxin response factors**
phe-miRNA-167b	TGAAGCTGCCAGCATGATCT	20	449.0957	319.13931	6		**putative auxin response factors** and dimerisation domain containing protein in Skp1 family
phe-miRNA-167c	TGAAGCTGCCAGCATGATCTGG	22	80.98448	987.5007	8		target not found
phe-miRNA-168	TCGCTTGGTGCAGATCGGGAC	21	30579.53	8786.7945	2	2	**putative PINHEAD and putative PAZ domain containing protein**
phe-miRNA-169a^c^	CAGCCAAGGATGACTTGCCGG	21	729.912	17.003324	4		target not found
phe-miRNA-169b^c^	CAGCCAAGGATGACTTGCCGC	21	585.8228	1143.1465	4	4	putative Sec1 family transport protein
phe-miRNA-171	TGATTGAGCCGTGCCAATATC	21	552.1669	58.857659	12		**putative scarecrow transcription factor family proteins**
phe-miRNA-319	TTGGACTGAAGGGTGCTCCCT	21	275.5576	388.46055	6		**putative TCP family transcription factors**
phe-miRNA-396a	TCCACAGGCTTTCTTGAACTG	21	1454.565	4172.3541	2		**putative growth regulating factor protein** and phosphatase family domain containing protein
phe-miRNA-396b	TTCCACAGCTTTCTTGAACTG	21	316.5757	270.74523	4		**putative growth regulating factor protein** and dynamin family protein, and an unknown protein
phe-miRNA-397	TCATTGAGTGCAGCGTTGATG	21	598.4437	51734.574	2		**putative laccase precursors**, RNA recognition containing protein, and calmodulin depedent protein kinase like protein, and cullulose synthase
phe-miRNA-398	TGTGTTCTCAGGTCACCCCTT	21	236.643	19.61922	1		target not found
phe-miRNA-399^c^	TGCCGAAGGAGAACTGCCCTG	21	178.7969	10.463584	1	1	**putative inorganic phosphate transporter**
phe-miRNA-408	TGCACTGCCTCTTCCCTGGC	20	41.01811	3027.8996	2	2	multicopper oxidase domain containing proteins, putative **plastocyanin-like domain containing protein**, acyl-protein thioesterase, PINHEAD, YABBY domain containing protein, ubiquitin carboxyl-terminal hydrolase domain containing protein, anthranilate phosphoribosyltransferase, RING-H2 finger protein, and unknown expressed proteins,
phe-miRNA-444a	TGCAGTTGTTGTCTCAAGCTT	21	89.39845	5328.5801	3		zinc finger C3HC4 type domain containing protein, putative DNA-directed RNA polymerase II subunit RPB9, EMB2261, chaperone protein clpB 1 and glycosyl hydrolase family 5 protein, and an unknown protein
phe-miRNA-444b	TGCAGTTGTTGCCTCAAGCTT	21	36.81113	1228.1632	1		zinc finger C3HC4 type domain containing protein and putative EMB2261, chaperone protein clpB 1, nodulin and integral membrane transporter family protein
phe-miRNA-528	TGGAAGGGGCATGCAGAGGAG	21	897.1397	83734.83	2		putative laccase precursor, plastocyanin-like domain containing protein, peroxidase precursor and polypheno oxidase
phe-miRNA-535	TGACAACGAGAGAGAGCACGC	21	7067.736	24309.521	6		**SBP-box gene family members**
phe-miRNA-827	TTAGATGACCATCAGCAAACA	21	431.216	149.10607	2		putative methyltransferase
phe-miRNA-1432	TTCAGGAGAGATGACACCGAC	21	39.96637	2610.6642	2		putative EF_hand_family_protein and plasma membrane type of calcium-transporting_ATPase_9
phe-miRNA-3979	CCTTCGGGGGAGGAGGGAAGC	21	318.6792	2.615896	1		puative PHLOEM 2-LIKE A10, phototropic-responsive NPH3 family protein and an unknown protein

a normalized read value;

b number of genomic loci;

c the most abundant sequence is the miR* sequence when the conserved small RNA sequence is chosen as mature miR sequence (Supplementary Table 2).

In root library, the most abundant sequences were phe-miRNA-156a, 156b and 168 with normalized read value around 30, 000 (Table 3). Their read numbers were about 3–4 times higher than in the leaf library where they also appeared to be accumulated at high levels. Other abundant miRNAs were phe-miRNA-156c, -159a, -159b, -162a, -166a, -167a, -169, -396a, and -535 with normalized read value above 1,000, and phe-miRNA-160, -162b, -164a, -164c, -166b, -166c, -167b, -169a, -169b, -171, -319, -396b, -397, -398, -399, -528, -827 and -3979 with read numbers above 100. Among these miRNAs, the read numbers of some miRNAs were below 20 in the leaf library such as phe-miRNA-399, -3979 and some variants of phe-miRNA-164 and 169, which were more than 40 to hundreds times lower than in the root libraries (Table 3 and Table S4).

In the leaf library, the most abundant sequences were phe-miRNA-156b, -167a, -397, 528 and -535 with normalized read value above 10,000. Particularly, the read numbers of phe-miRNA-528 and -397 were above 50,000, which were above 90 times more than in the root libraries. The total read numbers of these miRNAs took up 1.4% of the entire library. Other miRNAs with moderate levels are phe-miRNA-156a, -159a, -162a, -164b -168, -169b, -396a, -408, -444a, -444b, and -1432 with read numbers above 1,000; and phe-miRNA-159b, -162b, -164a, -166a, -167b, -167c, -319, -396b, and -827 with normalized read value above 100. Among these phe-miRNA-164b, -167a, -167b, -408, -444, and -1432 were much more abundant in leaf than in the root library. However, not all the variants in the same miRNA families showed a similar difference between the two libraries. Some miRNA variants show rather big differences in the two libraries such as phe-miRNA-167, -169 and -164 (Table 3 and Table S4). For example, we identified three variants of phe-miRNA-164 in both libraries, and both libraries contained similar amount of phe-miRNA-164a. However, the leaf library had more phe-miRNA-164b and root library contained more of phe-miRNA-164c.

### New miRNAs identified in Moso bamboo

In addition to the known miRNAs that have been identified in other species, we looked for new miRNAs that have not been described in any species. We searched the two libraries for potential new miRNAs using miRCat [25]. This algorithm identifies potential hairpin structures at the flanking regions of genome mapping sequences, considers the percentage of miRNA and miRNA* reads out of the total number of reads that map to the hairpin sequence (pre-miRNA sequence) and looks whether the expected miRNA* sequence has also been sequenced. Using the first two criteria, we identified seven potential new miRNAs with total normalized read value in the two libraries above 100 (Table 4 and Supplementary Figure 3). Since the miRNA* sequences of several conserved miRNAs were not present in our libraries, we included the two potential new miRNAs without sequenced miRNA* (New-6 and New-7) in Table 4 because their miRNA* may be present in future sRNA libraries. However, at present those two cannot be classified as miRNAs. The new miRNA, New-1, had high read numbers in both libraries. New-2 was more abundant in the root library while, New-3, New-4 and New-5 were more abundant in the leaf library. These five new miRNAs were also confirmed by Northern blot analysis and their expression profile matched the expected pattern based on the sequencing results (Figure 3).

**Table 4:** Putative new miRNAs.

			abundance*			
	sequence	length	root	leaf	genomic Location	miRNA star
New-1	GGCAAGTCTGTCCTTGGCTAC	21	4899.03	4478.41	PH01003459/60524–60544	CAGCCAAGGATGACTTGCCGC
					PH01000289/15663–15683	
					PH01000289/65843–65863	
					PH01000289/120711–120731	
New-2	GGCAGGTCTGTCCTTGGCTAC	21	2806.06	41.85	PH01000224/320759–320778	CAGCCAAGGAUGACUUGCCG
New-3	TCGTCGCAGGAGAGATGACGC	21	80.98	2727.07	PH01001122/158033–158053	TGGCGTCGTCTTCCTTGCGAC,GCGTCGTCTTCCTTGCGACGA
New-4	TGGGCGAGTCTTCTTGGCTATG	22	11.57	179.19	PH01002310/112516–112537	TTAGCCAAGAATGGCTTGCCTA,TAGCCAAGAATGGCTTGCCTAC
New-5	TCGGTTGCATTTGTAGTCCTA	21	10.52	274.67	PH01002844/54227–54247	TACGACTACAAATGCAACCGA
New-6	CTCCGAATTCTTGACAAACCGA	22	180.90	100.71	PH01001421/264619–264640	no
New-7	TTGCACTTGTCGACGGAGTTCC	22	46.28	149.11	PH01000975/466681–466702	no
					PH01205740/575–596	

*normalized read value.

### miRNA targets

The database of coding sequences from the published bamboo genome was used as input for the identification of putative targets of bamboo miRNAs. Many mRNAs involved in various biochemical procedures were predicted as targets for conserved miRNAs (Table 3, Supplementary Table 5). The majority of the predicted targets of the conserved miRNAs were also conserved and confirmed in other plant species (Table 3).

phe-miRNA-528, -397, -408, -444 and miRNA-1432 are the most differentially expressed miRNAs between the two libraries with higher accumulation in leaf tissues (Supplementary table 4). Among these, phe-miRNA-528, 444 and miRNA-1432 were found to be monocot-specific so far [23], [24]. Their predicted targets did not include the ones that were confirmed in other monocots [24], [26], [27] (Table 3). Nevertheless the putative targets of the two most abundant miRNAs, miRNA-528 and miRNA-397, include several family members of laccase precursors (Supplementary Table 5). In addition, the predicted targets of miRNA-528 and miRNA-408 include several plastocyanin-like domain containing proteins, multicopper oxidase domain containing proteins and polyphenol oxidase.

Using the current rules and coding sequence annotation, no putative targets were predicted for the new miRNAs.

### Other groups of sRNAs

We searched our sRNA libraries to identify the conserved miRNAs that target ta-siRNA precursors such as miRNA-390, miRNA-482 and miRNA-828. However, surprisingly we did not find any of these miRNAs in either of the two libraries. Next we tried to find the *MIRNA* genes for these miRNAs on the genome using maize miRNA-482, tomato miRNA-828 (because miRNA-828 has not been identified in any monocots so far) and bamboo miRNA-390 [8] sequences. We did not find a potential gene for miRNA-828 but it was expected as it seems to be dicot specific. The mature maize miR-482 sequence mapped to 435 positions on the bamboo genome (with 1 or 2 mis-matches); therefore we did not investigate whether any of them produces miRNA. However, we were able to identify a potential *MIRNA-390* gene. Next we tried to detect phe-miRNA-390 by northern blot analysis but our attempt was not successful (Figure 3), therefore we did not look for ta-siRNAs.

In addition to the sRNAs that were mapped to the well-characterized non-coding and coding sequences about 37% of top 100 reads in root library and about 11% in leaf library were mapped to intergenic or intron region. Among them, a series of sRNAs ranging from 30–32 nucleotides were mapped to 14 different loci in the bamboo genome. One of them is the most abundant sRNA in the root library which contributes more than 50% of 31 mer reads. The sum of their reads is about 16% of the total genome matching reads in the root library. Using a probe complementary to the most abundant 31 nt sRNA, Northern blot analysis revealed a very strong signal in the root sample (Figure 3). Accumulation of this sRNA was also confirmed in stem and leaf tissues.

## Discussion

In the present study, we have analysed the sRNA profiles in leaf and root tissues of moso bamboo, one of the most important forest grass. Several groups of sRNAs with known and unknown functions were found in the sRNA libraries constructed by using HD adapters. The accumulation of several sRNAs was verified in the bamboo seedlings by northern blot analysis. The expression patterns obtained by Northern blot analysis were consistent with the sequencing results indicating the excellent quantitative correlation between sRNA read numbers and its actual accumulation in two different tissue samples. Thus, the HD adapters based cDNA libraries appeared to be reliable for high through analysis of sRNA populations in plant tissues.

### Complex sRNA population in plant tissues

As in most plant sRNA libraries, 21 mer miRNAs and 24 mer siRNAs were very abundant in the bamboo leaf and root sRNA libraries. However, there were also differences between the two tissues. For example, 25% of the sRNAs in the leaf library were mapped to the chloroplast genome while very few reads in the root library were derived from plastid. There is an obvious difference between plastid development and chloroplast activity between the tissues, which is also reflected at the sRNA level. A large number of sRNAs were mapped to non-coding RNAs such as rRNAs and tRNAs and UTRs or intergenic regions with extremely high redundancy. Many of them were mapped to the PPR binding or predicted PPR binding sites based on results in other plant species indicating a valuable resource for future PPR binding site prediction. The different sizes of sequences or accumulation of these sRNAs in the two libraries may reflect the underlying mechanisms in transcription or translation regulation of plastid originated genes in these tissues.

In most previously published papers on sRNA profiling, the proportion of rRNA/tRNA matching sRNAs has been smaller than what we have found. However, the majority of the total RNA consists of rRNAs and tRNAs are also very abundant. Nevertheless it will be interesting to understand in the future why some sRNAs with high abundance were mapped to specific positions of specific rRNAs or tRNAs. In addition, there are some other highly abundant sRNAs that were mapped to intergenic or intron regions and their accumulation levels also varied in the two tissues. Further study of their origins and functions will help to understand sRNA biology in plants.

However, it is unexpected that none of the known and conserved TAS RNA targeting miRNAs were found or detected in the leaf and root tissues of young bamboo seedlings. miRNA-390 was cloned in the culm libraries of moso bamboo with relatively low normalized read value [8], but not other miRNAs such as miRNA482 and miRNA828. These two miRNAs were not found in rice either.

### The miRNA profiles are consistent with their biological functions

A set of known conserved miRNAs was found in both libraries in high abundance. Their predicted targets were also conserved or confirmed in other plant species. These conserved miRNAs are known to be important for leaf and root development by regulating the expression of transcription factors involved in organ development such as miRNA-156, -167, -166, -159, -169, -319, -164, -160, -444, -171, -396 and miRNA-535. For example, both miRNA-160 and miRNA-164 are required for root development in Arabidopsis [28], [29] while miRNA-164 is also required for vegetative shoot development [30], [31]. Along with miRNA-319, miRNA-164 control leaf senescence [32], [33]. mir319 also regulates cell proliferation and leaf morphogenesis mediated by the conserved miR396–GRF module [34]. Mir159 is essential for leaf morphogenesis [35], [36] and miRNA-167 plays a role in the development of adventitious root in both dicots and monocots [37]–[39].

Some of the above conserved miRNAs and other conserved miRNAs such as miRNA-398, -399, -168 and -162 also respond to environmental stresses and regulate nutrient uptake in both leaf and root tissues [26], [40]–[45]. Although these miRNAs were expressed abundantly in both root and leaf tissues, the variants of some miRNA families exhibited tissue preference, which may be correlated with some tissue specific targets and/or tissue specific regulation under environmental stresses. Some miRNAs exhibit quite dramatic expression difference between the two tissues. For example, phe-miRNA-156 was more abundant in roots particularly one of its variants. Over-expressing of tomato miRNA-156 induces the development of high density of airy roots along the stem [46]. The higher accumulation of bamboo miRNA-156 may be correlated with its dense adventitious roots and airy roots along the stem which help with its growth in poor soil. On the other hand miRNA-528, -397 and -408 were expressed at much higher levels in bamboo leaf tissue. miRNA-528 is also very abundant in rice seedlings and young maize leaf tissues [47], [48]. It is expressed in maize shoot apex and leaf primordial and vasculature [49]. Through qRT-PCR, miRNA 397 and miRNA 528 were found to most abundant in the leaves of *Pinellia pedatisecta S.* [50]. The experimentally confirmed target in rice is Os06g37150 encoding an ascorbate oxidase [27], and IAA-alanine resistance protein 1 (IAR1) gene [26]. One confirmed target in maize is copper/zinc superoxide dismutase [44]. However, none of these genes’ homologs were predicted to be the targets of bamboo miRNA-528. Instead, phe-miRNA-528 more likely targets polyphenol oxidase genes including the laccase genes, another putative polyphenol oxidase, a putative peroxidase and plastocyanin-like proteins. In the meantime, miRNA-397 and miRNA-408 were predicted to target different laccases and a plastocyanin-like protein and other polyphenol oxidases, respectively. Both polyphenol oxidases and plastocyanin proteins are copper containing oxidases. miRNA-397 has been confirmed to be involved in cell wall development by down-regulating laccases in Arabidopsis and poplar [51]. Both miRNA-528 and miRNA-397 also respond to diverse biotic and abiotic stresses including low copper availability [26], [40], [41], [43]. Thus these three miRNAs that were preferentially expressed in leaf tissues may work together in regulating stress response and copper homeostasis. For example, they may down regulate the expression of copper-containing proteins to accommodate the optimum availability of copper for photosynthesis, in order to store enough energy for rapid growth.

## Supporting Information

Figure S1 Size and complexity distributions of various groups of sRNAs.
(TIF)

Figure S2 Predicted hairpin structures for all the identified known miRNAs and other general information.
(PPTX)

Figure S3 Predicted hairpin structures for all the predicted new miRNAs and other general information.
(PPTX)

Table S1 Sequences of the probes used for Northern blot analysis.
(XLSX)

Table S2 List of all the miRNAs and miRNA* that were identified through similarity search.
(XLSX)

Table S3 List of all the genomic loci of the identified miRNAs and miRNA* and the corresponding hairpin sequences.
(XLSX)

Table S4 Comparison of the abundance of all the miRNAs in leaf and root libraries.
(XLSX)

Table S5 List of the predicted targets of all known miRNAs identified in leaf and root libraries.
(XLSX)
