# Supplementary figures and images for: Small RNA Profile in Moso Bamboo Root and Leaf Obtained by High Definition Adapters
Source: PLoS One. 2014 Jul 31;9(7):e103590. doi: 10.1371/journal.pone.0103590 (PMC4117519; doi:10.1371/journal.pone.0103590)

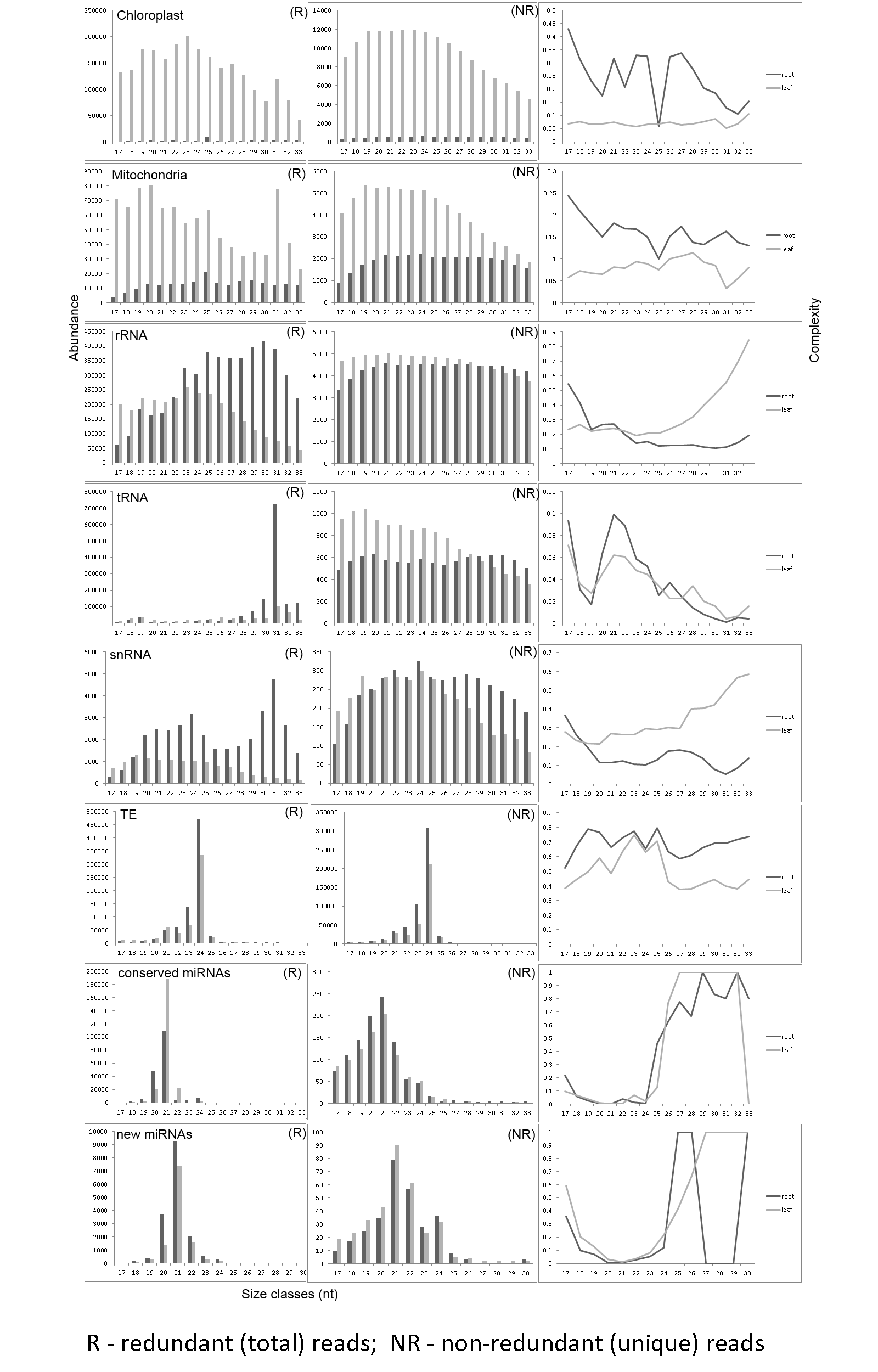

Supplement: Figure S1 — Size and complexity distributions of various groups of sRNAs. (TIF) [file pone.0103590.s001.tif]
