# Supplementary material for: Small RNA Profile in Moso Bamboo Root and Leaf Obtained by High Definition Adapters
Source: PLoS One. 2014 Jul 31;9(7):e103590. doi: 10.1371/journal.pone.0103590 (PMC4117519; doi:10.1371/journal.pone.0103590)

## Slide 1
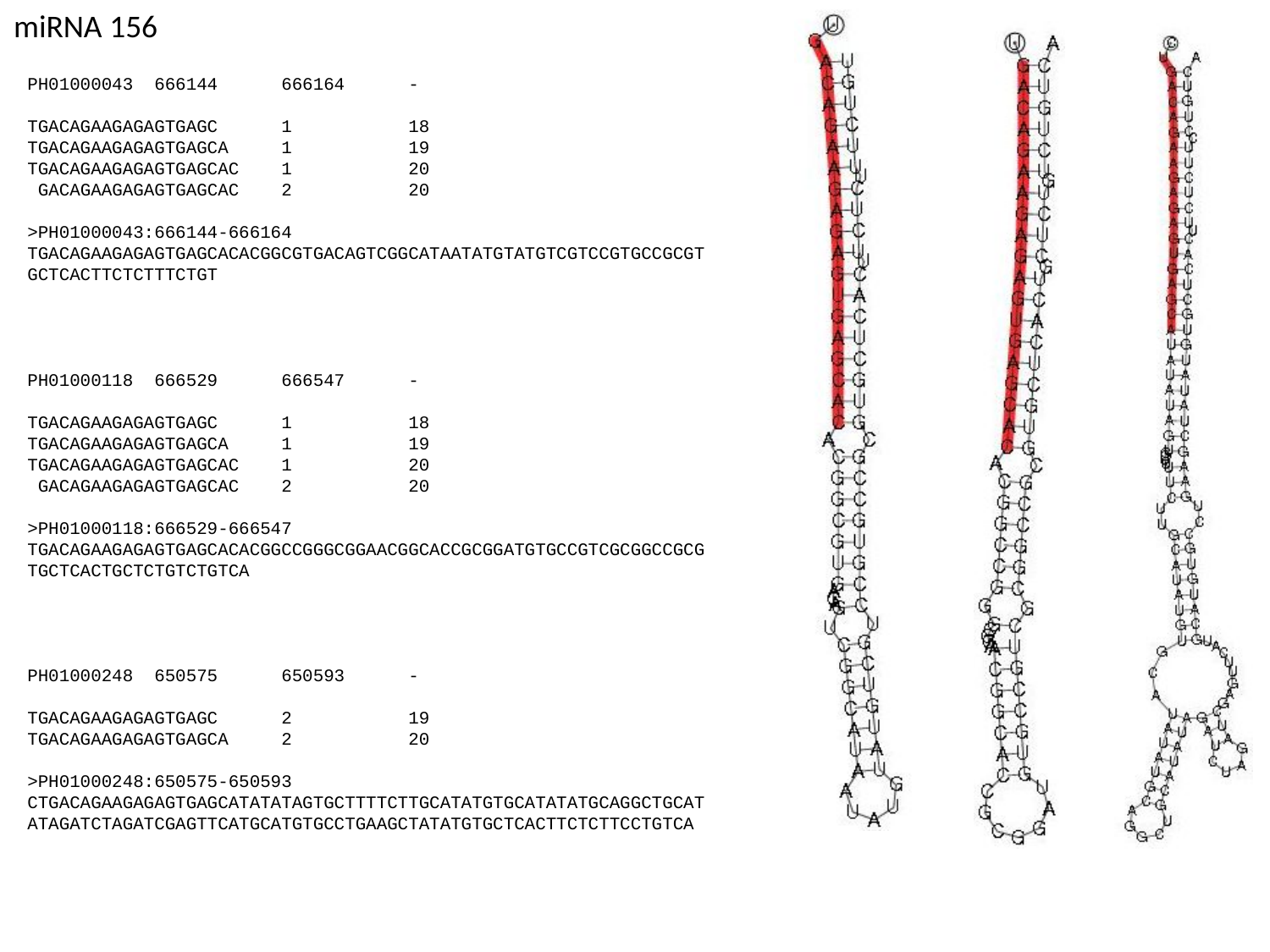

miRNA 156
PH01000043	666144	666164	-
TGACAGAAGAGAGTGAGC 	1	18
TGACAGAAGAGAGTGAGCA 	1	19
TGACAGAAGAGAGTGAGCAC 	1	20
 GACAGAAGAGAGTGAGCAC 	2	20
>PH01000043:666144-666164
TGACAGAAGAGAGTGAGCACACGGCGTGACAGTCGGCATAATATGTATGTCGTCCGTGCCGCGTGCTCACTTCTCTTTCTGT
PH01000118	666529	666547	-
TGACAGAAGAGAGTGAGC 	1	18
TGACAGAAGAGAGTGAGCA 	1	19
TGACAGAAGAGAGTGAGCAC 	1	20
 GACAGAAGAGAGTGAGCAC 	2	20
>PH01000118:666529-666547
TGACAGAAGAGAGTGAGCACACGGCCGGGCGGAACGGCACCGCGGATGTGCCGTCGCGGCCGCGTGCTCACTGCTCTGTCTGTCA
PH01000248	650575	650593	-
TGACAGAAGAGAGTGAGC 	2	19
TGACAGAAGAGAGTGAGCA 	2	20
>PH01000248:650575-650593
CTGACAGAAGAGAGTGAGCATATATAGTGCTTTTCTTGCATATGTGCATATATGCAGGCTGCATATAGATCTAGATCGAGTTCATGCATGTGCCTGAAGCTATATGTGCTCACTTCTCTTCCTGTCA

## Slide 2
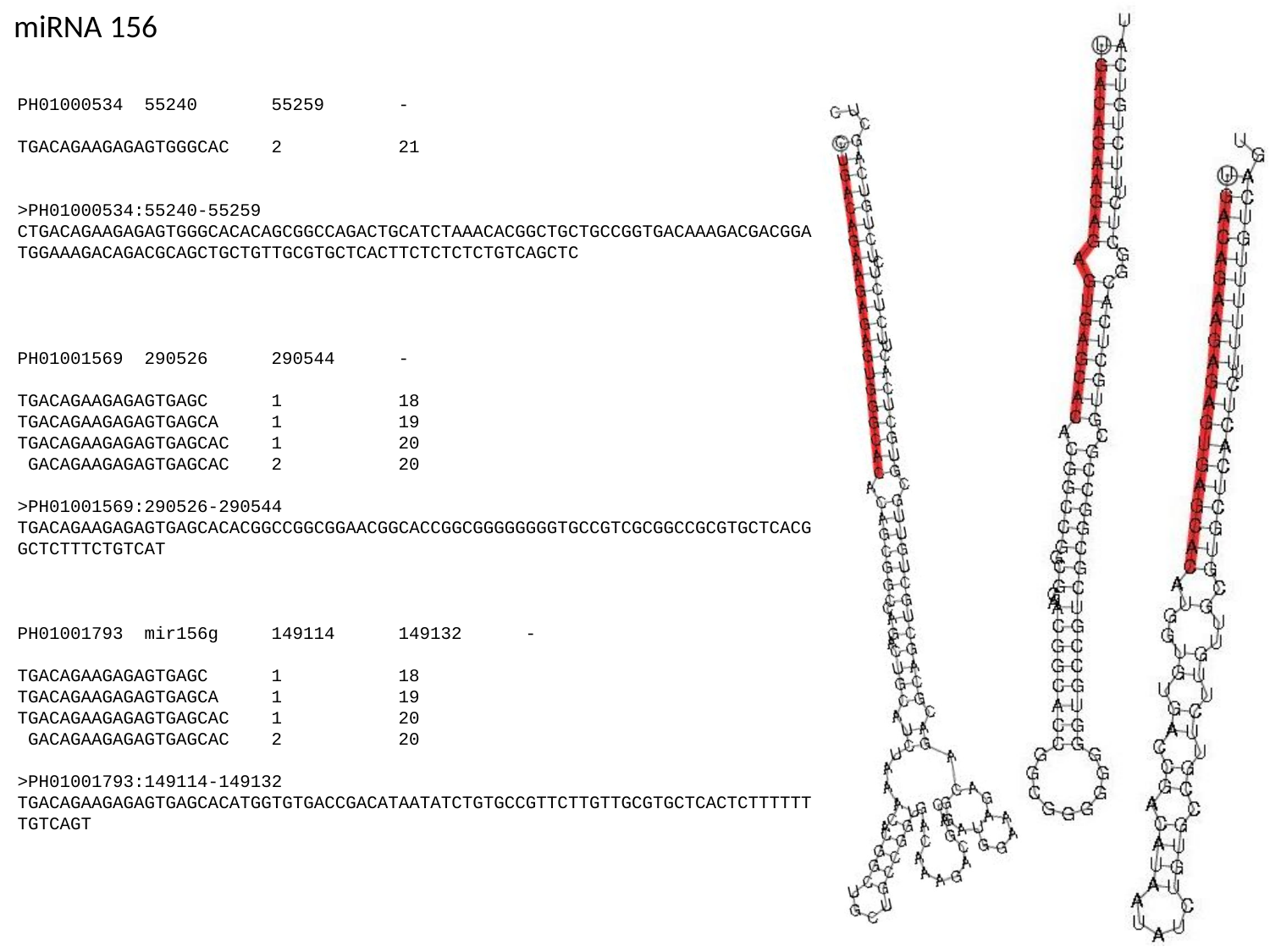

miRNA 156
PH01000534	55240	55259	-
TGACAGAAGAGAGTGGGCAC 	2	21
>PH01000534:55240-55259
CTGACAGAAGAGAGTGGGCACACAGCGGCCAGACTGCATCTAAACACGGCTGCTGCCGGTGACAAAGACGACGGATGGAAAGACAGACGCAGCTGCTGTTGCGTGCTCACTTCTCTCTCTGTCAGCTC
PH01001569	290526	290544	-
TGACAGAAGAGAGTGAGC 	1	18
TGACAGAAGAGAGTGAGCA 	1	19
TGACAGAAGAGAGTGAGCAC 	1	20
 GACAGAAGAGAGTGAGCAC 	2	20
>PH01001569:290526-290544
TGACAGAAGAGAGTGAGCACACGGCCGGCGGAACGGCACCGGCGGGGGGGGTGCCGTCGCGGCCGCGTGCTCACGGCTCTTTCTGTCAT
PH01001793	mir156g 	149114	149132	-
TGACAGAAGAGAGTGAGC 	1	18
TGACAGAAGAGAGTGAGCA 	1	19
TGACAGAAGAGAGTGAGCAC 	1	20
 GACAGAAGAGAGTGAGCAC 	2	20
>PH01001793:149114-149132
TGACAGAAGAGAGTGAGCACATGGTGTGACCGACATAATATCTGTGCCGTTCTTGTTGCGTGCTCACTCTTTTTTTGTCAGT

## Slide 3
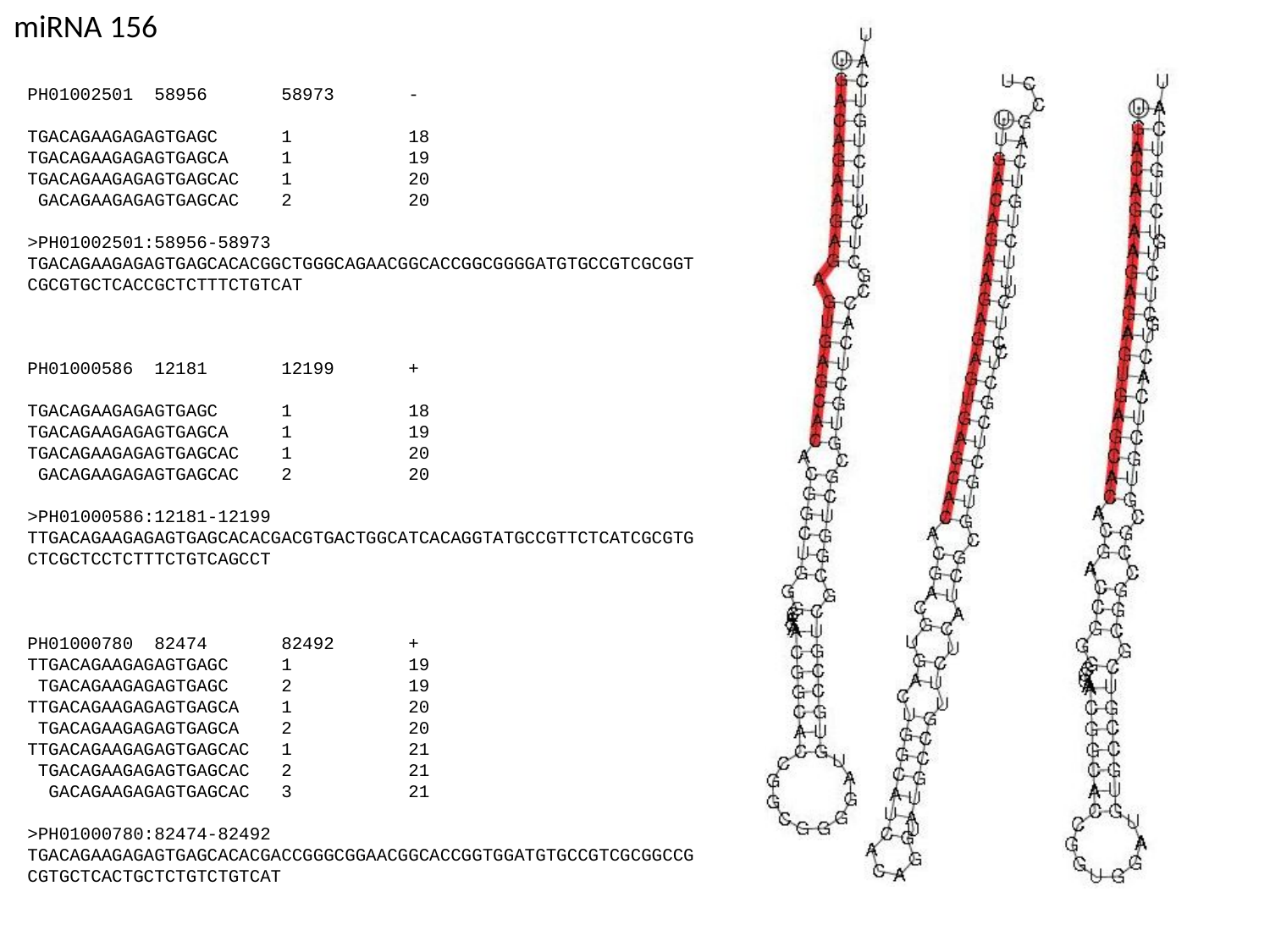

miRNA 156
PH01002501	58956	58973	-
TGACAGAAGAGAGTGAGC 	1	18
TGACAGAAGAGAGTGAGCA 	1	19
TGACAGAAGAGAGTGAGCAC 	1	20
 GACAGAAGAGAGTGAGCAC 	2	20
>PH01002501:58956-58973
TGACAGAAGAGAGTGAGCACACGGCTGGGCAGAACGGCACCGGCGGGGATGTGCCGTCGCGGTCGCGTGCTCACCGCTCTTTCTGTCAT
PH01000586	12181	12199	+
TGACAGAAGAGAGTGAGC 	1	18
TGACAGAAGAGAGTGAGCA 	1	19
TGACAGAAGAGAGTGAGCAC 	1	20
 GACAGAAGAGAGTGAGCAC 	2	20
>PH01000586:12181-12199
TTGACAGAAGAGAGTGAGCACACGACGTGACTGGCATCACAGGTATGCCGTTCTCATCGCGTGCTCGCTCCTCTTTCTGTCAGCCT
PH01000780	82474	82492	+
TTGACAGAAGAGAGTGAGC 	1	19
 TGACAGAAGAGAGTGAGC 	2	19
TTGACAGAAGAGAGTGAGCA 	1	20
 TGACAGAAGAGAGTGAGCA 	2	20
TTGACAGAAGAGAGTGAGCAC 	1	21
 TGACAGAAGAGAGTGAGCAC 	2	21
 GACAGAAGAGAGTGAGCAC 	3	21
>PH01000780:82474-82492 TGACAGAAGAGAGTGAGCACACGACCGGGCGGAACGGCACCGGTGGATGTGCCGTCGCGGCCGCGTGCTCACTGCTCTGTCTGTCAT

## Slide 4
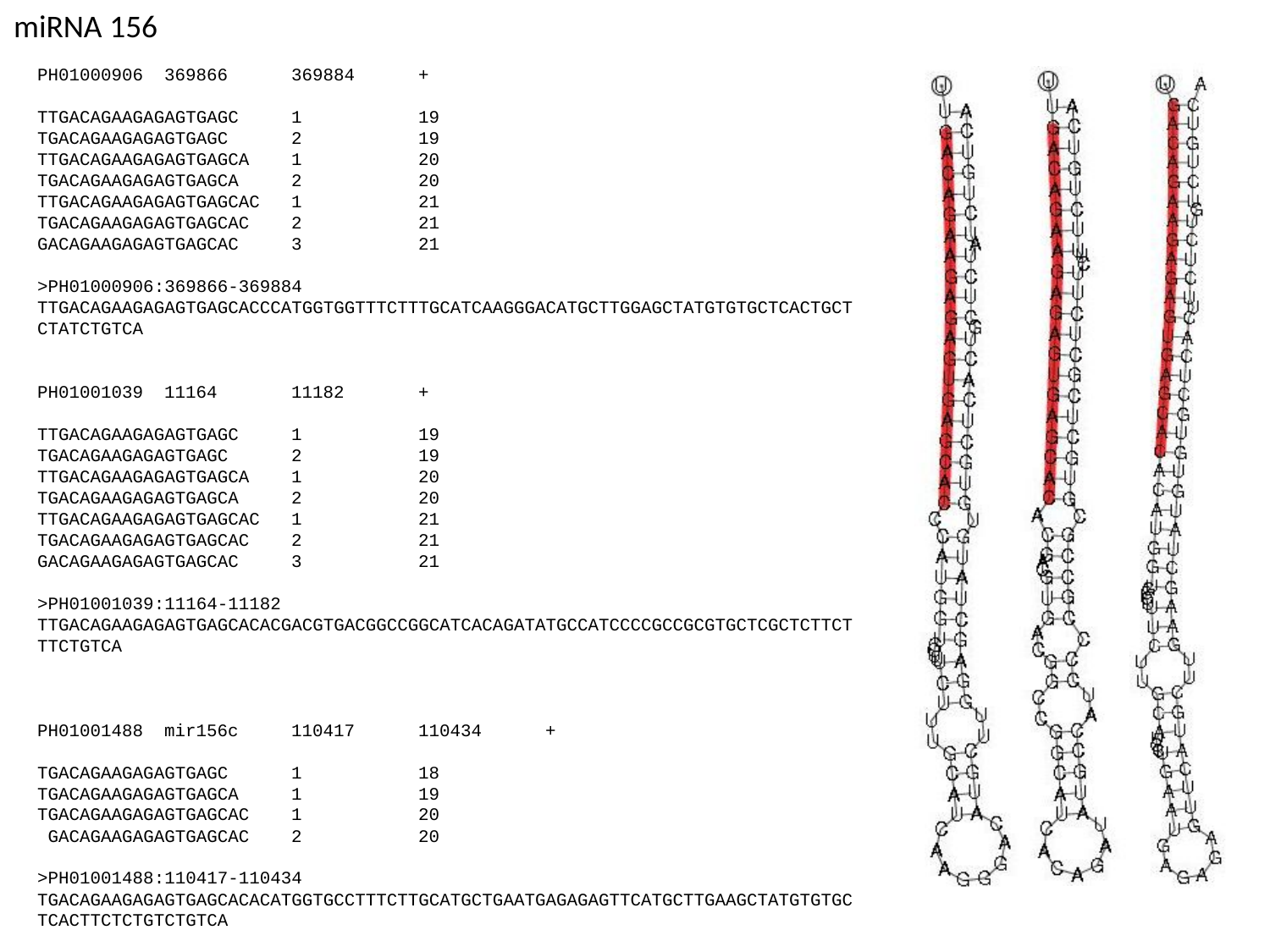

miRNA 156
PH01000906	369866	369884	+
TTGACAGAAGAGAGTGAGC 	1	19
TGACAGAAGAGAGTGAGC 	2	19
TTGACAGAAGAGAGTGAGCA 	1	20
TGACAGAAGAGAGTGAGCA 	2	20
TTGACAGAAGAGAGTGAGCAC 	1	21
TGACAGAAGAGAGTGAGCAC 	2	21
GACAGAAGAGAGTGAGCAC 	3	21
>PH01000906:369866-369884
TTGACAGAAGAGAGTGAGCACCCATGGTGGTTTCTTTGCATCAAGGGACATGCTTGGAGCTATGTGTGCTCACTGCTCTATCTGTCA
PH01001039	11164	11182	+
TTGACAGAAGAGAGTGAGC 	1	19
TGACAGAAGAGAGTGAGC 	2	19
TTGACAGAAGAGAGTGAGCA 	1	20
TGACAGAAGAGAGTGAGCA 	2	20
TTGACAGAAGAGAGTGAGCAC 	1	21
TGACAGAAGAGAGTGAGCAC 	2	21
GACAGAAGAGAGTGAGCAC 	3	21
>PH01001039:11164-11182
TTGACAGAAGAGAGTGAGCACACGACGTGACGGCCGGCATCACAGATATGCCATCCCCGCCGCGTGCTCGCTCTTCTTTCTGTCA
PH01001488	mir156c 	110417	110434	+
TGACAGAAGAGAGTGAGC 	1	18
TGACAGAAGAGAGTGAGCA 	1	19
TGACAGAAGAGAGTGAGCAC 	1	20
 GACAGAAGAGAGTGAGCAC 	2	20
>PH01001488:110417-110434
TGACAGAAGAGAGTGAGCACACATGGTGCCTTTCTTGCATGCTGAATGAGAGAGTTCATGCTTGAAGCTATGTGTGCTCACTTCTCTGTCTGTCA

## Slide 5
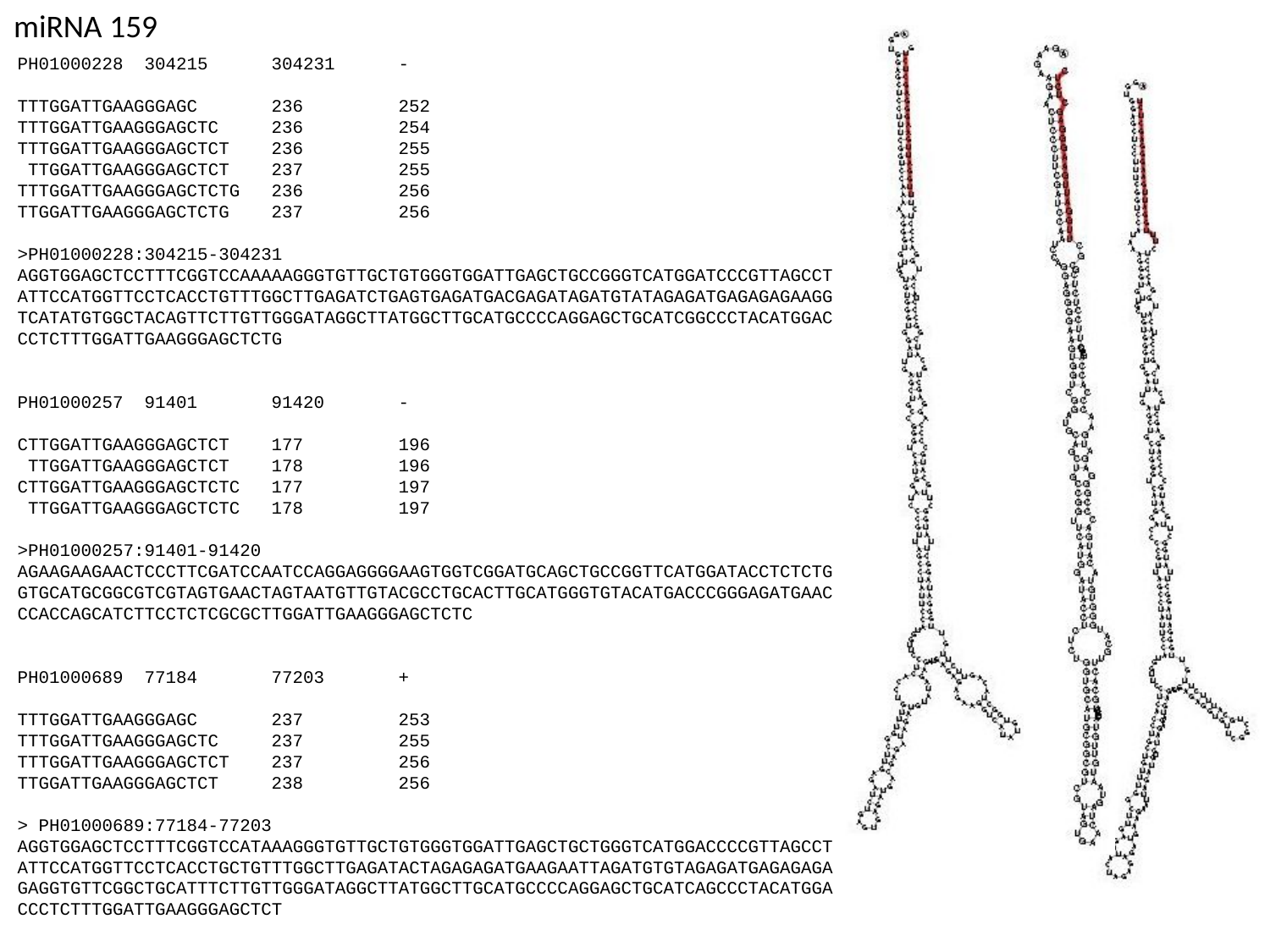

miRNA 159
PH01000228	304215	304231	-
TTTGGATTGAAGGGAGC 	236	252
TTTGGATTGAAGGGAGCTC 	236	254
TTTGGATTGAAGGGAGCTCT 	236	255
 TTGGATTGAAGGGAGCTCT 	237	255
TTTGGATTGAAGGGAGCTCTG 	236	256
TTGGATTGAAGGGAGCTCTG 	237	256
>PH01000228:304215-304231
AGGTGGAGCTCCTTTCGGTCCAAAAAGGGTGTTGCTGTGGGTGGATTGAGCTGCCGGGTCATGGATCCCGTTAGCCTATTCCATGGTTCCTCACCTGTTTGGCTTGAGATCTGAGTGAGATGACGAGATAGATGTATAGAGATGAGAGAGAAGGTCATATGTGGCTACAGTTCTTGTTGGGATAGGCTTATGGCTTGCATGCCCCAGGAGCTGCATCGGCCCTACATGGACCCTCTTTGGATTGAAGGGAGCTCTG
PH01000257	91401	91420	-
CTTGGATTGAAGGGAGCTCT 	177	196
 TTGGATTGAAGGGAGCTCT 	178	196
CTTGGATTGAAGGGAGCTCTC 	177	197
 TTGGATTGAAGGGAGCTCTC 	178	197
>PH01000257:91401-91420
AGAAGAAGAACTCCCTTCGATCCAATCCAGGAGGGGAAGTGGTCGGATGCAGCTGCCGGTTCATGGATACCTCTCTGGTGCATGCGGCGTCGTAGTGAACTAGTAATGTTGTACGCCTGCACTTGCATGGGTGTACATGACCCGGGAGATGAACCCACCAGCATCTTCCTCTCGCGCTTGGATTGAAGGGAGCTCTC
PH01000689	77184	77203	+
TTTGGATTGAAGGGAGC 	237	253
TTTGGATTGAAGGGAGCTC 	237	255
TTTGGATTGAAGGGAGCTCT 	237	256
TTGGATTGAAGGGAGCTCT 	238	256
> PH01000689:77184-77203
AGGTGGAGCTCCTTTCGGTCCATAAAGGGTGTTGCTGTGGGTGGATTGAGCTGCTGGGTCATGGACCCCGTTAGCCTATTCCATGGTTCCTCACCTGCTGTTTGGCTTGAGATACTAGAGAGATGAAGAATTAGATGTGTAGAGATGAGAGAGAGAGGTGTTCGGCTGCATTTCTTGTTGGGATAGGCTTATGGCTTGCATGCCCCAGGAGCTGCATCAGCCCTACATGGACCCTCTTTGGATTGAAGGGAGCTCT

## Slide 6
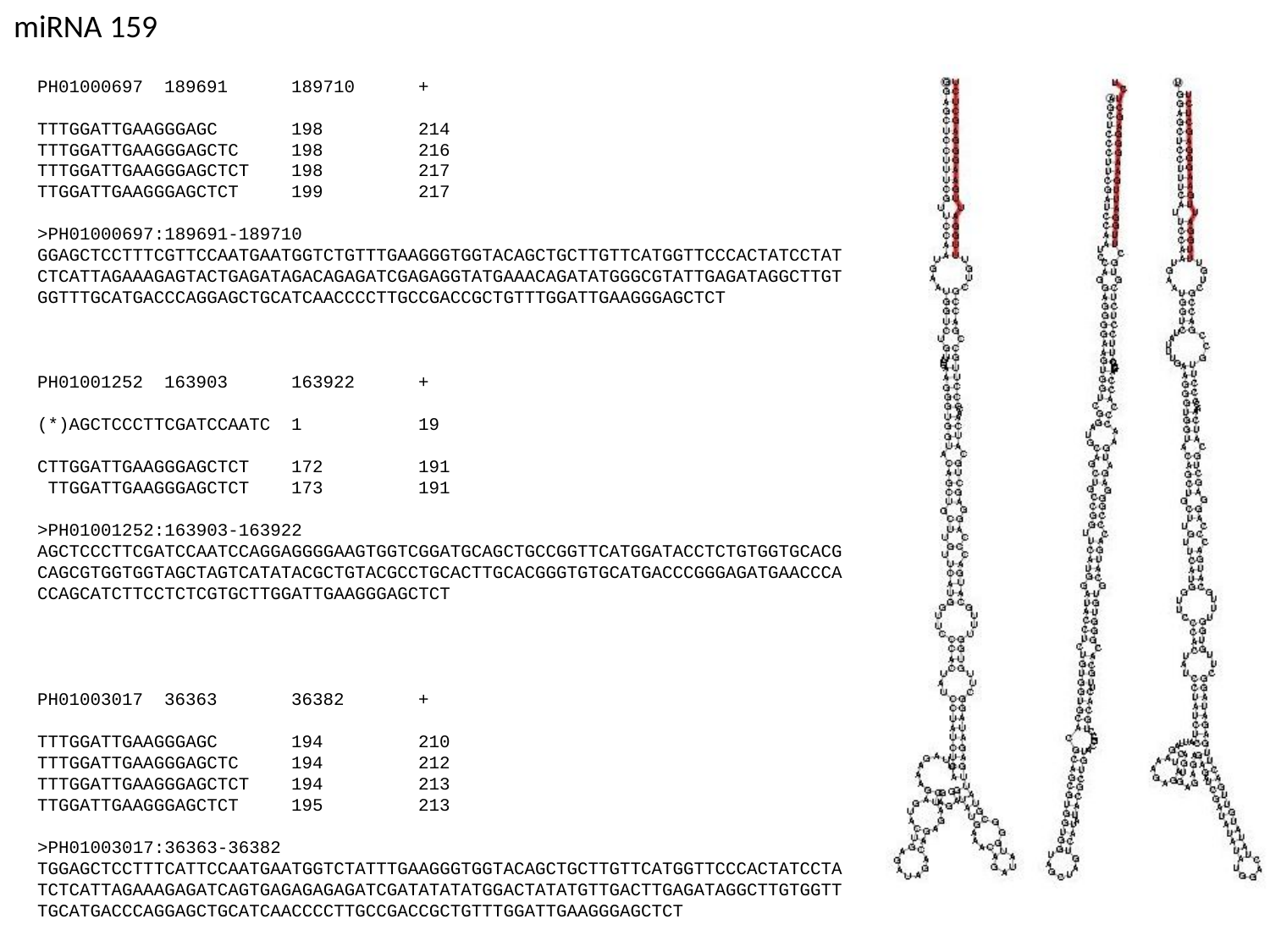

miRNA 159
PH01000697	189691	189710	+
TTTGGATTGAAGGGAGC 	198	214
TTTGGATTGAAGGGAGCTC 	198	216
TTTGGATTGAAGGGAGCTCT 	198	217
TTGGATTGAAGGGAGCTCT 	199	217
>PH01000697:189691-189710
GGAGCTCCTTTCGTTCCAATGAATGGTCTGTTTGAAGGGTGGTACAGCTGCTTGTTCATGGTTCCCACTATCCTATCTCATTAGAAAGAGTACTGAGATAGACAGAGATCGAGAGGTATGAAACAGATATGGGCGTATTGAGATAGGCTTGTGGTTTGCATGACCCAGGAGCTGCATCAACCCCTTGCCGACCGCTGTTTGGATTGAAGGGAGCTCT
PH01001252	163903	163922	+
(*)AGCTCCCTTCGATCCAATC 	1	19
CTTGGATTGAAGGGAGCTCT 	172	191
 TTGGATTGAAGGGAGCTCT 	173	191
>PH01001252:163903-163922
AGCTCCCTTCGATCCAATCCAGGAGGGGAAGTGGTCGGATGCAGCTGCCGGTTCATGGATACCTCTGTGGTGCACGCAGCGTGGTGGTAGCTAGTCATATACGCTGTACGCCTGCACTTGCACGGGTGTGCATGACCCGGGAGATGAACCCACCAGCATCTTCCTCTCGTGCTTGGATTGAAGGGAGCTCT
PH01003017	36363	36382	+
TTTGGATTGAAGGGAGC 	194	210
TTTGGATTGAAGGGAGCTC 	194	212
TTTGGATTGAAGGGAGCTCT 	194	213
TTGGATTGAAGGGAGCTCT 	195	213
>PH01003017:36363-36382
TGGAGCTCCTTTCATTCCAATGAATGGTCTATTTGAAGGGTGGTACAGCTGCTTGTTCATGGTTCCCACTATCCTATCTCATTAGAAAGAGATCAGTGAGAGAGAGATCGATATATATGGACTATATGTTGACTTGAGATAGGCTTGTGGTTTGCATGACCCAGGAGCTGCATCAACCCCTTGCCGACCGCTGTTTGGATTGAAGGGAGCTCT

## Slide 7
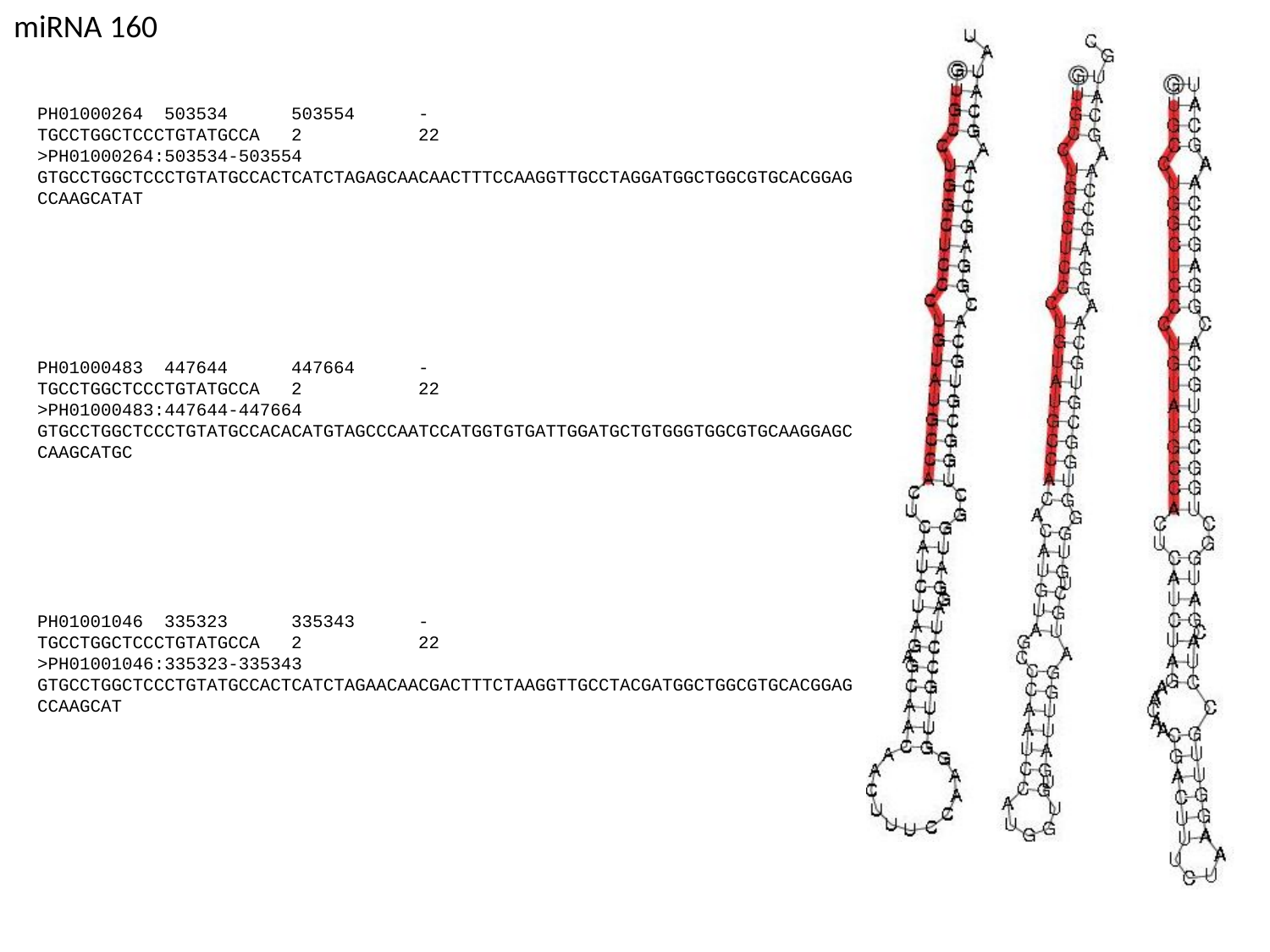

miRNA 160
PH01000264	503534	503554	-
TGCCTGGCTCCCTGTATGCCA 	2	22
>PH01000264:503534-503554
GTGCCTGGCTCCCTGTATGCCACTCATCTAGAGCAACAACTTTCCAAGGTTGCCTAGGATGGCTGGCGTGCACGGAGCCAAGCATAT
PH01000483	447644	447664	-
TGCCTGGCTCCCTGTATGCCA 	2	22
>PH01000483:447644-447664
GTGCCTGGCTCCCTGTATGCCACACATGTAGCCCAATCCATGGTGTGATTGGATGCTGTGGGTGGCGTGCAAGGAGCCAAGCATGC
PH01001046 	335323	335343	-
TGCCTGGCTCCCTGTATGCCA 	2	22
>PH01001046:335323-335343
GTGCCTGGCTCCCTGTATGCCACTCATCTAGAACAACGACTTTCTAAGGTTGCCTACGATGGCTGGCGTGCACGGAGCCAAGCAT

## Slide 8
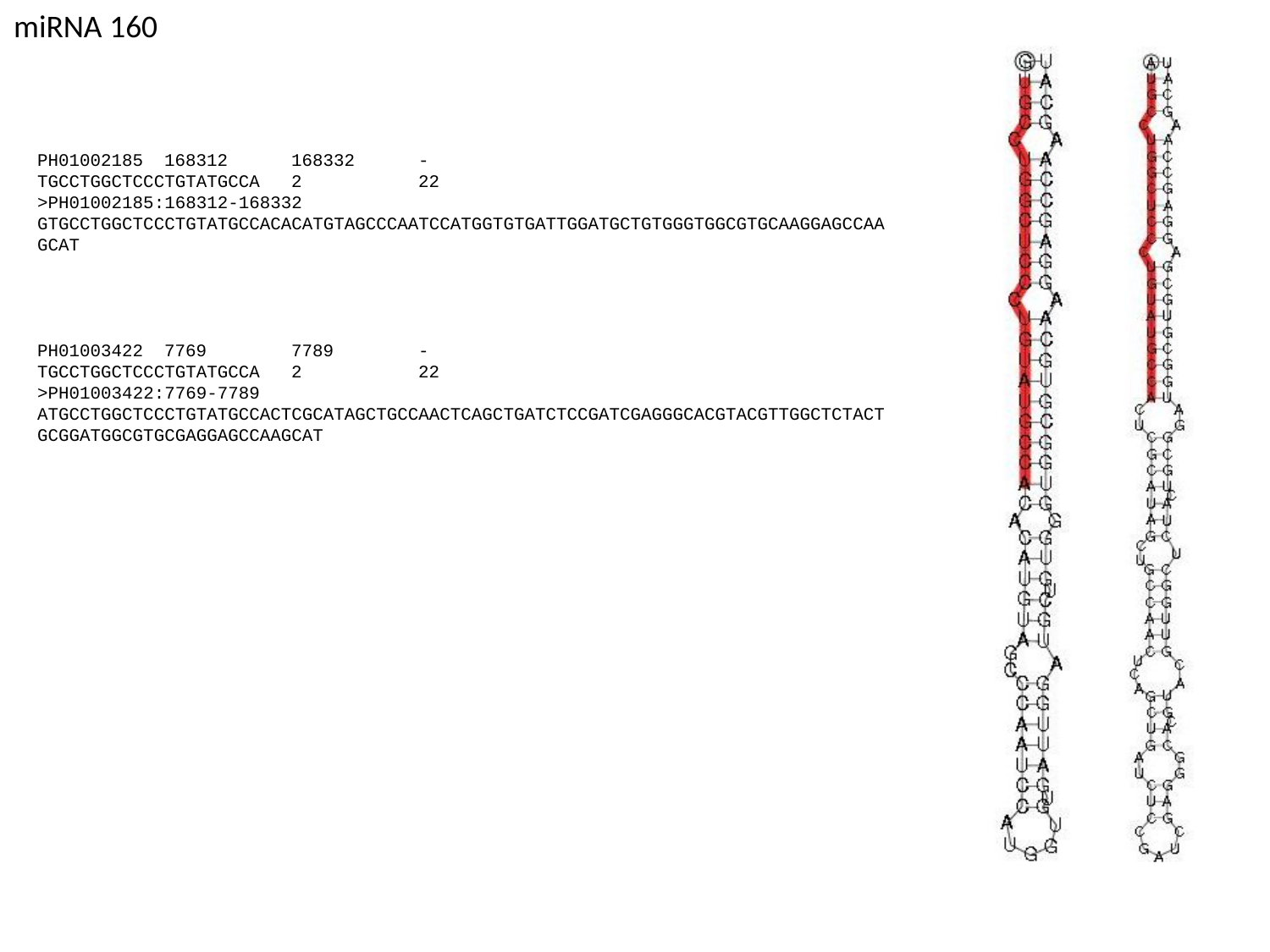

miRNA 160
PH01002185	168312	168332	-
TGCCTGGCTCCCTGTATGCCA 	2	22
>PH01002185:168312-168332
GTGCCTGGCTCCCTGTATGCCACACATGTAGCCCAATCCATGGTGTGATTGGATGCTGTGGGTGGCGTGCAAGGAGCCAAGCAT
PH01003422	7769	7789	-
TGCCTGGCTCCCTGTATGCCA 	2	22
>PH01003422:7769-7789
ATGCCTGGCTCCCTGTATGCCACTCGCATAGCTGCCAACTCAGCTGATCTCCGATCGAGGGCACGTACGTTGGCTCTACTGCGGATGGCGTGCGAGGAGCCAAGCAT

## Slide 9
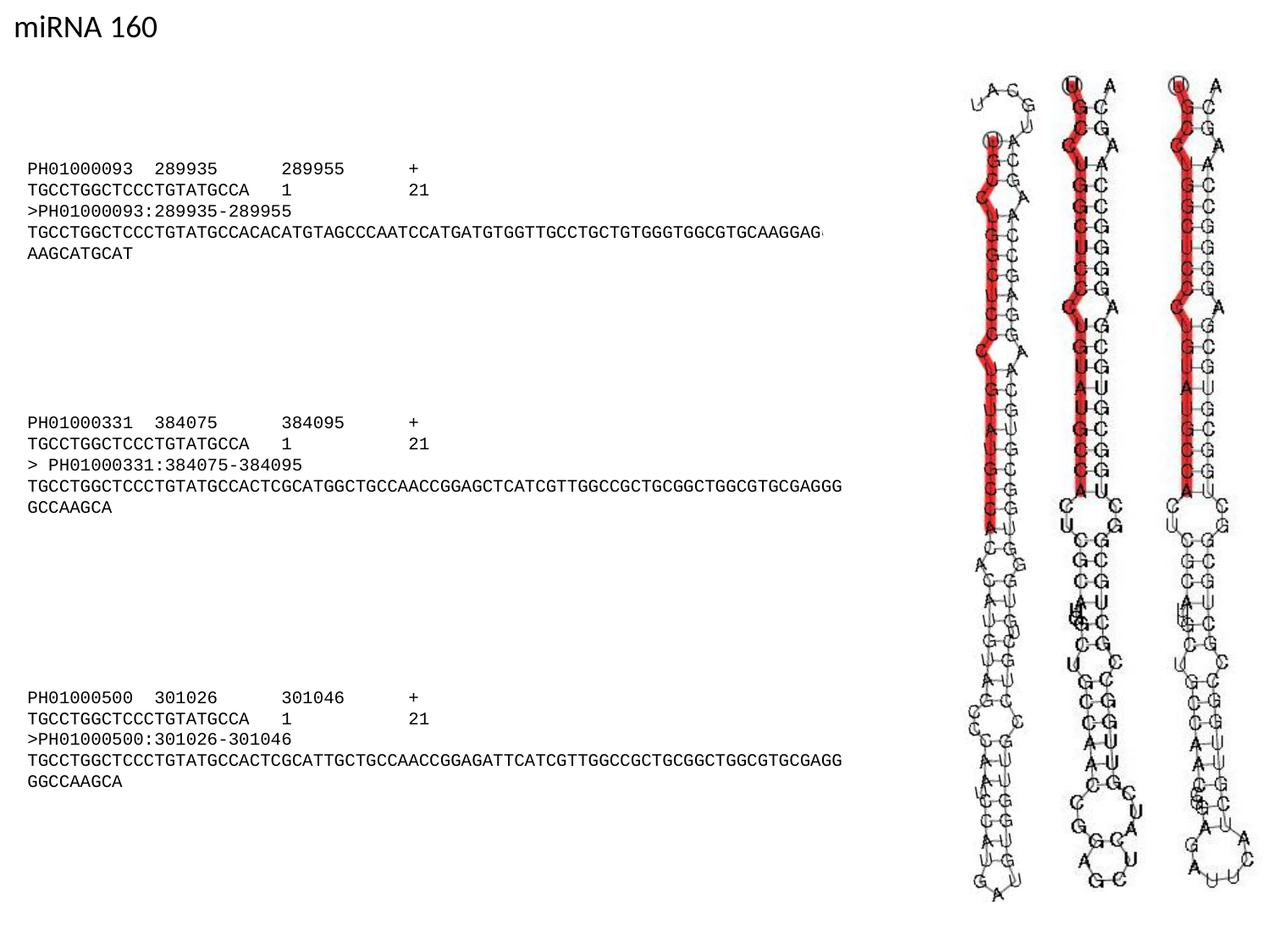

miRNA 160
PH01000093 	289935	289955	+
TGCCTGGCTCCCTGTATGCCA 	1	21
>PH01000093:289935-289955
TGCCTGGCTCCCTGTATGCCACACATGTAGCCCAATCCATGATGTGGTTGCCTGCTGTGGGTGGCGTGCAAGGAGCCAAGCATGCAT
PH01000331 	384075	384095	+
TGCCTGGCTCCCTGTATGCCA 	1	21
> PH01000331:384075-384095
TGCCTGGCTCCCTGTATGCCACTCGCATGGCTGCCAACCGGAGCTCATCGTTGGCCGCTGCGGCTGGCGTGCGAGGGGCCAAGCA
PH01000500 	301026	301046	+
TGCCTGGCTCCCTGTATGCCA 	1	21
>PH01000500:301026-301046
TGCCTGGCTCCCTGTATGCCACTCGCATTGCTGCCAACCGGAGATTCATCGTTGGCCGCTGCGGCTGGCGTGCGAGGGGCCAAGCA

## Slide 10
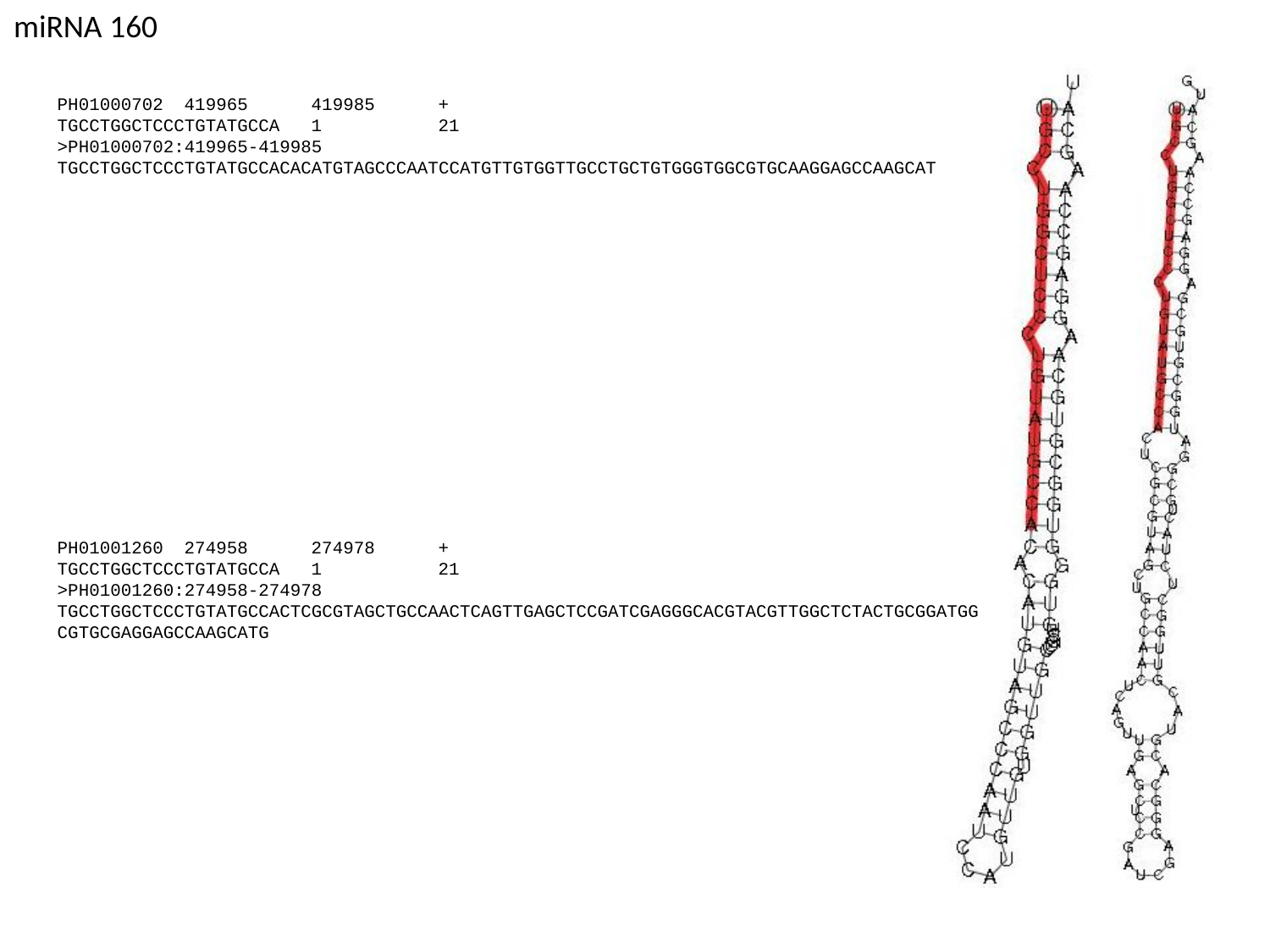

miRNA 160
PH01000702	419965	419985	+
TGCCTGGCTCCCTGTATGCCA 	1	21
>PH01000702:419965-419985
TGCCTGGCTCCCTGTATGCCACACATGTAGCCCAATCCATGTTGTGGTTGCCTGCTGTGGGTGGCGTGCAAGGAGCCAAGCAT
PH01001260	274958	274978	+
TGCCTGGCTCCCTGTATGCCA 	1	21
>PH01001260:274958-274978
TGCCTGGCTCCCTGTATGCCACTCGCGTAGCTGCCAACTCAGTTGAGCTCCGATCGAGGGCACGTACGTTGGCTCTACTGCGGATGGCGTGCGAGGAGCCAAGCATG

## Slide 11
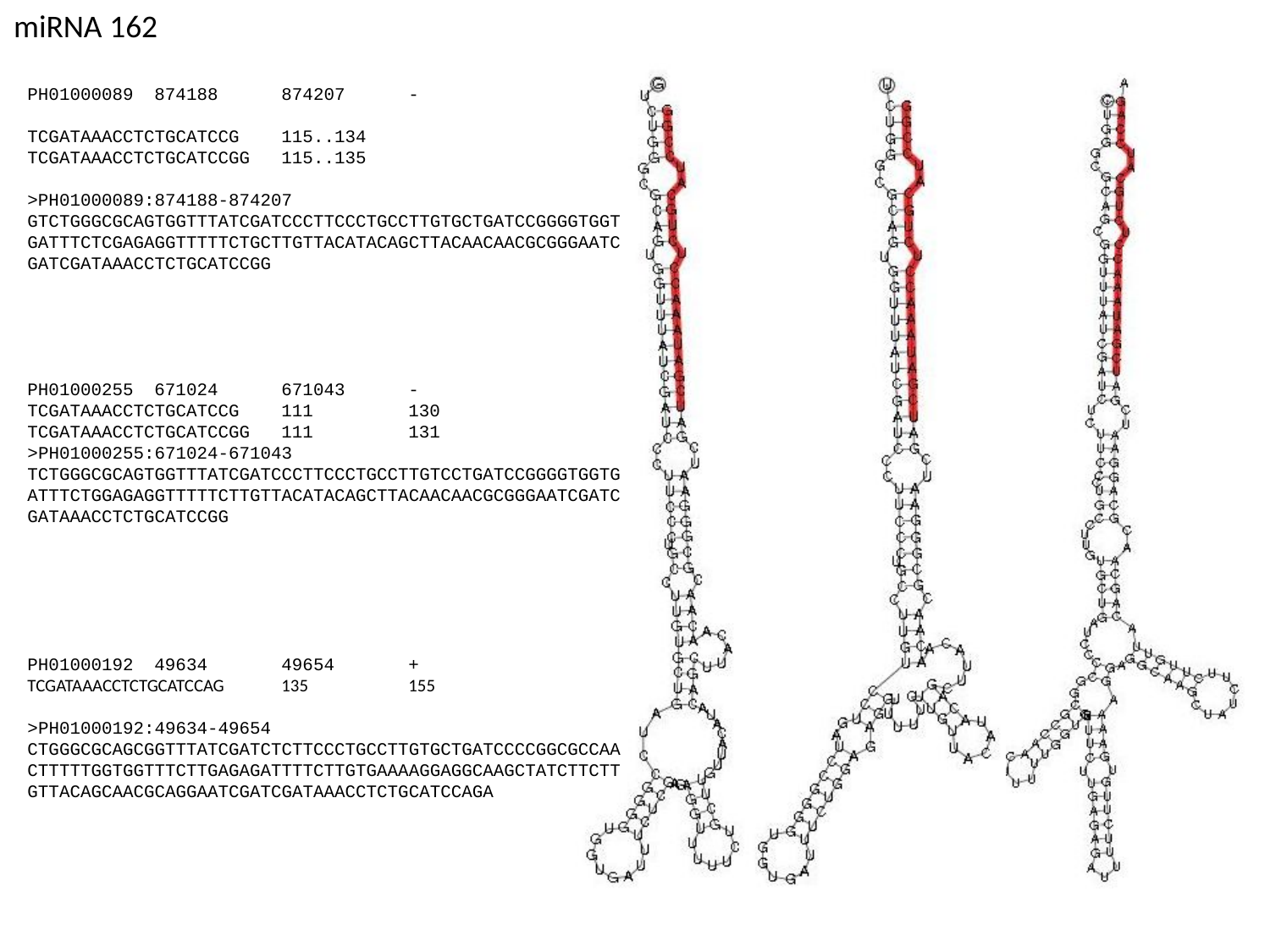

miRNA 162
PH01000089	874188	874207	-
TCGATAAACCTCTGCATCCG 	115..134
TCGATAAACCTCTGCATCCGG 	115..135
>PH01000089:874188-874207
GTCTGGGCGCAGTGGTTTATCGATCCCTTCCCTGCCTTGTGCTGATCCGGGGTGGTGATTTCTCGAGAGGTTTTTCTGCTTGTTACATACAGCTTACAACAACGCGGGAATCGATCGATAAACCTCTGCATCCGG
PH01000255	671024	671043	-
TCGATAAACCTCTGCATCCG 	111	130
TCGATAAACCTCTGCATCCGG 	111	131
>PH01000255:671024-671043
TCTGGGCGCAGTGGTTTATCGATCCCTTCCCTGCCTTGTCCTGATCCGGGGTGGTGATTTCTGGAGAGGTTTTTCTTGTTACATACAGCTTACAACAACGCGGGAATCGATCGATAAACCTCTGCATCCGG
PH01000192	49634	49654	+
TCGATAAACCTCTGCATCCAG 	135	155
>PH01000192:49634-49654
CTGGGCGCAGCGGTTTATCGATCTCTTCCCTGCCTTGTGCTGATCCCCGGCGCCAACTTTTTGGTGGTTTCTTGAGAGATTTTCTTGTGAAAAGGAGGCAAGCTATCTTCTTGTTACAGCAACGCAGGAATCGATCGATAAACCTCTGCATCCAGA

## Slide 12
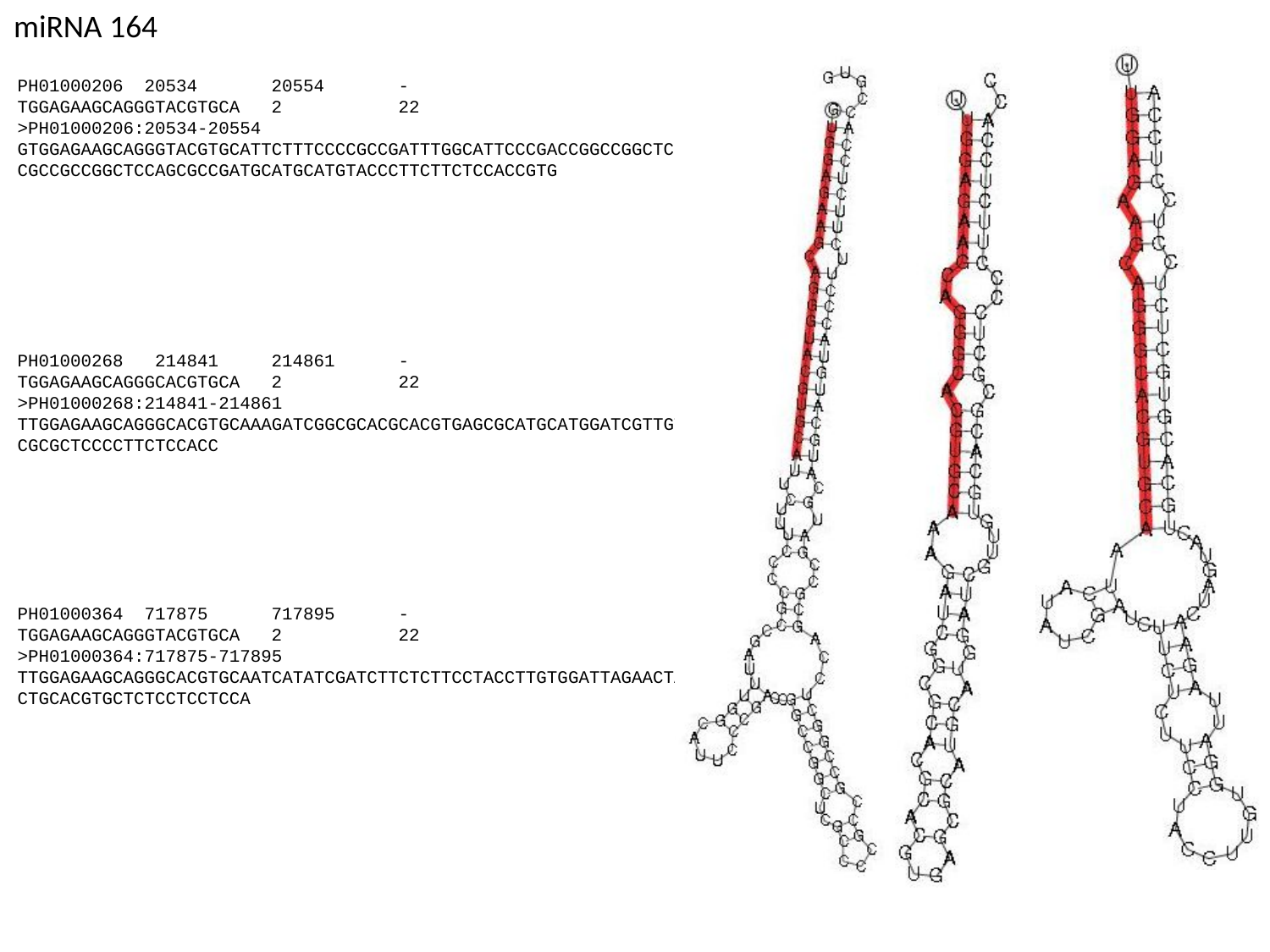

miRNA 164
PH01000206	20534	20554	-
TGGAGAAGCAGGGTACGTGCA 	2	22
>PH01000206:20534-20554
GTGGAGAAGCAGGGTACGTGCATTCTTTCCCCGCCGATTTGGCATTCCCGACCGGCCGGCTCGCCCCGCCGCCGGCTCCAGCGCCGATGCATGCATGTACCCTTCTTCTCCACCGTG
PH01000268	 214841 	214861	-
TGGAGAAGCAGGGCACGTGCA 	2	22
>PH01000268:214841-214861
TTGGAGAAGCAGGGCACGTGCAAAGATCGGCGCACGCACGTGAGCGCATGCATGGATCGTTGTGCACGCGCTCCCCTTCTCCACC
PH01000364	717875	717895	-
TGGAGAAGCAGGGTACGTGCA 	2	22
>PH01000364:717875-717895
TTGGAGAAGCAGGGCACGTGCAATCATATCGATCTTCTCTTCCTACCTTGTGGATTAGAACTAGTACTGCACGTGCTCTCCTCCTCCA

## Slide 13
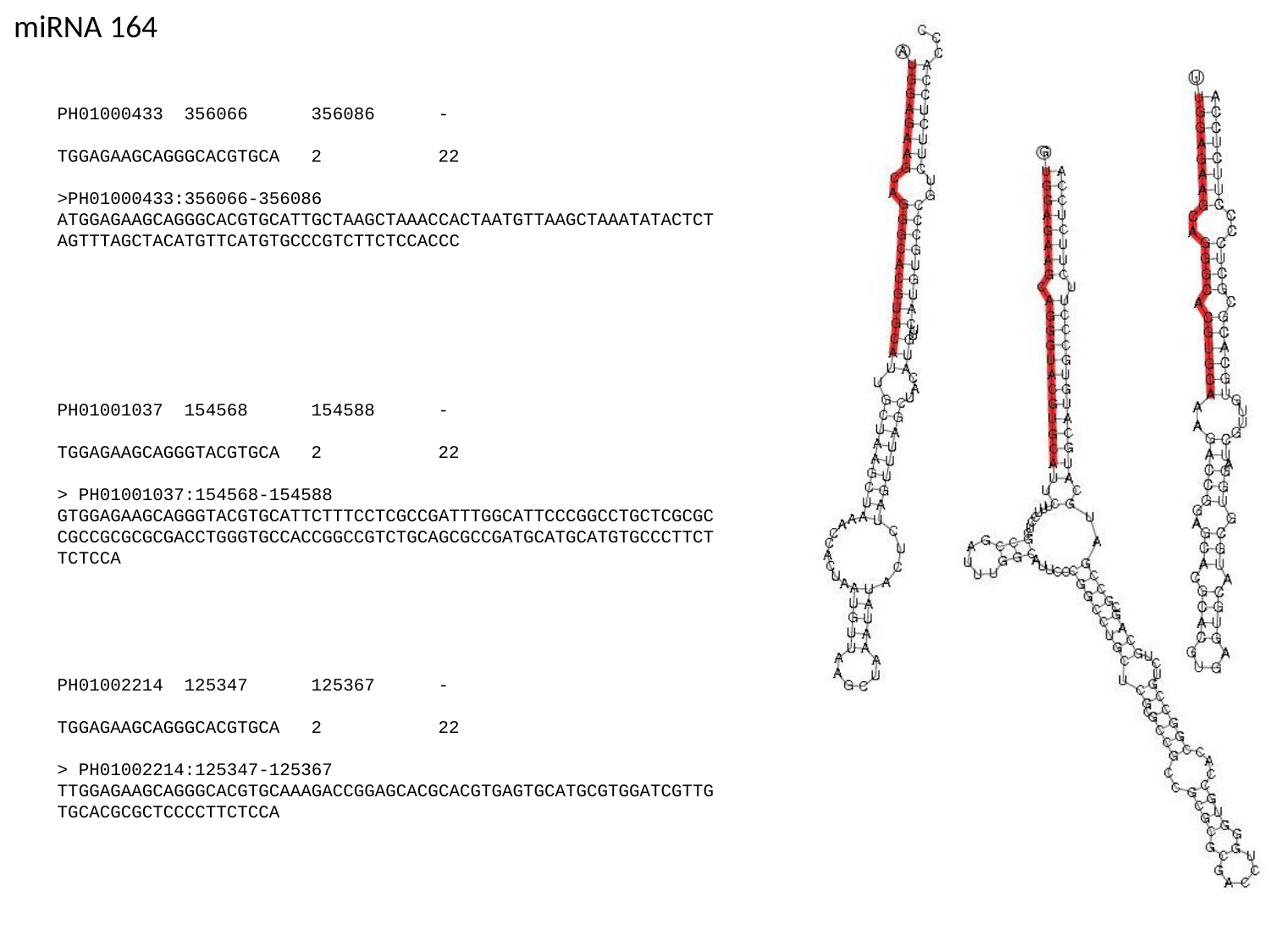

miRNA 164
PH01000433	356066	356086	-
TGGAGAAGCAGGGCACGTGCA 	2	22
>PH01000433:356066-356086
ATGGAGAAGCAGGGCACGTGCATTGCTAAGCTAAACCACTAATGTTAAGCTAAATATACTCTAGTTTAGCTACATGTTCATGTGCCCGTCTTCTCCACCC
PH01001037	154568	154588	-
TGGAGAAGCAGGGTACGTGCA 	2	22
> PH01001037:154568-154588
GTGGAGAAGCAGGGTACGTGCATTCTTTCCTCGCCGATTTGGCATTCCCGGCCTGCTCGCGCCGCCGCGCGCGACCTGGGTGCCACCGGCCGTCTGCAGCGCCGATGCATGCATGTGCCCTTCTTCTCCA
PH01002214	125347	125367	-
TGGAGAAGCAGGGCACGTGCA 	2	22
> PH01002214:125347-125367
TTGGAGAAGCAGGGCACGTGCAAAGACCGGAGCACGCACGTGAGTGCATGCGTGGATCGTTGTGCACGCGCTCCCCTTCTCCA

## Slide 14
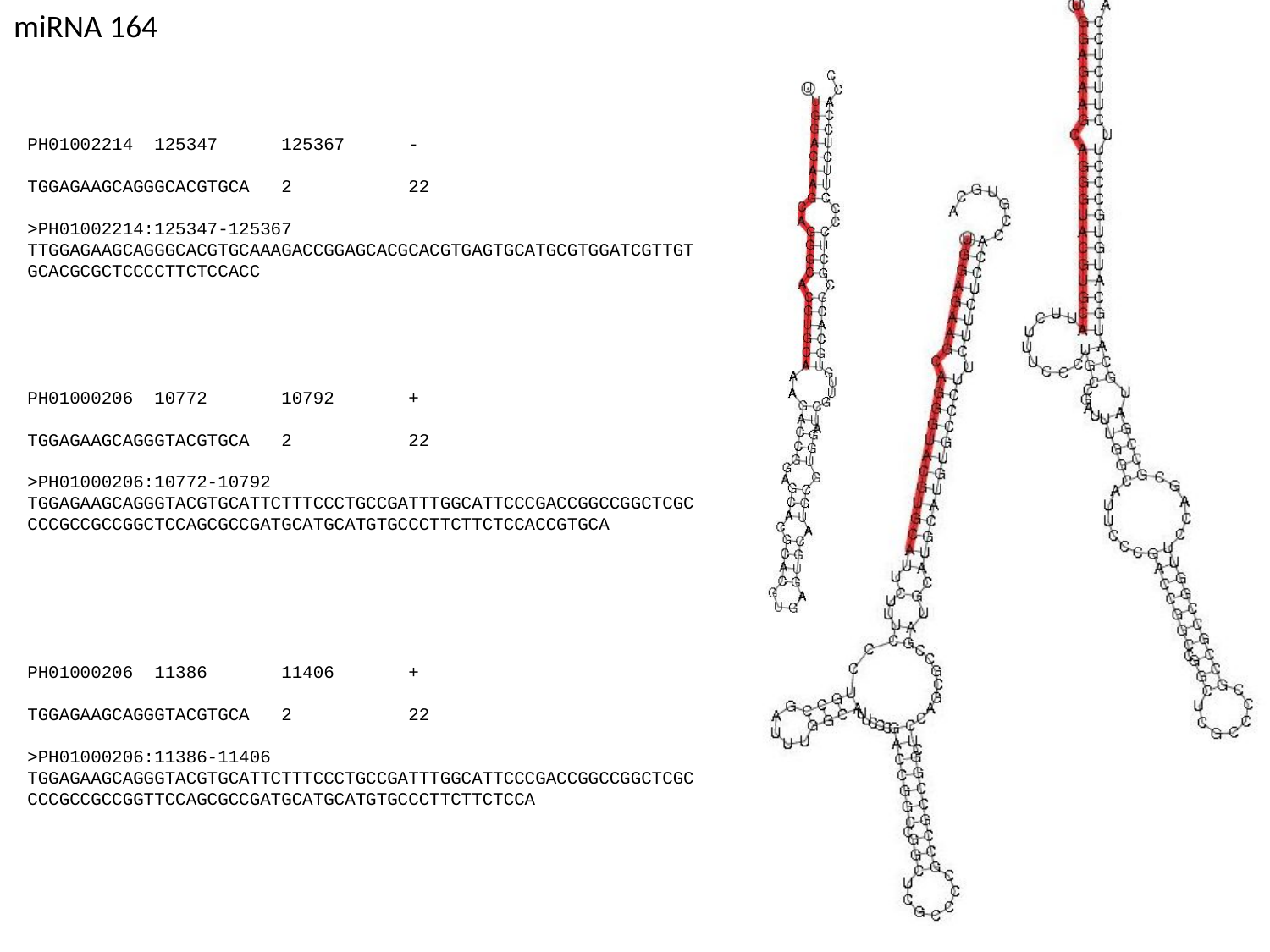

miRNA 164
PH01002214	125347	125367	-
TGGAGAAGCAGGGCACGTGCA 	2	22
>PH01002214:125347-125367
TTGGAGAAGCAGGGCACGTGCAAAGACCGGAGCACGCACGTGAGTGCATGCGTGGATCGTTGTGCACGCGCTCCCCTTCTCCACC
PH01000206	10772	10792	+
TGGAGAAGCAGGGTACGTGCA 	2	22
>PH01000206:10772-10792
TGGAGAAGCAGGGTACGTGCATTCTTTCCCTGCCGATTTGGCATTCCCGACCGGCCGGCTCGCCCCGCCGCCGGCTCCAGCGCCGATGCATGCATGTGCCCTTCTTCTCCACCGTGCA
PH01000206	11386	11406	+
TGGAGAAGCAGGGTACGTGCA 	2	22
>PH01000206:11386-11406
TGGAGAAGCAGGGTACGTGCATTCTTTCCCTGCCGATTTGGCATTCCCGACCGGCCGGCTCGCCCCGCCGCCGGTTCCAGCGCCGATGCATGCATGTGCCCTTCTTCTCCA

## Slide 15
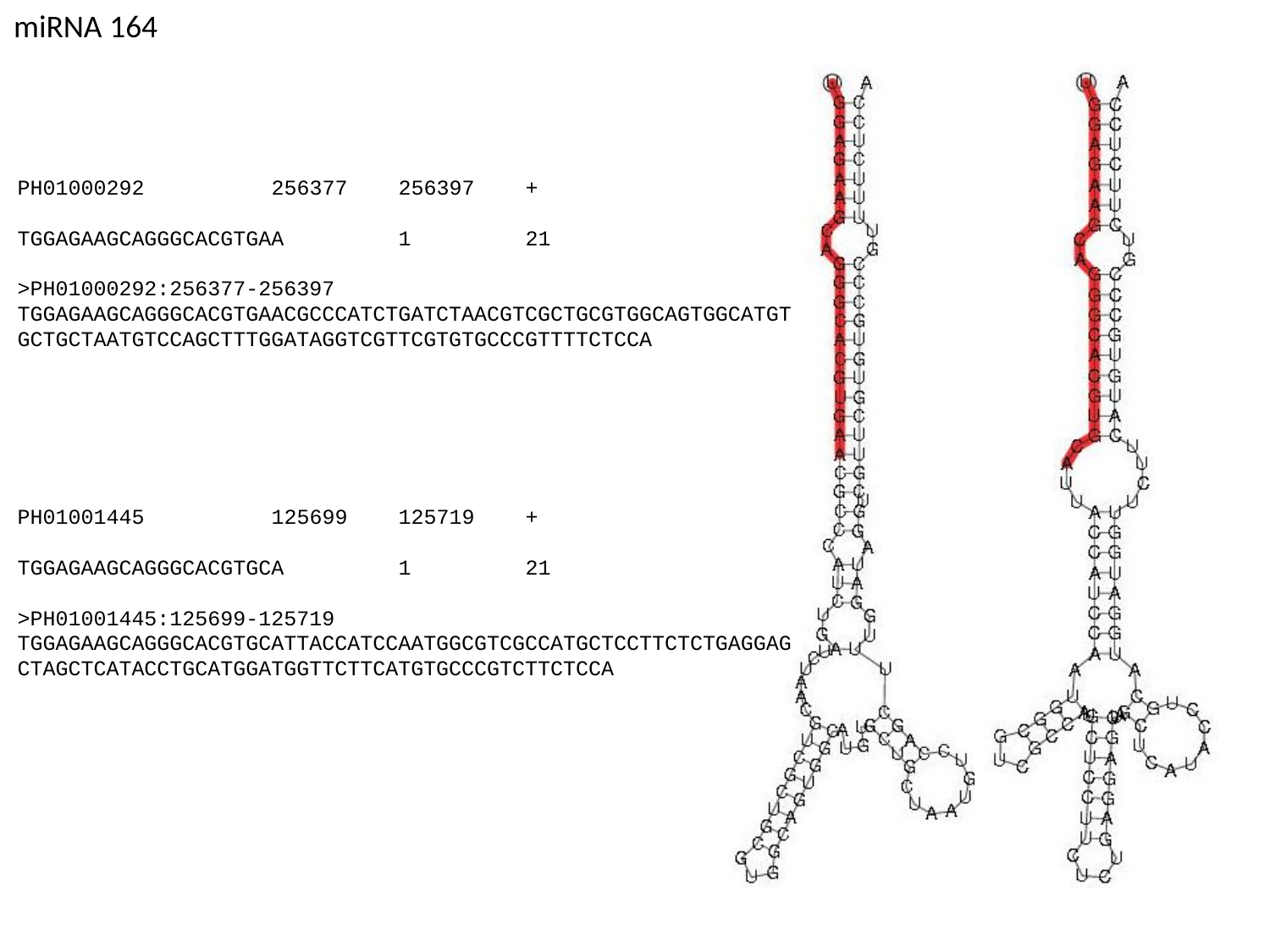

miRNA 164
PH01000292	256377	256397	+
TGGAGAAGCAGGGCACGTGAA 	1	21
>PH01000292:256377-256397
TGGAGAAGCAGGGCACGTGAACGCCCATCTGATCTAACGTCGCTGCGTGGCAGTGGCATGTGCTGCTAATGTCCAGCTTTGGATAGGTCGTTCGTGTGCCCGTTTTCTCCA
PH01001445	125699	125719	+
TGGAGAAGCAGGGCACGTGCA 	1	21
>PH01001445:125699-125719
TGGAGAAGCAGGGCACGTGCATTACCATCCAATGGCGTCGCCATGCTCCTTCTCTGAGGAGCTAGCTCATACCTGCATGGATGGTTCTTCATGTGCCCGTCTTCTCCA

## Slide 16
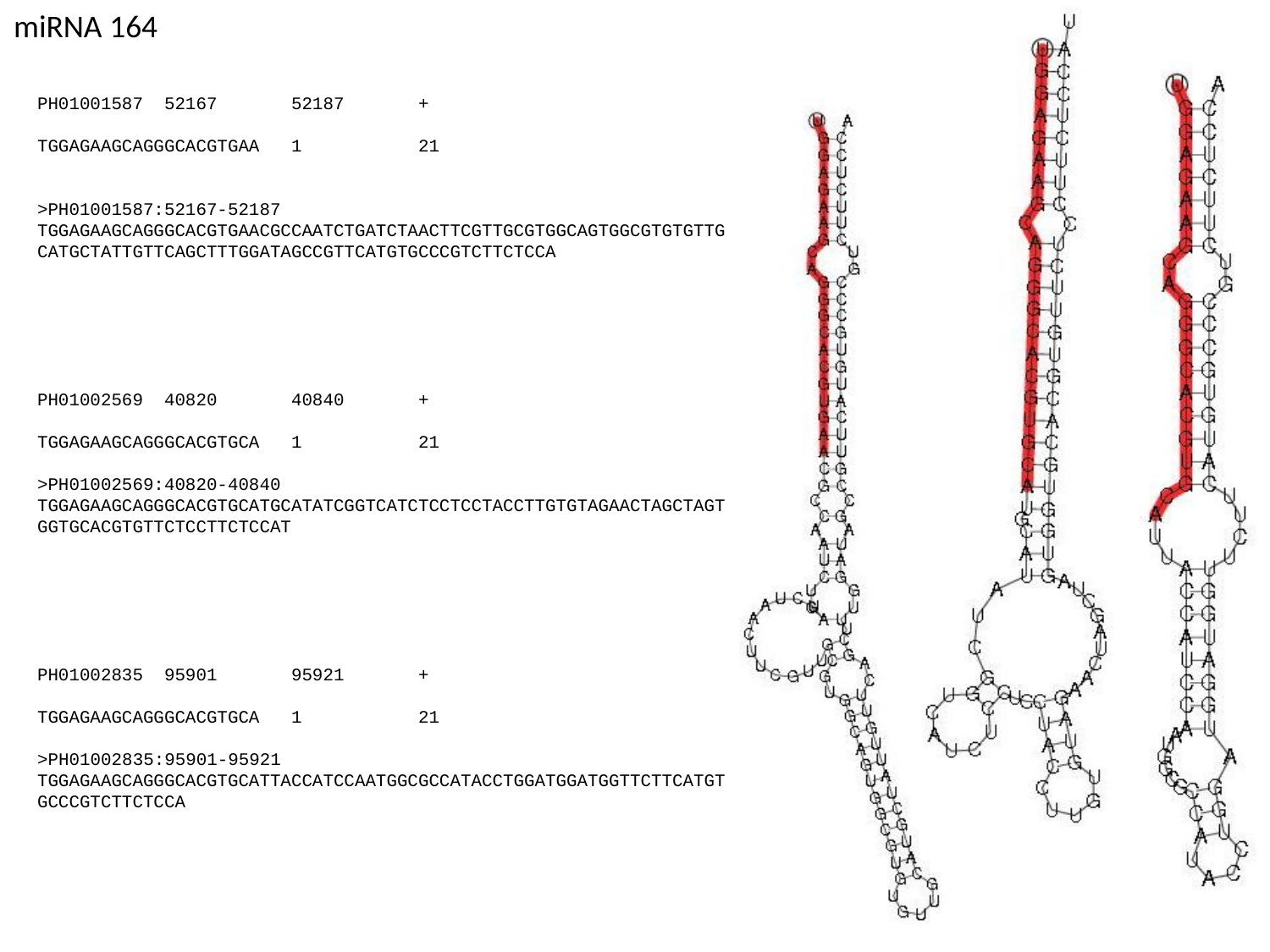

miRNA 164
PH01001587	52167	52187	+
TGGAGAAGCAGGGCACGTGAA 	1	21
>PH01001587:52167-52187
TGGAGAAGCAGGGCACGTGAACGCCAATCTGATCTAACTTCGTTGCGTGGCAGTGGCGTGTGTTGCATGCTATTGTTCAGCTTTGGATAGCCGTTCATGTGCCCGTCTTCTCCA
PH01002569	40820	40840	+
TGGAGAAGCAGGGCACGTGCA 	1	21
>PH01002569:40820-40840
TGGAGAAGCAGGGCACGTGCATGCATATCGGTCATCTCCTCCTACCTTGTGTAGAACTAGCTAGTGGTGCACGTGTTCTCCTTCTCCAT
PH01002835	95901	95921	+
TGGAGAAGCAGGGCACGTGCA 	1	21
>PH01002835:95901-95921
TGGAGAAGCAGGGCACGTGCATTACCATCCAATGGCGCCATACCTGGATGGATGGTTCTTCATGTGCCCGTCTTCTCCA

## Slide 17
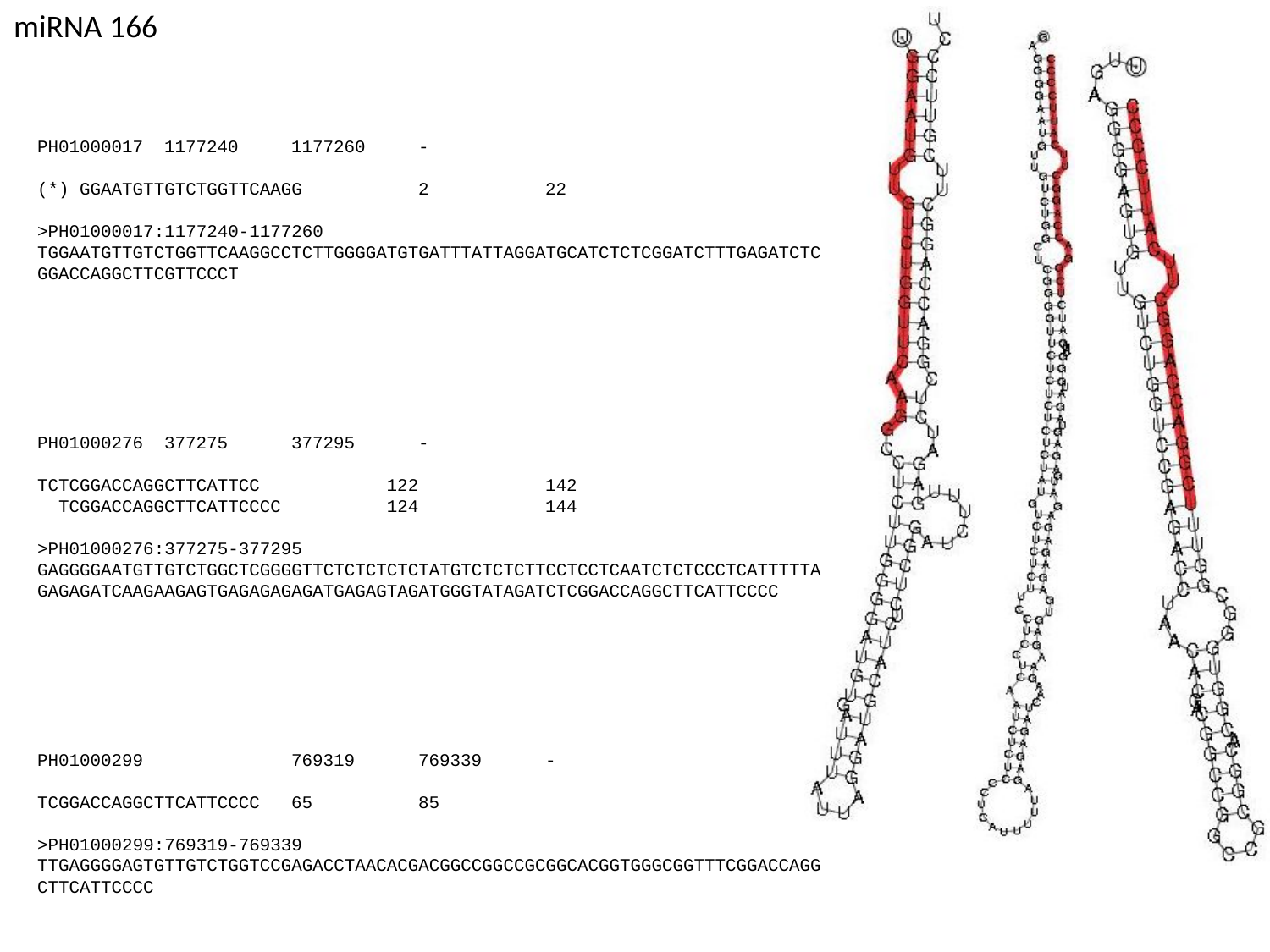

miRNA 166
PH01000017	1177240	1177260	-
(*) GGAATGTTGTCTGGTTCAAGG 	2	22
>PH01000017:1177240-1177260
TGGAATGTTGTCTGGTTCAAGGCCTCTTGGGGATGTGATTTATTAGGATGCATCTCTCGGATCTTTGAGATCTCGGACCAGGCTTCGTTCCCT
PH01000276	377275	377295	-
TCTCGGACCAGGCTTCATTCC 	 122	142
 TCGGACCAGGCTTCATTCCCC 124	144
>PH01000276:377275-377295
GAGGGGAATGTTGTCTGGCTCGGGGTTCTCTCTCTCTATGTCTCTCTTCCTCCTCAATCTCTCCCTCATTTTTAGAGAGATCAAGAAGAGTGAGAGAGAGATGAGAGTAGATGGGTATAGATCTCGGACCAGGCTTCATTCCCC
PH01000299		769319	769339	-
TCGGACCAGGCTTCATTCCCC 	65	85
>PH01000299:769319-769339
TTGAGGGGAGTGTTGTCTGGTCCGAGACCTAACACGACGGCCGGCCGCGGCACGGTGGGCGGTTTCGGACCAGGCTTCATTCCCC

## Slide 18
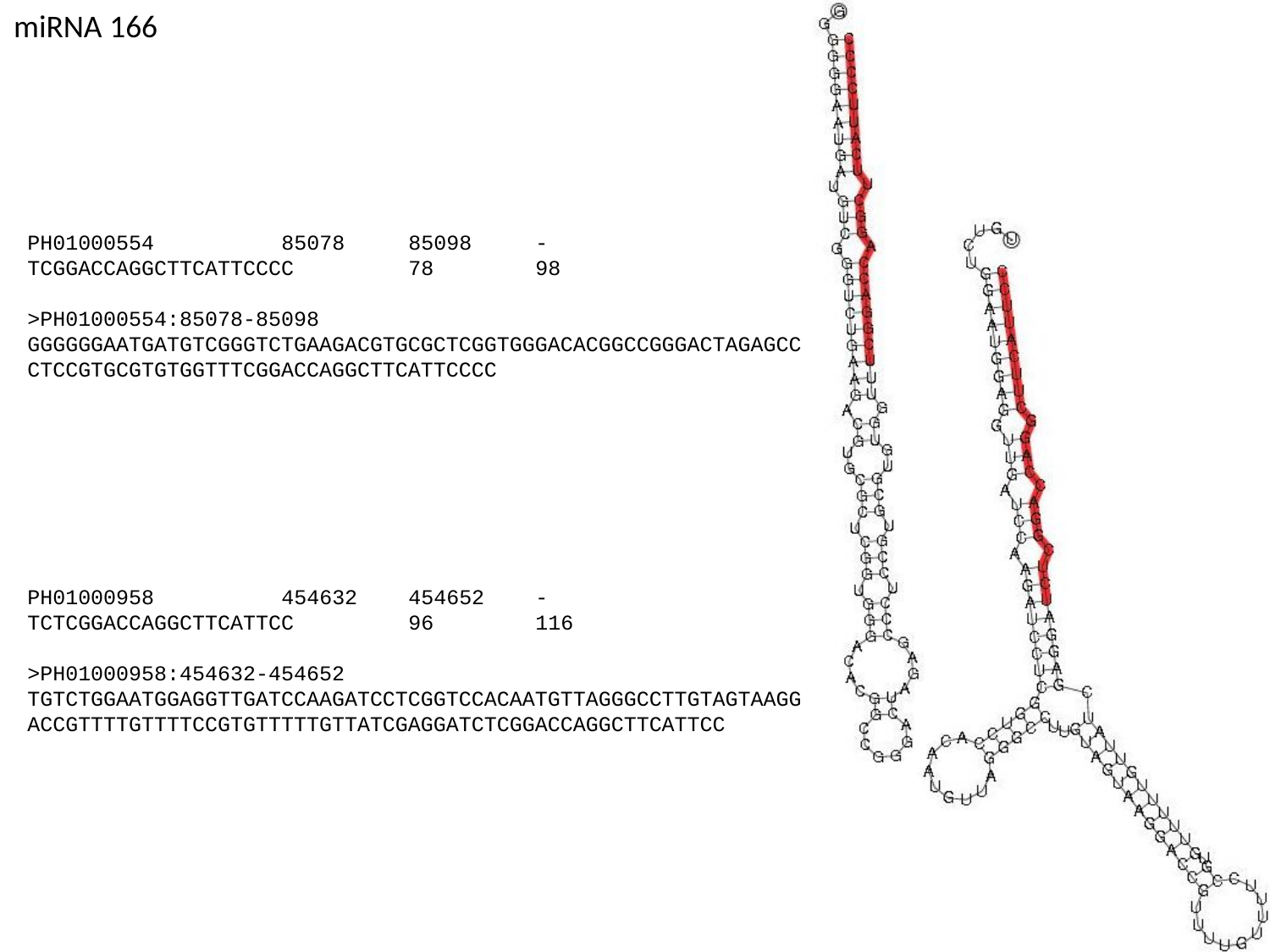

miRNA 166
PH01000554	85078	85098	-
TCGGACCAGGCTTCATTCCCC 	78	98
>PH01000554:85078-85098
GGGGGGAATGATGTCGGGTCTGAAGACGTGCGCTCGGTGGGACACGGCCGGGACTAGAGCCCTCCGTGCGTGTGGTTTCGGACCAGGCTTCATTCCCC
PH01000958	454632	454652	-
TCTCGGACCAGGCTTCATTCC 	96	116
>PH01000958:454632-454652
TGTCTGGAATGGAGGTTGATCCAAGATCCTCGGTCCACAATGTTAGGGCCTTGTAGTAAGGACCGTTTTGTTTTCCGTGTTTTTGTTATCGAGGATCTCGGACCAGGCTTCATTCC

## Slide 19
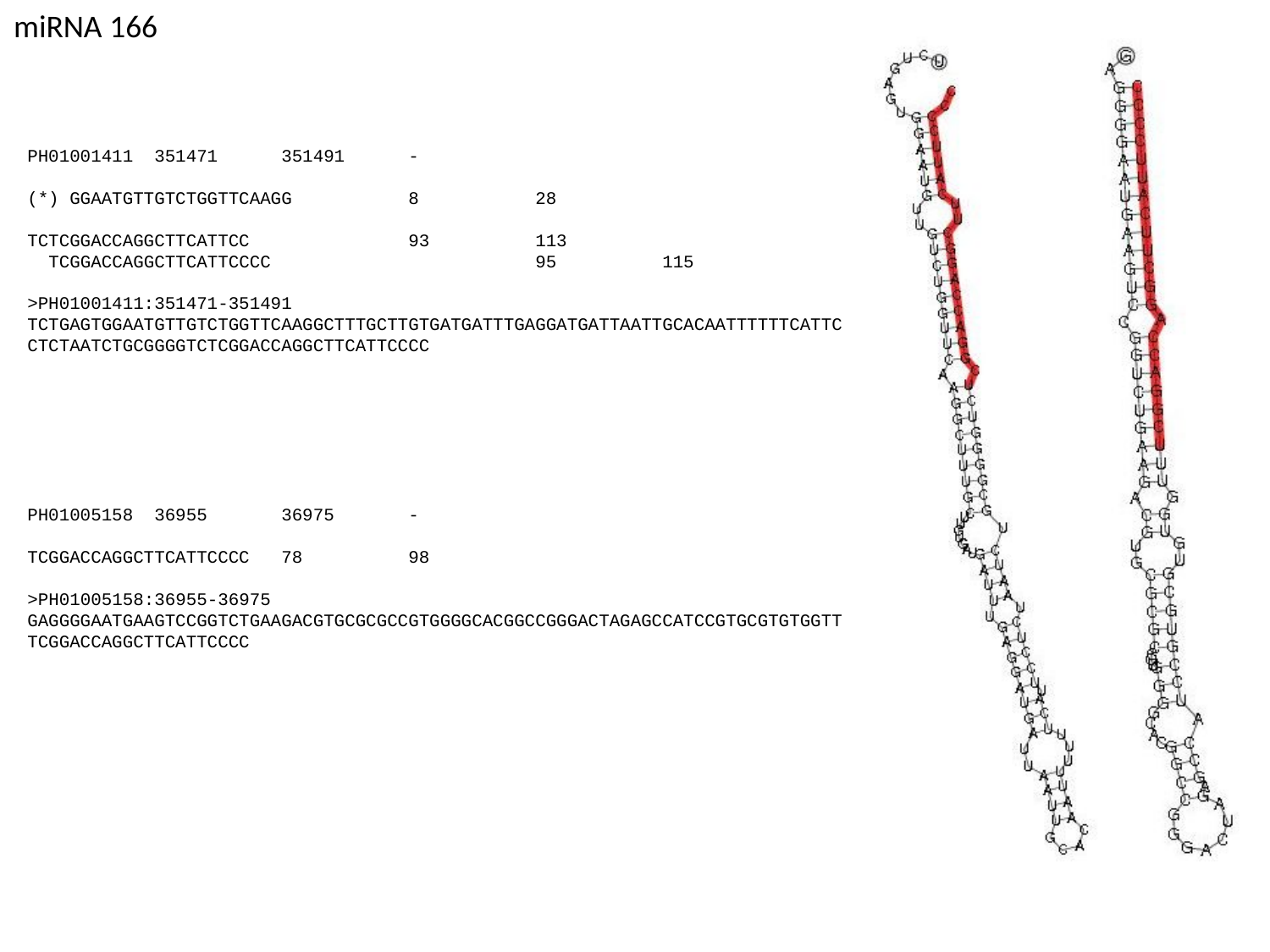

miRNA 166
PH01001411	351471	351491	-
(*) GGAATGTTGTCTGGTTCAAGG 	8	28
TCTCGGACCAGGCTTCATTCC 	 93	113
 TCGGACCAGGCTTCATTCCCC 	 95	115
>PH01001411:351471-351491
TCTGAGTGGAATGTTGTCTGGTTCAAGGCTTTGCTTGTGATGATTTGAGGATGATTAATTGCACAATTTTTTCATTCCTCTAATCTGCGGGGTCTCGGACCAGGCTTCATTCCCC
PH01005158	36955	36975	-
TCGGACCAGGCTTCATTCCCC 	78	98
>PH01005158:36955-36975 GAGGGGAATGAAGTCCGGTCTGAAGACGTGCGCGCCGTGGGGCACGGCCGGGACTAGAGCCATCCGTGCGTGTGGTTTCGGACCAGGCTTCATTCCCC

## Slide 20
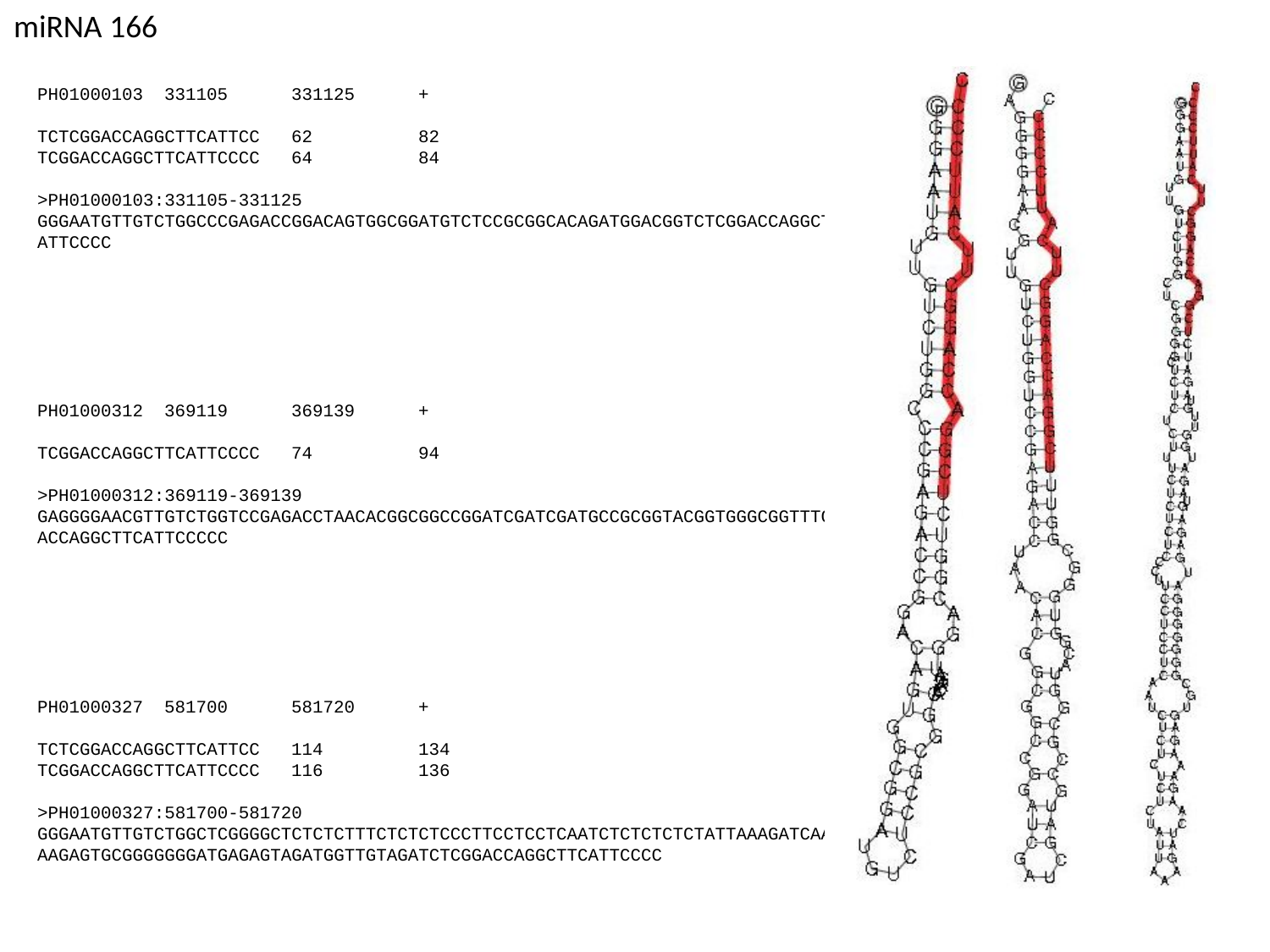

miRNA 166
PH01000103	331105	331125	+
TCTCGGACCAGGCTTCATTCC 	62	82
TCGGACCAGGCTTCATTCCCC 	64	84
>PH01000103:331105-331125
GGGAATGTTGTCTGGCCCGAGACCGGACAGTGGCGGATGTCTCCGCGGCACAGATGGACGGTCTCGGACCAGGCTTCATTCCCC
PH01000312	369119	369139	+
TCGGACCAGGCTTCATTCCCC 	74	94
>PH01000312:369119-369139
GAGGGGAACGTTGTCTGGTCCGAGACCTAACACGGCGGCCGGATCGATCGATGCCGCGGTACGGTGGGCGGTTTCGGACCAGGCTTCATTCCCCC
PH01000327	581700	581720	+
TCTCGGACCAGGCTTCATTCC 	114	134
TCGGACCAGGCTTCATTCCCC 	116	136
>PH01000327:581700-581720
GGGAATGTTGTCTGGCTCGGGGCTCTCTCTTTCTCTCTCCCTTCCTCCTCAATCTCTCTCTCTATTAAAGATCAAGAAAGAGTGCGGGGGGGATGAGAGTAGATGGTTGTAGATCTCGGACCAGGCTTCATTCCCC

## Slide 21
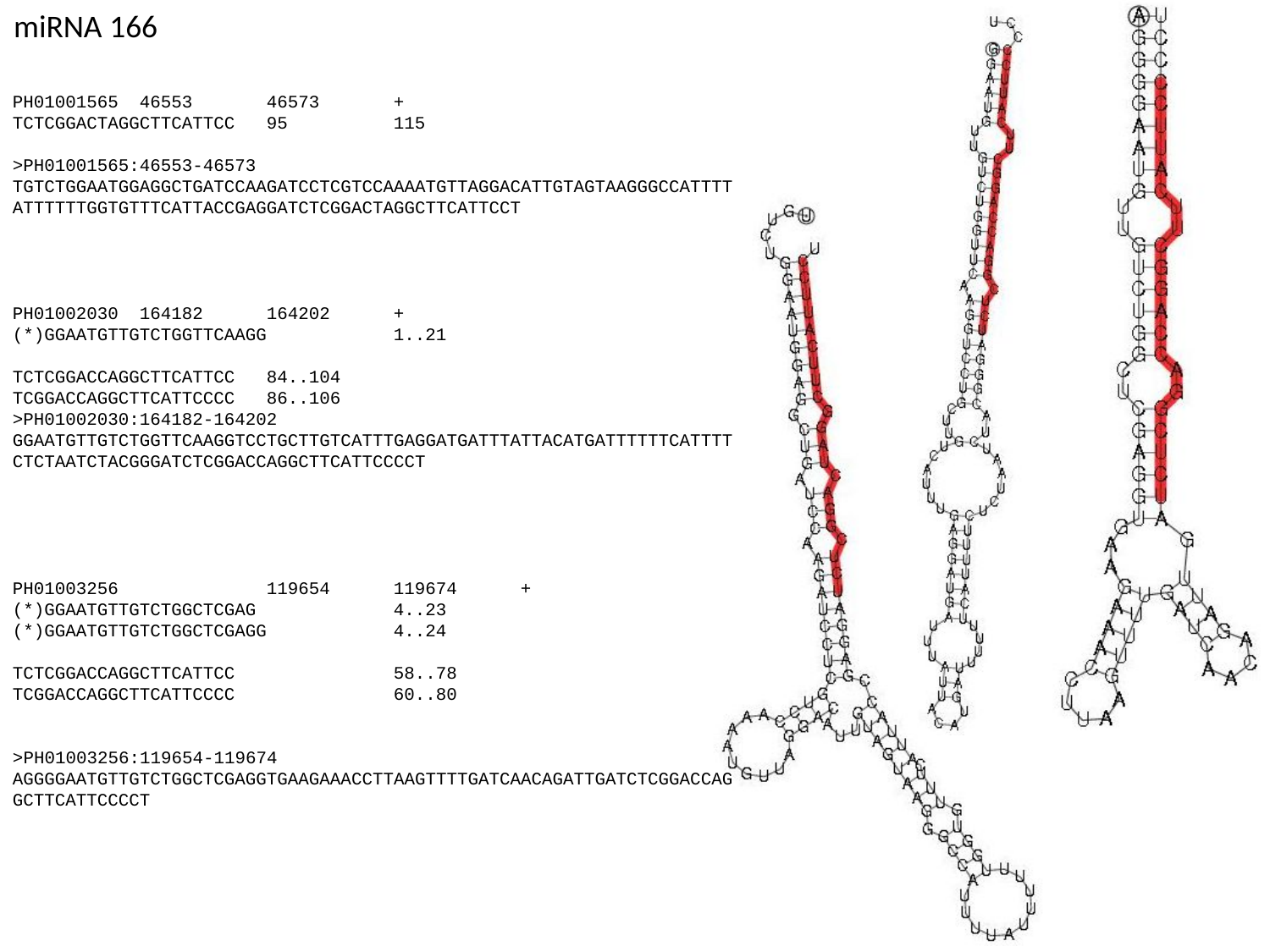

miRNA 166
PH01001565	46553	46573	+
TCTCGGACTAGGCTTCATTCC 	95	115
>PH01001565:46553-46573
TGTCTGGAATGGAGGCTGATCCAAGATCCTCGTCCAAAATGTTAGGACATTGTAGTAAGGGCCATTTTATTTTTTGGTGTTTCATTACCGAGGATCTCGGACTAGGCTTCATTCCT
PH01002030	164182	164202	+
(*)GGAATGTTGTCTGGTTCAAGG 	1..21
TCTCGGACCAGGCTTCATTCC 	84..104
TCGGACCAGGCTTCATTCCCC 	86..106
>PH01002030:164182-164202
GGAATGTTGTCTGGTTCAAGGTCCTGCTTGTCATTTGAGGATGATTTATTACATGATTTTTTCATTTTCTCTAATCTACGGGATCTCGGACCAGGCTTCATTCCCCT
PH01003256		119654	119674	+
(*)GGAATGTTGTCTGGCTCGAG 	4..23
(*)GGAATGTTGTCTGGCTCGAGG	4..24
TCTCGGACCAGGCTTCATTCC 		58..78
TCGGACCAGGCTTCATTCCCC 		60..80
>PH01003256:119654-119674
AGGGGAATGTTGTCTGGCTCGAGGTGAAGAAACCTTAAGTTTTGATCAACAGATTGATCTCGGACCAGGCTTCATTCCCCT

## Slide 22
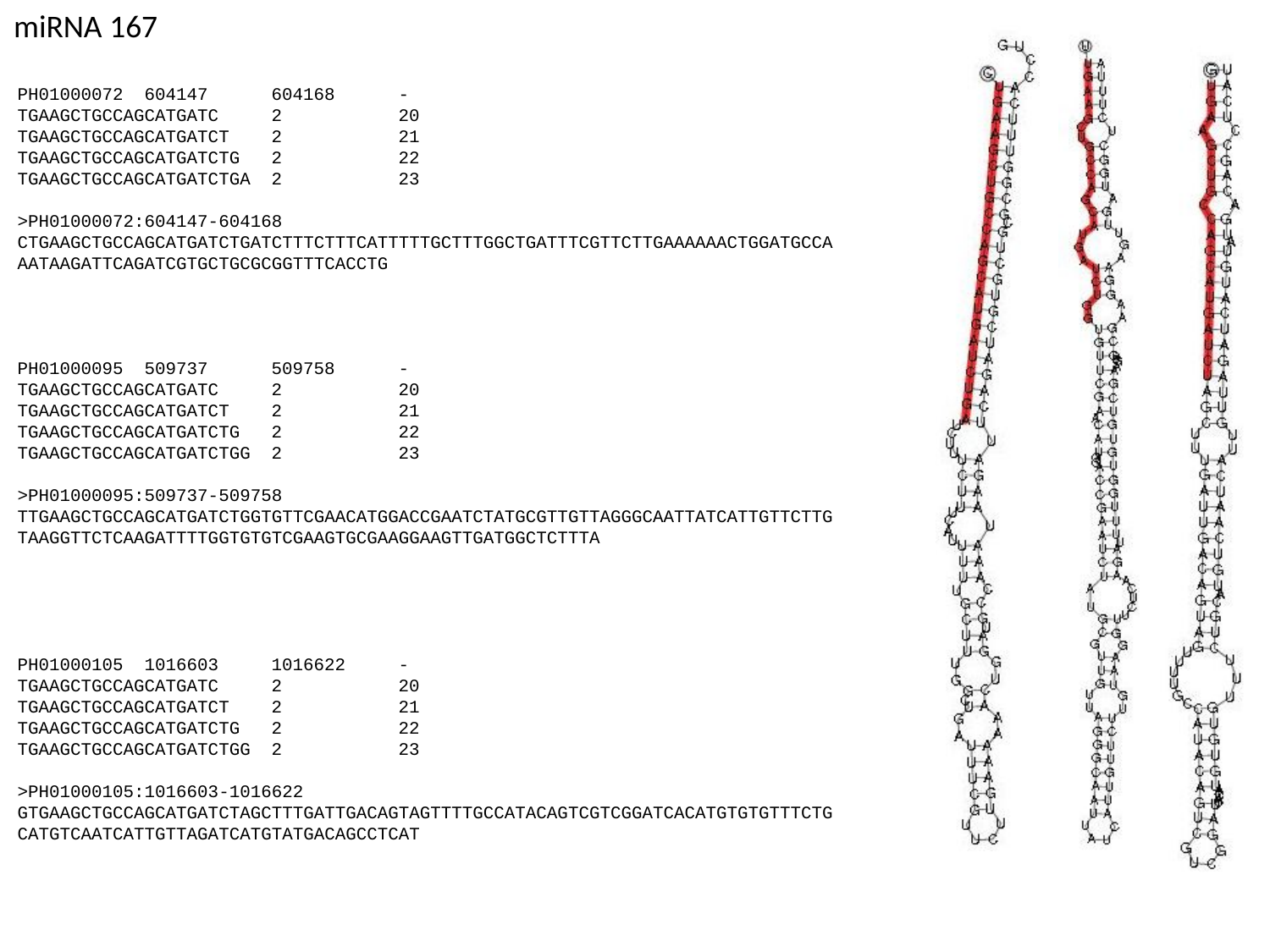

miRNA 167
PH01000072	604147	604168	-
TGAAGCTGCCAGCATGATC 	2	20
TGAAGCTGCCAGCATGATCT 	2	21
TGAAGCTGCCAGCATGATCTG 	2	22
TGAAGCTGCCAGCATGATCTGA 	2	23
>PH01000072:604147-604168
CTGAAGCTGCCAGCATGATCTGATCTTTCTTTCATTTTTGCTTTGGCTGATTTCGTTCTTGAAAAAACTGGATGCCAAATAAGATTCAGATCGTGCTGCGCGGTTTCACCTG
PH01000095	509737	509758	-
TGAAGCTGCCAGCATGATC 	2	20
TGAAGCTGCCAGCATGATCT 	2	21
TGAAGCTGCCAGCATGATCTG 	2	22
TGAAGCTGCCAGCATGATCTGG 	2	23
>PH01000095:509737-509758
TTGAAGCTGCCAGCATGATCTGGTGTTCGAACATGGACCGAATCTATGCGTTGTTAGGGCAATTATCATTGTTCTTGTAAGGTTCTCAAGATTTTGGTGTGTCGAAGTGCGAAGGAAGTTGATGGCTCTTTA
PH01000105	1016603	1016622	-
TGAAGCTGCCAGCATGATC 	2	20
TGAAGCTGCCAGCATGATCT 	2	21
TGAAGCTGCCAGCATGATCTG 	2	22
TGAAGCTGCCAGCATGATCTGG 	2	23
>PH01000105:1016603-1016622
GTGAAGCTGCCAGCATGATCTAGCTTTGATTGACAGTAGTTTTGCCATACAGTCGTCGGATCACATGTGTGTTTCTGCATGTCAATCATTGTTAGATCATGTATGACAGCCTCAT

## Slide 23
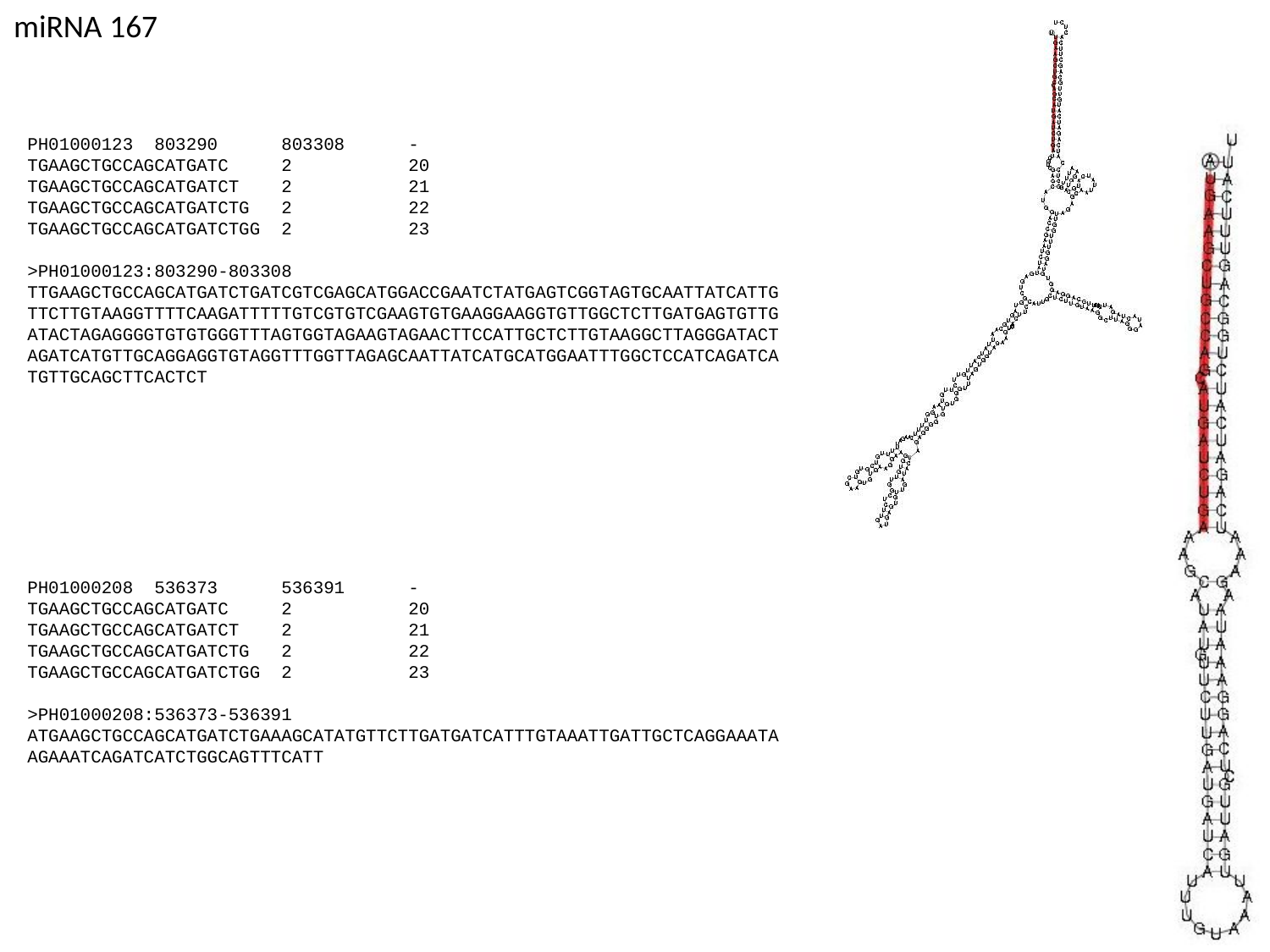

miRNA 167
PH01000123	803290	803308	-
TGAAGCTGCCAGCATGATC 	2	20
TGAAGCTGCCAGCATGATCT 	2	21
TGAAGCTGCCAGCATGATCTG 	2	22
TGAAGCTGCCAGCATGATCTGG 	2	23
>PH01000123:803290-803308
TTGAAGCTGCCAGCATGATCTGATCGTCGAGCATGGACCGAATCTATGAGTCGGTAGTGCAATTATCATTGTTCTTGTAAGGTTTTCAAGATTTTTGTCGTGTCGAAGTGTGAAGGAAGGTGTTGGCTCTTGATGAGTGTTGATACTAGAGGGGTGTGTGGGTTTAGTGGTAGAAGTAGAACTTCCATTGCTCTTGTAAGGCTTAGGGATACTAGATCATGTTGCAGGAGGTGTAGGTTTGGTTAGAGCAATTATCATGCATGGAATTTGGCTCCATCAGATCATGTTGCAGCTTCACTCT
PH01000208	536373	536391	-
TGAAGCTGCCAGCATGATC 	2	20
TGAAGCTGCCAGCATGATCT 	2	21
TGAAGCTGCCAGCATGATCTG 	2	22
TGAAGCTGCCAGCATGATCTGG 	2	23
>PH01000208:536373-536391
ATGAAGCTGCCAGCATGATCTGAAAGCATATGTTCTTGATGATCATTTGTAAATTGATTGCTCAGGAAATAAGAAATCAGATCATCTGGCAGTTTCATT

## Slide 24
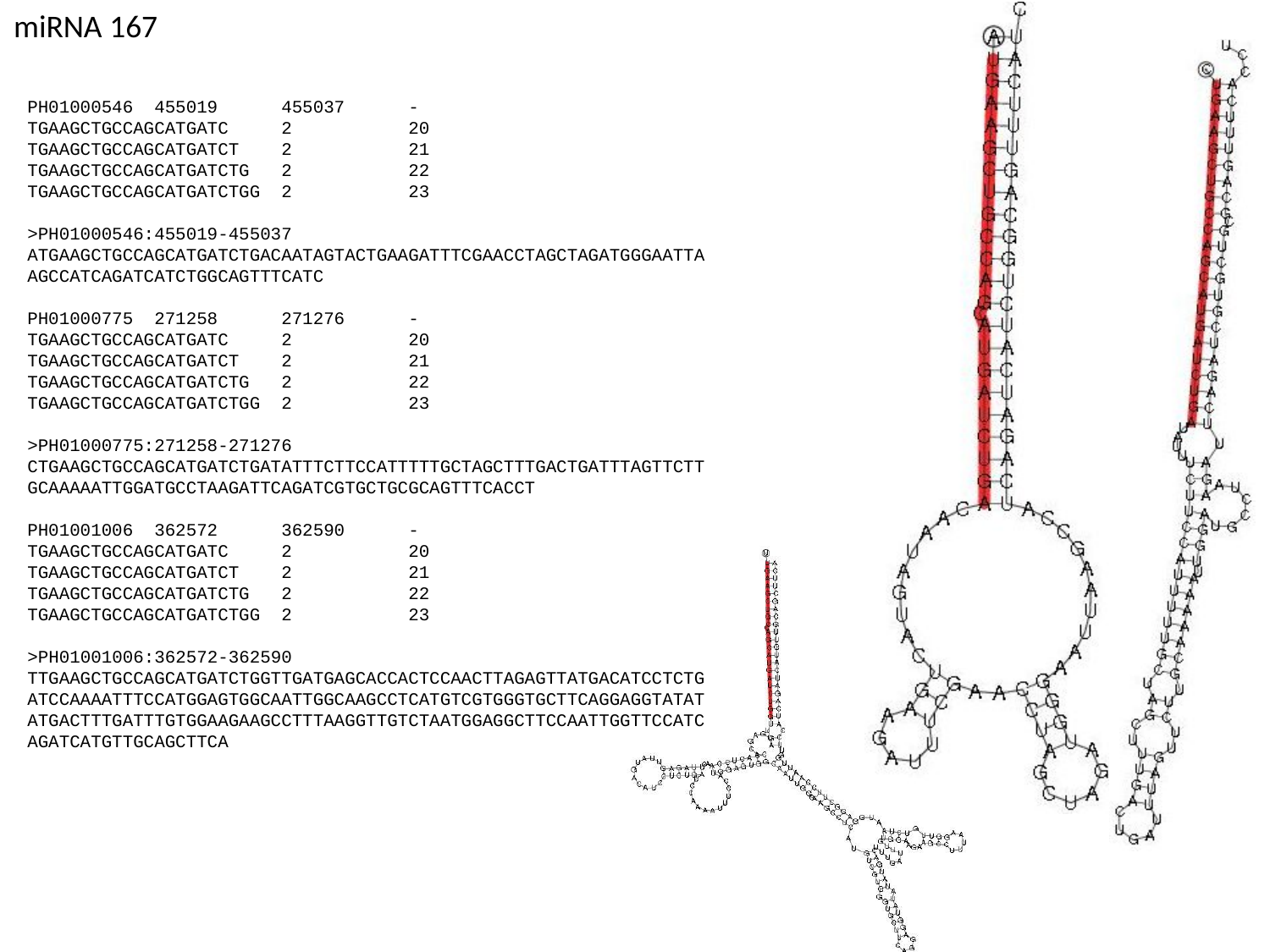

miRNA 167
PH01000546	455019	455037	-
TGAAGCTGCCAGCATGATC 	2	20
TGAAGCTGCCAGCATGATCT 	2	21
TGAAGCTGCCAGCATGATCTG 	2	22
TGAAGCTGCCAGCATGATCTGG 	2	23
>PH01000546:455019-455037
ATGAAGCTGCCAGCATGATCTGACAATAGTACTGAAGATTTCGAACCTAGCTAGATGGGAATTAAGCCATCAGATCATCTGGCAGTTTCATC
PH01000775	271258	271276	-
TGAAGCTGCCAGCATGATC 	2	20
TGAAGCTGCCAGCATGATCT 	2	21
TGAAGCTGCCAGCATGATCTG 	2	22
TGAAGCTGCCAGCATGATCTGG 	2	23
>PH01000775:271258-271276
CTGAAGCTGCCAGCATGATCTGATATTTCTTCCATTTTTGCTAGCTTTGACTGATTTAGTTCTTGCAAAAATTGGATGCCTAAGATTCAGATCGTGCTGCGCAGTTTCACCT
PH01001006	362572	362590	-
TGAAGCTGCCAGCATGATC 	2	20
TGAAGCTGCCAGCATGATCT 	2	21
TGAAGCTGCCAGCATGATCTG 	2	22
TGAAGCTGCCAGCATGATCTGG 	2	23
>PH01001006:362572-362590
TTGAAGCTGCCAGCATGATCTGGTTGATGAGCACCACTCCAACTTAGAGTTATGACATCCTCTGATCCAAAATTTCCATGGAGTGGCAATTGGCAAGCCTCATGTCGTGGGTGCTTCAGGAGGTATATATGACTTTGATTTGTGGAAGAAGCCTTTAAGGTTGTCTAATGGAGGCTTCCAATTGGTTCCATCAGATCATGTTGCAGCTTCA

## Slide 25
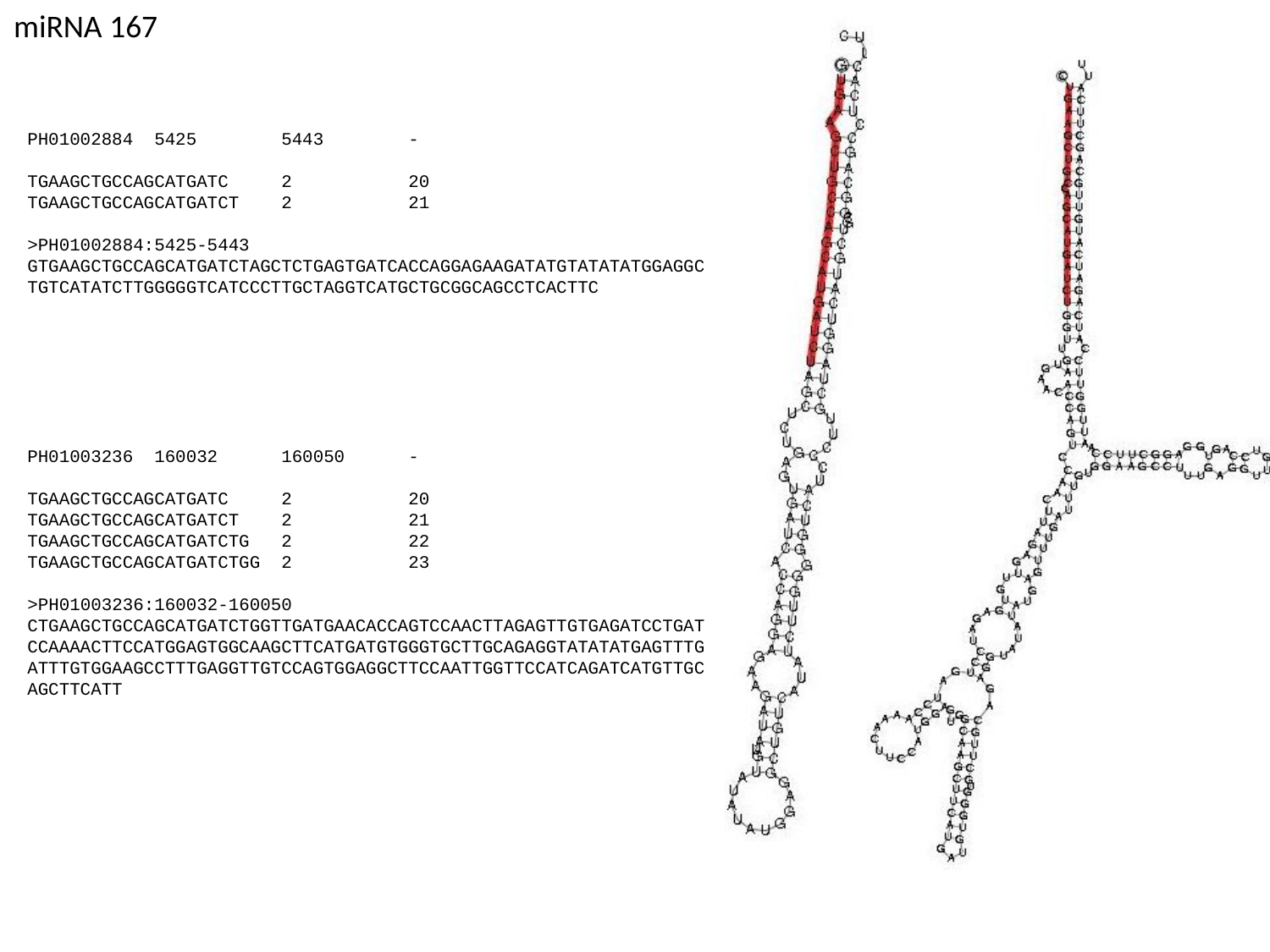

miRNA 167
PH01002884	5425	5443	-
TGAAGCTGCCAGCATGATC 	2	20
TGAAGCTGCCAGCATGATCT 	2	21
>PH01002884:5425-5443
GTGAAGCTGCCAGCATGATCTAGCTCTGAGTGATCACCAGGAGAAGATATGTATATATGGAGGCTGTCATATCTTGGGGGTCATCCCTTGCTAGGTCATGCTGCGGCAGCCTCACTTC
PH01003236	160032	160050	-
TGAAGCTGCCAGCATGATC 	2	20
TGAAGCTGCCAGCATGATCT 	2	21
TGAAGCTGCCAGCATGATCTG 	2	22
TGAAGCTGCCAGCATGATCTGG 	2	23
>PH01003236:160032-160050
CTGAAGCTGCCAGCATGATCTGGTTGATGAACACCAGTCCAACTTAGAGTTGTGAGATCCTGATCCAAAACTTCCATGGAGTGGCAAGCTTCATGATGTGGGTGCTTGCAGAGGTATATATGAGTTTGATTTGTGGAAGCCTTTGAGGTTGTCCAGTGGAGGCTTCCAATTGGTTCCATCAGATCATGTTGCAGCTTCATT

## Slide 26
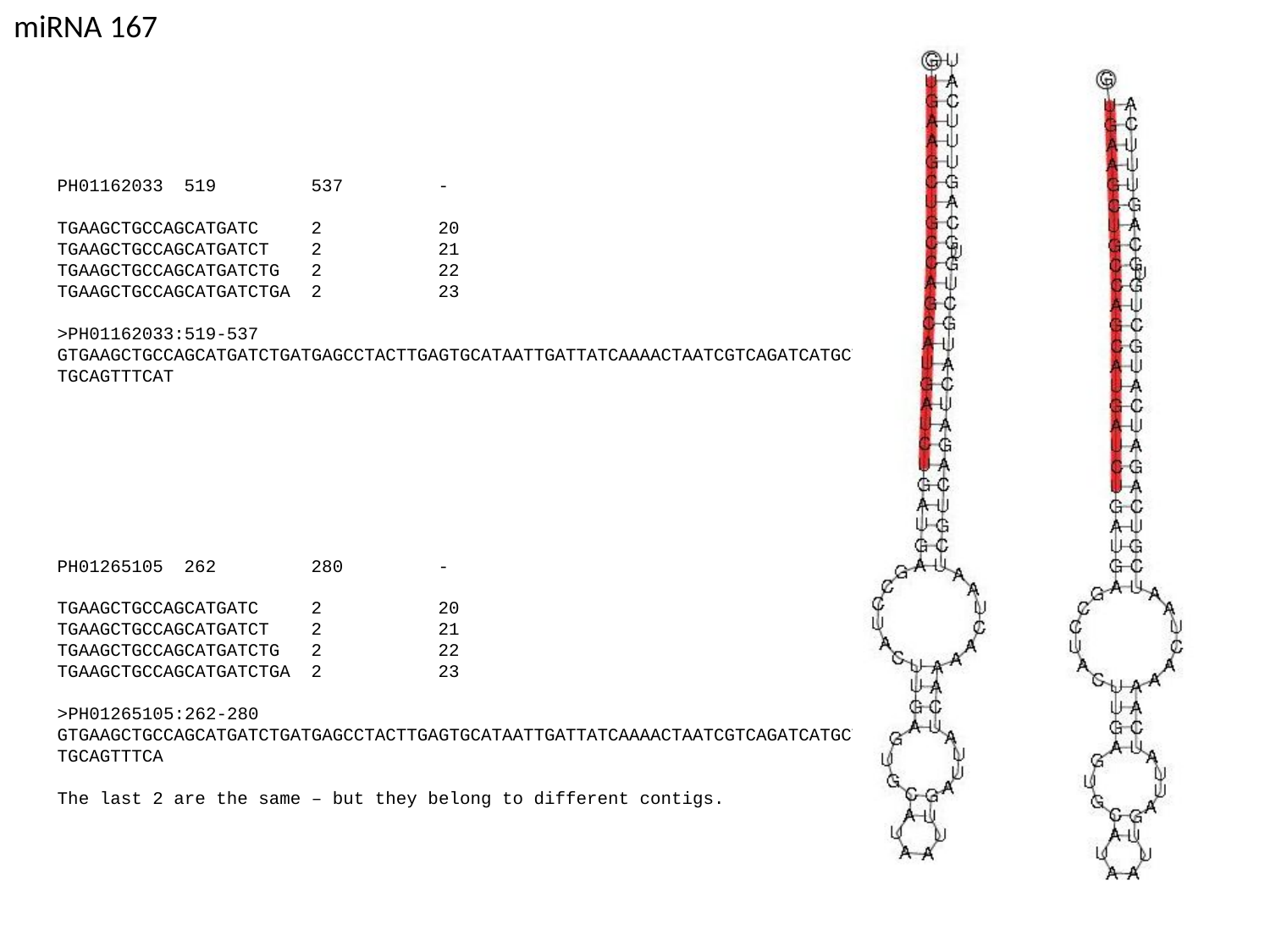

miRNA 167
PH01162033	519	537	-
TGAAGCTGCCAGCATGATC 	2	20
TGAAGCTGCCAGCATGATCT 	2	21
TGAAGCTGCCAGCATGATCTG 	2	22
TGAAGCTGCCAGCATGATCTGA 	2	23
>PH01162033:519-537
GTGAAGCTGCCAGCATGATCTGATGAGCCTACTTGAGTGCATAATTGATTATCAAAACTAATCGTCAGATCATGCTGTGCAGTTTCAT
PH01265105	262	280	-
TGAAGCTGCCAGCATGATC 	2	20
TGAAGCTGCCAGCATGATCT 	2	21
TGAAGCTGCCAGCATGATCTG 	2	22
TGAAGCTGCCAGCATGATCTGA 	2	23
>PH01265105:262-280 GTGAAGCTGCCAGCATGATCTGATGAGCCTACTTGAGTGCATAATTGATTATCAAAACTAATCGTCAGATCATGCTGTGCAGTTTCA
The last 2 are the same – but they belong to different contigs.

## Slide 27
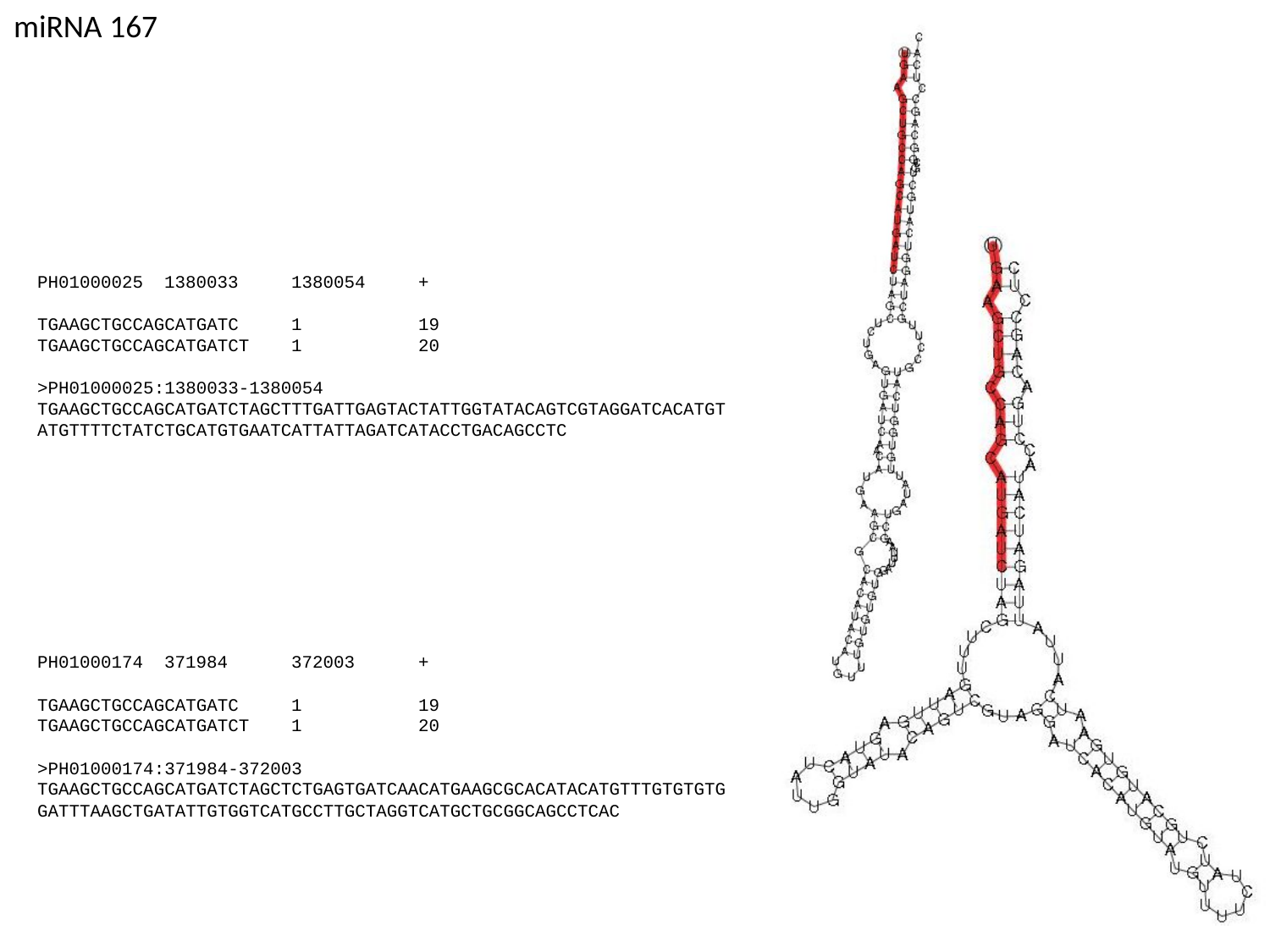

miRNA 167
PH01000025	1380033	1380054	+
TGAAGCTGCCAGCATGATC 	1	19
TGAAGCTGCCAGCATGATCT 	1	20
>PH01000025:1380033-1380054
TGAAGCTGCCAGCATGATCTAGCTTTGATTGAGTACTATTGGTATACAGTCGTAGGATCACATGTATGTTTTCTATCTGCATGTGAATCATTATTAGATCATACCTGACAGCCTC
PH01000174	371984	372003	+
TGAAGCTGCCAGCATGATC 	1	19
TGAAGCTGCCAGCATGATCT 	1	20
>PH01000174:371984-372003
TGAAGCTGCCAGCATGATCTAGCTCTGAGTGATCAACATGAAGCGCACATACATGTTTGTGTGTGGATTTAAGCTGATATTGTGGTCATGCCTTGCTAGGTCATGCTGCGGCAGCCTCAC

## Slide 28
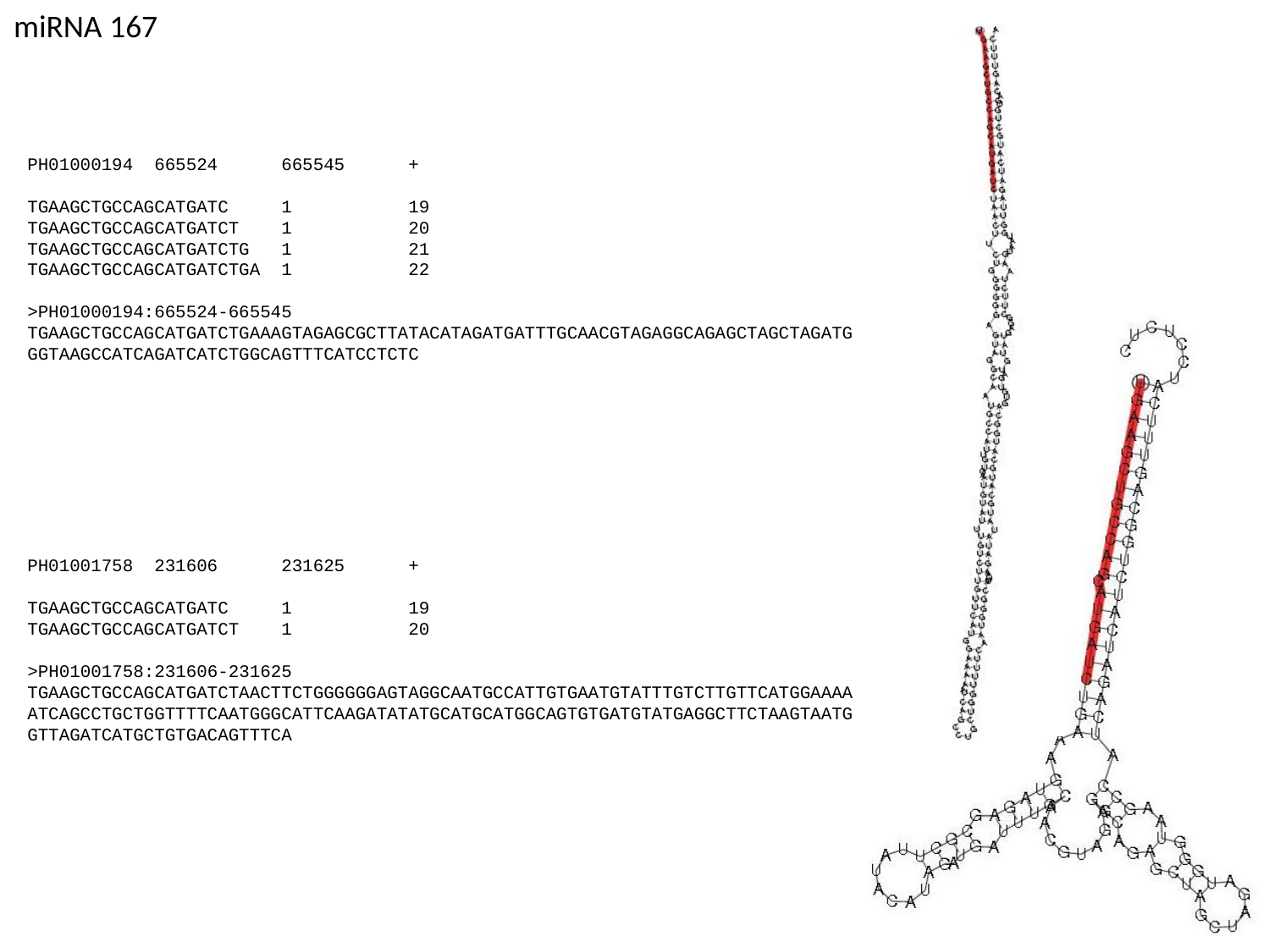

miRNA 167
PH01000194	665524	665545	+
TGAAGCTGCCAGCATGATC 	1	19
TGAAGCTGCCAGCATGATCT 	1	20
TGAAGCTGCCAGCATGATCTG 	1	21
TGAAGCTGCCAGCATGATCTGA 	1	22
>PH01000194:665524-665545
TGAAGCTGCCAGCATGATCTGAAAGTAGAGCGCTTATACATAGATGATTTGCAACGTAGAGGCAGAGCTAGCTAGATGGGTAAGCCATCAGATCATCTGGCAGTTTCATCCTCTC
PH01001758	231606	231625	+
TGAAGCTGCCAGCATGATC 	1	19
TGAAGCTGCCAGCATGATCT 	1	20
>PH01001758:231606-231625
TGAAGCTGCCAGCATGATCTAACTTCTGGGGGGAGTAGGCAATGCCATTGTGAATGTATTTGTCTTGTTCATGGAAAAATCAGCCTGCTGGTTTTCAATGGGCATTCAAGATATATGCATGCATGGCAGTGTGATGTATGAGGCTTCTAAGTAATGGTTAGATCATGCTGTGACAGTTTCA

## Slide 29
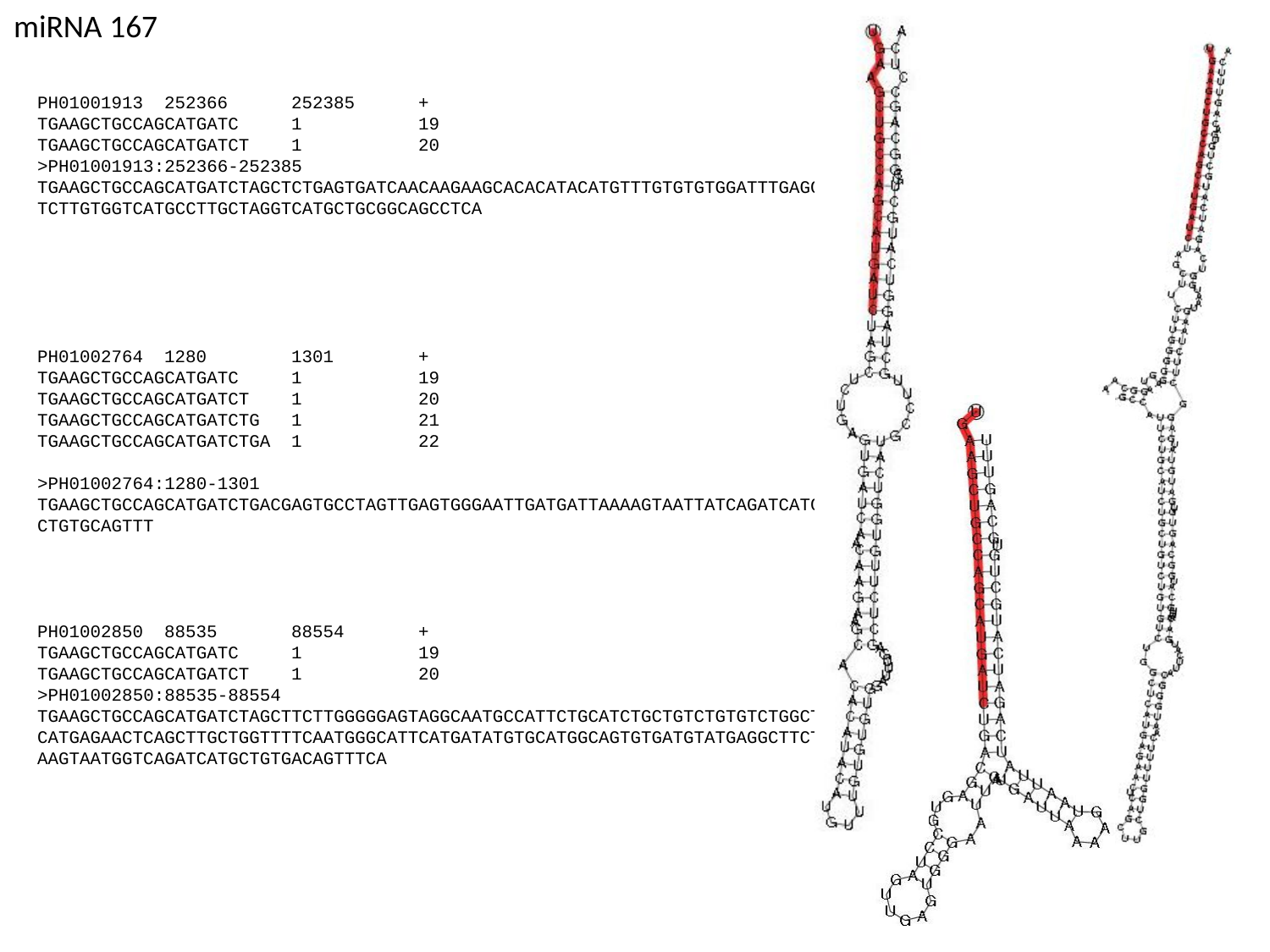

miRNA 167
PH01001913	252366	252385	+
TGAAGCTGCCAGCATGATC 	1	19
TGAAGCTGCCAGCATGATCT 	1	20
>PH01001913:252366-252385
TGAAGCTGCCAGCATGATCTAGCTCTGAGTGATCAACAAGAAGCACACATACATGTTTGTGTGTGGATTTGAGCTCTTGTGGTCATGCCTTGCTAGGTCATGCTGCGGCAGCCTCA
PH01002764	1280	1301	+
TGAAGCTGCCAGCATGATC 	1	19
TGAAGCTGCCAGCATGATCT 	1	20
TGAAGCTGCCAGCATGATCTG 	1	21
TGAAGCTGCCAGCATGATCTGA 	1	22
>PH01002764:1280-1301
TGAAGCTGCCAGCATGATCTGACGAGTGCCTAGTTGAGTGGGAATTGATGATTAAAAGTAATTATCAGATCATGCTGTGCAGTTT
PH01002850	88535	88554	+
TGAAGCTGCCAGCATGATC 	1	19
TGAAGCTGCCAGCATGATCT 	1	20
>PH01002850:88535-88554
TGAAGCTGCCAGCATGATCTAGCTTCTTGGGGGAGTAGGCAATGCCATTCTGCATCTGCTGTCTGTGTCTGGCTCATGAGAACTCAGCTTGCTGGTTTTCAATGGGCATTCATGATATGTGCATGGCAGTGTGATGTATGAGGCTTCTAAGTAATGGTCAGATCATGCTGTGACAGTTTCA

## Slide 30
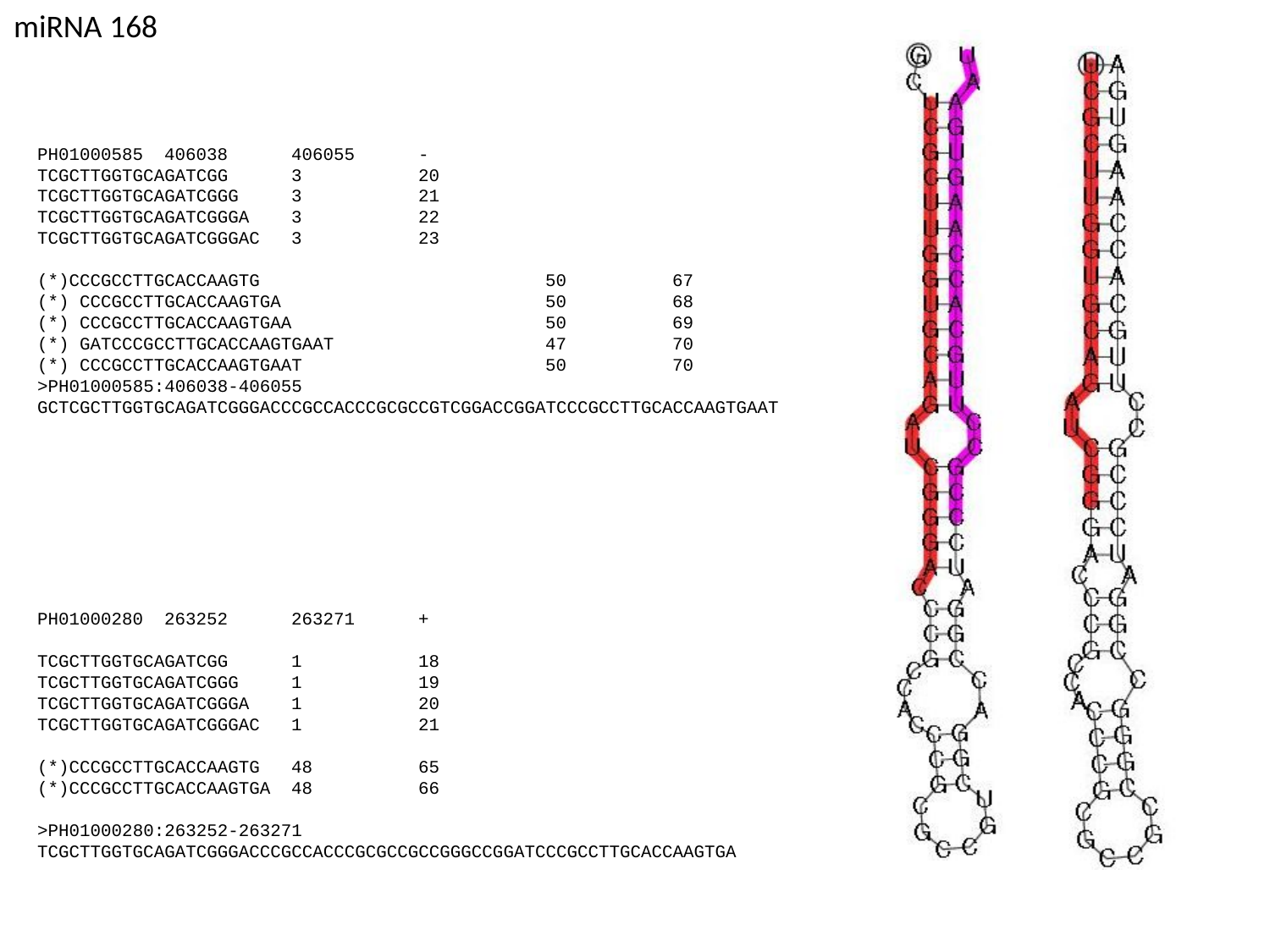

miRNA 168
PH01000585	406038	406055	-
TCGCTTGGTGCAGATCGG 	3	20
TCGCTTGGTGCAGATCGGG 	3	21
TCGCTTGGTGCAGATCGGGA 	3	22
TCGCTTGGTGCAGATCGGGAC 	3	23
(*)CCCGCCTTGCACCAAGTG 			50	67
(*) CCCGCCTTGCACCAAGTGA 		50	68		0
(*) CCCGCCTTGCACCAAGTGAA 		50	69		0
(*) GATCCCGCCTTGCACCAAGTGAAT 		47	70		0
(*) CCCGCCTTGCACCAAGTGAAT 		50	70		0
>PH01000585:406038-406055
GCTCGCTTGGTGCAGATCGGGACCCGCCACCCGCGCCGTCGGACCGGATCCCGCCTTGCACCAAGTGAAT
PH01000280	263252	263271	+
TCGCTTGGTGCAGATCGG 	1	18
TCGCTTGGTGCAGATCGGG 	1	19
TCGCTTGGTGCAGATCGGGA 	1	20
TCGCTTGGTGCAGATCGGGAC 	1	21
(*)CCCGCCTTGCACCAAGTG	48	65
(*)CCCGCCTTGCACCAAGTGA 	48	66
>PH01000280:263252-263271
TCGCTTGGTGCAGATCGGGACCCGCCACCCGCGCCGCCGGGCCGGATCCCGCCTTGCACCAAGTGA

## Slide 31
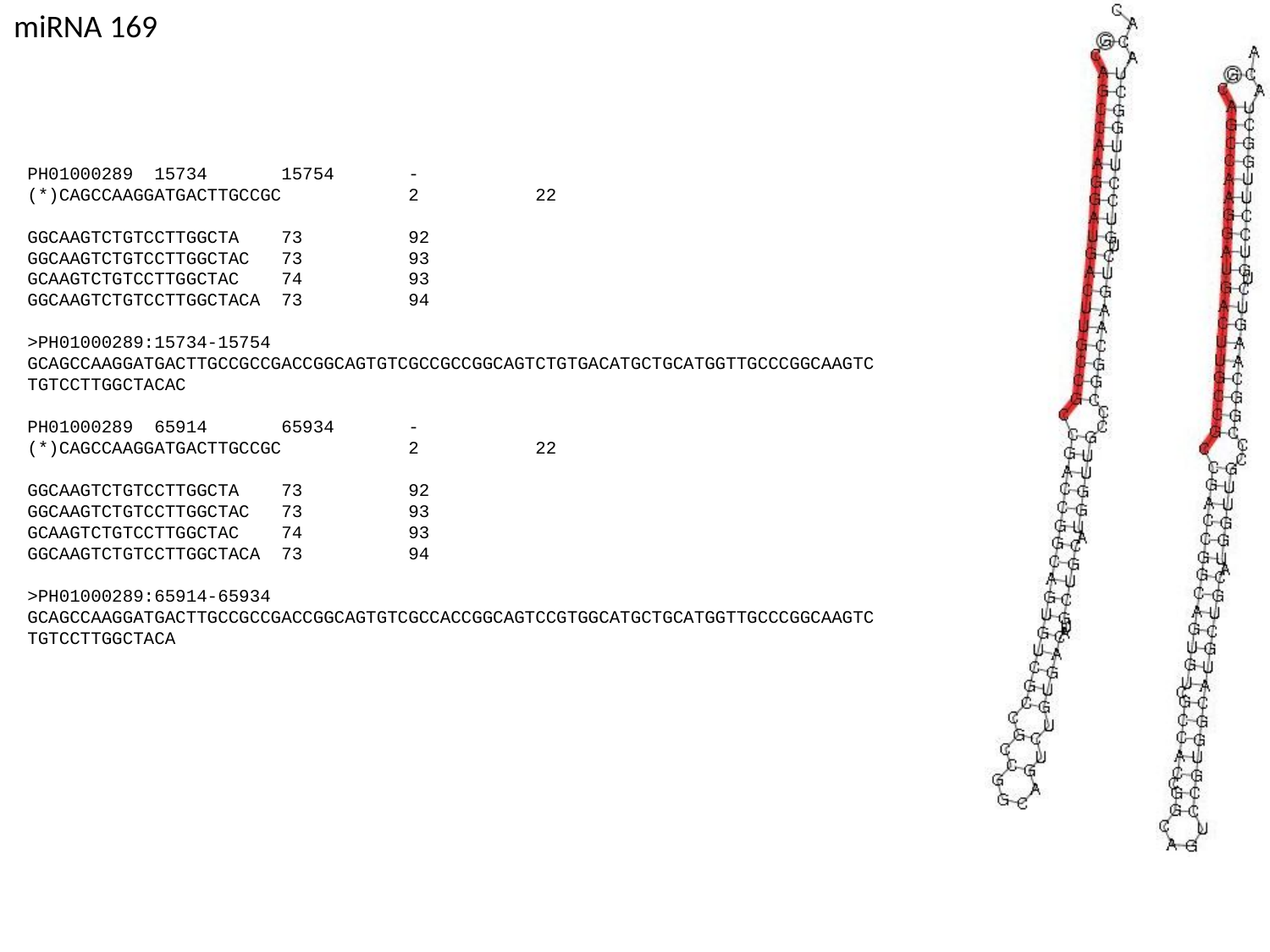

miRNA 169
PH01000289	15734	15754	-
(*)CAGCCAAGGATGACTTGCCGC 	2	22
GGCAAGTCTGTCCTTGGCTA 	73	92
GGCAAGTCTGTCCTTGGCTAC 	73	93
GCAAGTCTGTCCTTGGCTAC 	74	93
GGCAAGTCTGTCCTTGGCTACA 	73	94
>PH01000289:15734-15754
GCAGCCAAGGATGACTTGCCGCCGACCGGCAGTGTCGCCGCCGGCAGTCTGTGACATGCTGCATGGTTGCCCGGCAAGTCTGTCCTTGGCTACAC
PH01000289	65914	65934	-
(*)CAGCCAAGGATGACTTGCCGC 	2	22
GGCAAGTCTGTCCTTGGCTA 	73	92
GGCAAGTCTGTCCTTGGCTAC 	73	93
GCAAGTCTGTCCTTGGCTAC 	74	93
GGCAAGTCTGTCCTTGGCTACA 	73	94
>PH01000289:65914-65934
GCAGCCAAGGATGACTTGCCGCCGACCGGCAGTGTCGCCACCGGCAGTCCGTGGCATGCTGCATGGTTGCCCGGCAAGTCTGTCCTTGGCTACA

## Slide 32
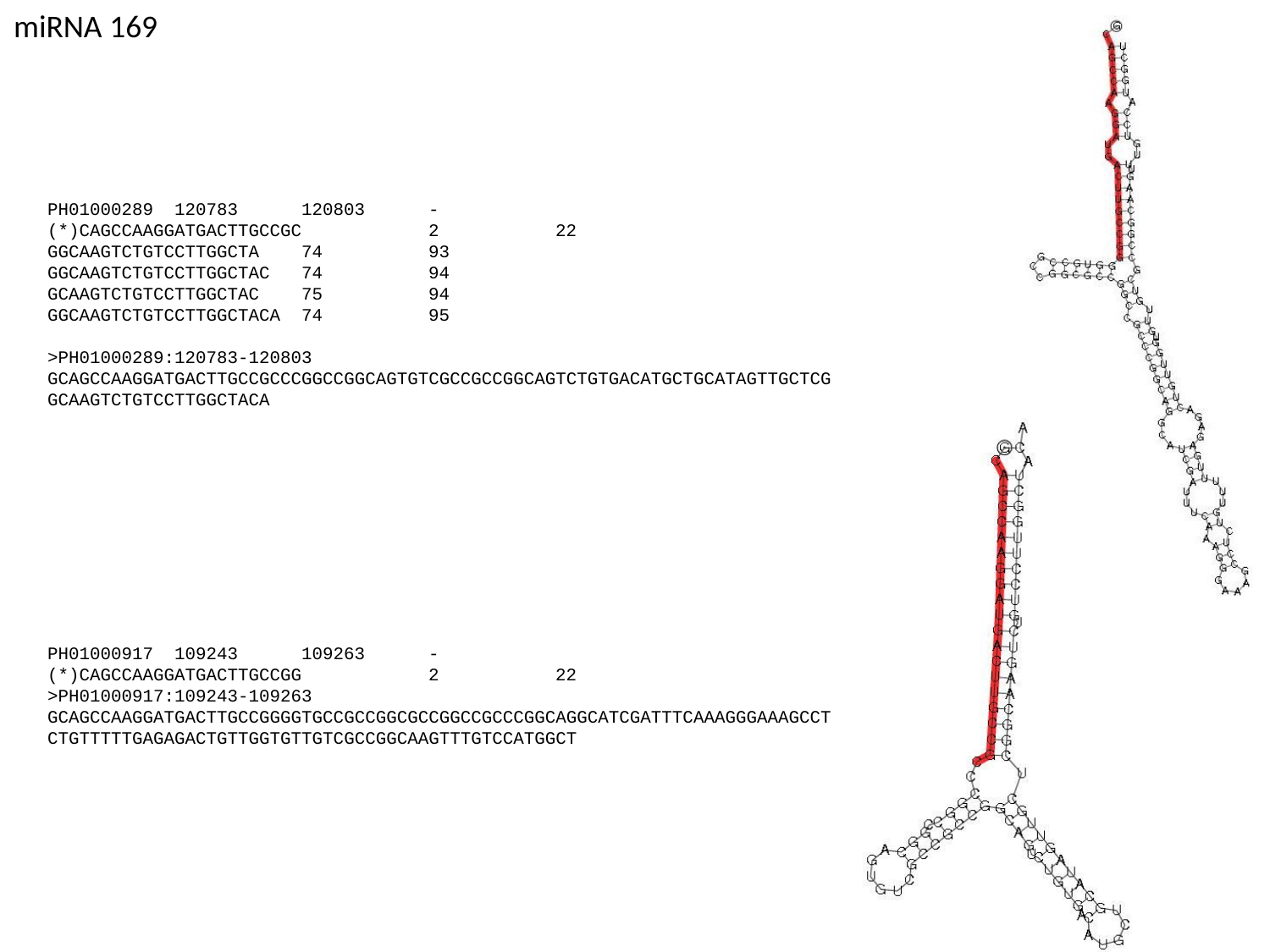

miRNA 169
PH01000289	120783	120803	-
(*)CAGCCAAGGATGACTTGCCGC 	2	22
GGCAAGTCTGTCCTTGGCTA 	74	93
GGCAAGTCTGTCCTTGGCTAC 	74	94
GCAAGTCTGTCCTTGGCTAC 	75	94
GGCAAGTCTGTCCTTGGCTACA 	74	95
>PH01000289:120783-120803
GCAGCCAAGGATGACTTGCCGCCCGGCCGGCAGTGTCGCCGCCGGCAGTCTGTGACATGCTGCATAGTTGCTCGGCAAGTCTGTCCTTGGCTACA
PH01000917	109243	109263	-
(*)CAGCCAAGGATGACTTGCCGG 	2	22
>PH01000917:109243-109263
GCAGCCAAGGATGACTTGCCGGGGTGCCGCCGGCGCCGGCCGCCCGGCAGGCATCGATTTCAAAGGGAAAGCCTCTGTTTTTGAGAGACTGTTGGTGTTGTCGCCGGCAAGTTTGTCCATGGCT

## Slide 33
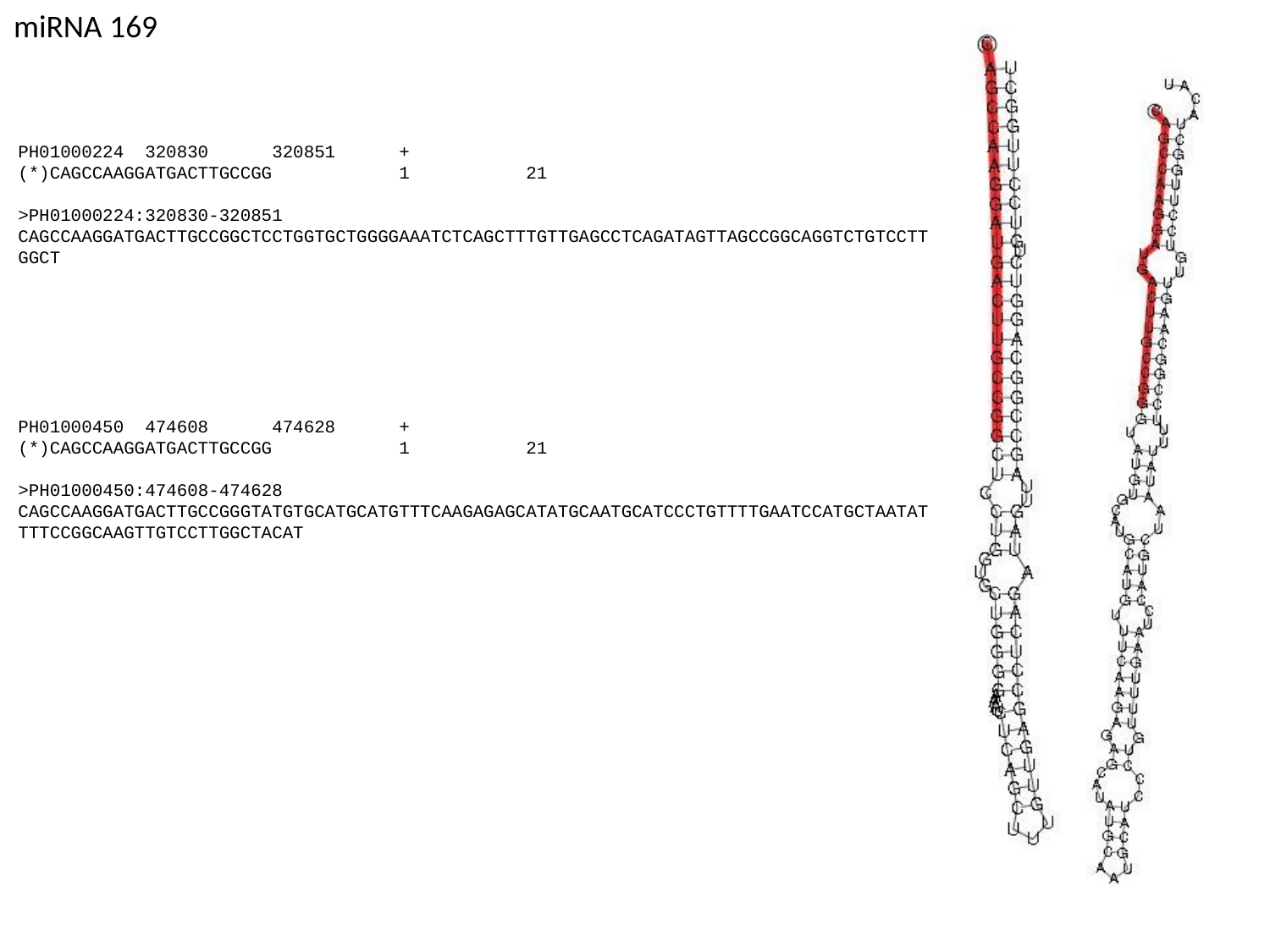

miRNA 169
PH01000224	320830	320851	+
(*)CAGCCAAGGATGACTTGCCGG 	1	21
>PH01000224:320830-320851
CAGCCAAGGATGACTTGCCGGCTCCTGGTGCTGGGGAAATCTCAGCTTTGTTGAGCCTCAGATAGTTAGCCGGCAGGTCTGTCCTTGGCT
PH01000450	474608	474628	+
(*)CAGCCAAGGATGACTTGCCGG 	1	21
>PH01000450:474608-474628
CAGCCAAGGATGACTTGCCGGGTATGTGCATGCATGTTTCAAGAGAGCATATGCAATGCATCCCTGTTTTGAATCCATGCTAATATTTTCCGGCAAGTTGTCCTTGGCTACAT

## Slide 34
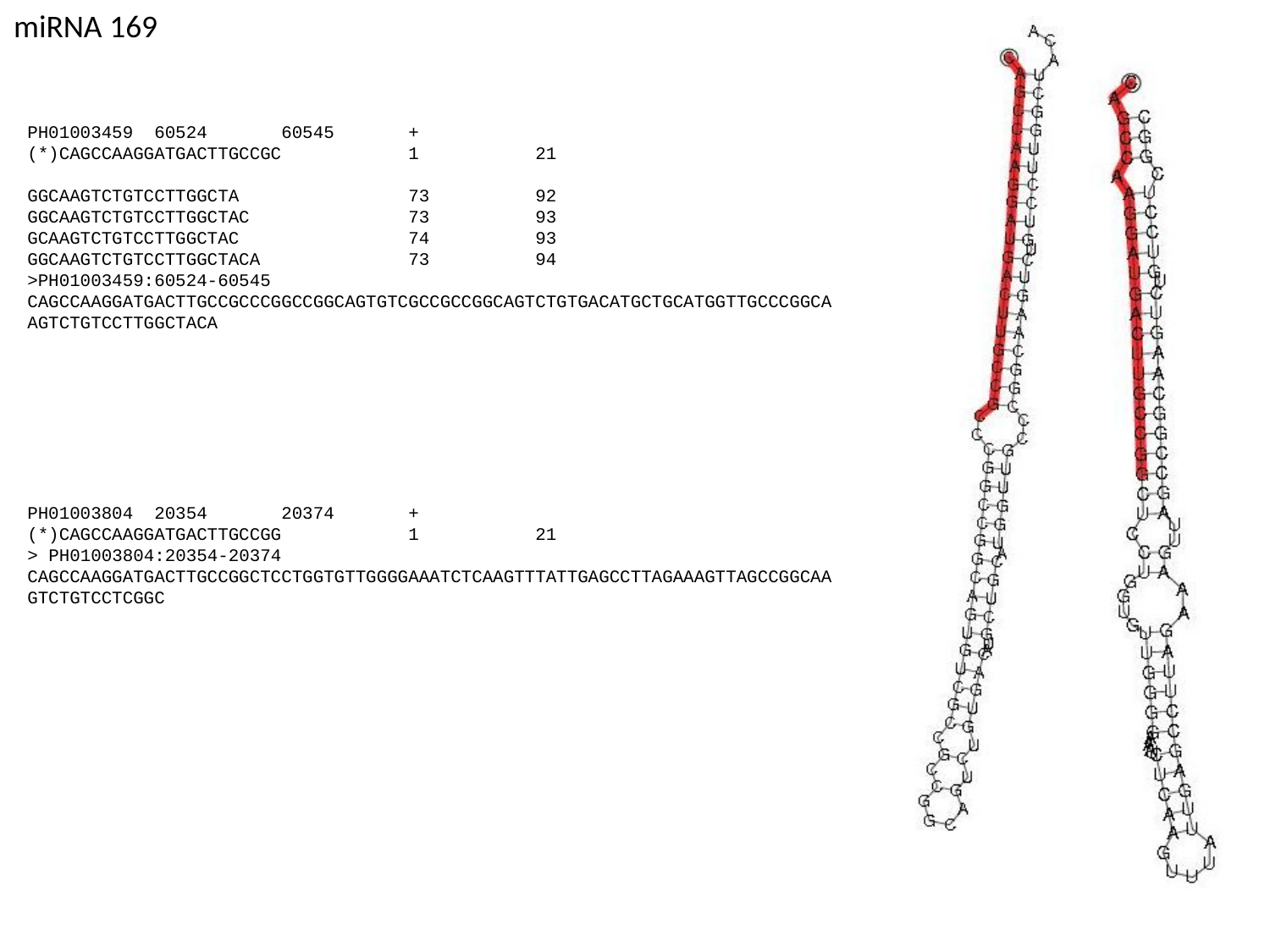

miRNA 169
PH01003459	60524	60545	+
(*)CAGCCAAGGATGACTTGCCGC 	1	21
GGCAAGTCTGTCCTTGGCTA 		73	92
GGCAAGTCTGTCCTTGGCTAC 		73	93
GCAAGTCTGTCCTTGGCTAC 		74	93
GGCAAGTCTGTCCTTGGCTACA 		73	94
>PH01003459:60524-60545
CAGCCAAGGATGACTTGCCGCCCGGCCGGCAGTGTCGCCGCCGGCAGTCTGTGACATGCTGCATGGTTGCCCGGCAAGTCTGTCCTTGGCTACA
PH01003804	20354	20374	+
(*)CAGCCAAGGATGACTTGCCGG 	1	21
> PH01003804:20354-20374
CAGCCAAGGATGACTTGCCGGCTCCTGGTGTTGGGGAAATCTCAAGTTTATTGAGCCTTAGAAAGTTAGCCGGCAAGTCTGTCCTCGGC

## Slide 35
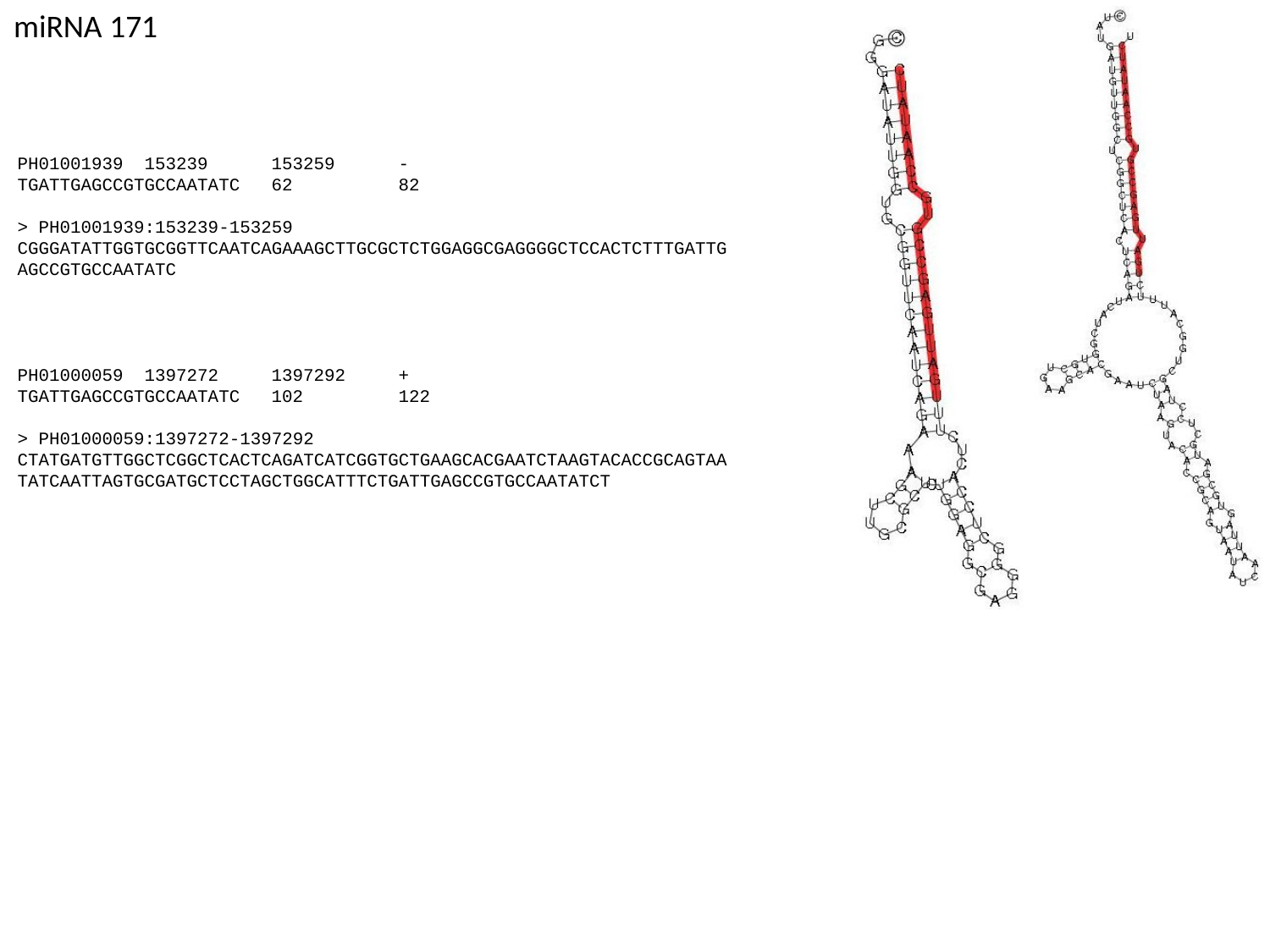

miRNA 171
PH01001939	153239	153259	-
TGATTGAGCCGTGCCAATATC 	62	82
> PH01001939:153239-153259
CGGGATATTGGTGCGGTTCAATCAGAAAGCTTGCGCTCTGGAGGCGAGGGGCTCCACTCTTTGATTGAGCCGTGCCAATATC
PH01000059	1397272	1397292	+
TGATTGAGCCGTGCCAATATC 	102	122
> PH01000059:1397272-1397292
CTATGATGTTGGCTCGGCTCACTCAGATCATCGGTGCTGAAGCACGAATCTAAGTACACCGCAGTAATATCAATTAGTGCGATGCTCCTAGCTGGCATTTCTGATTGAGCCGTGCCAATATCT

## Slide 36
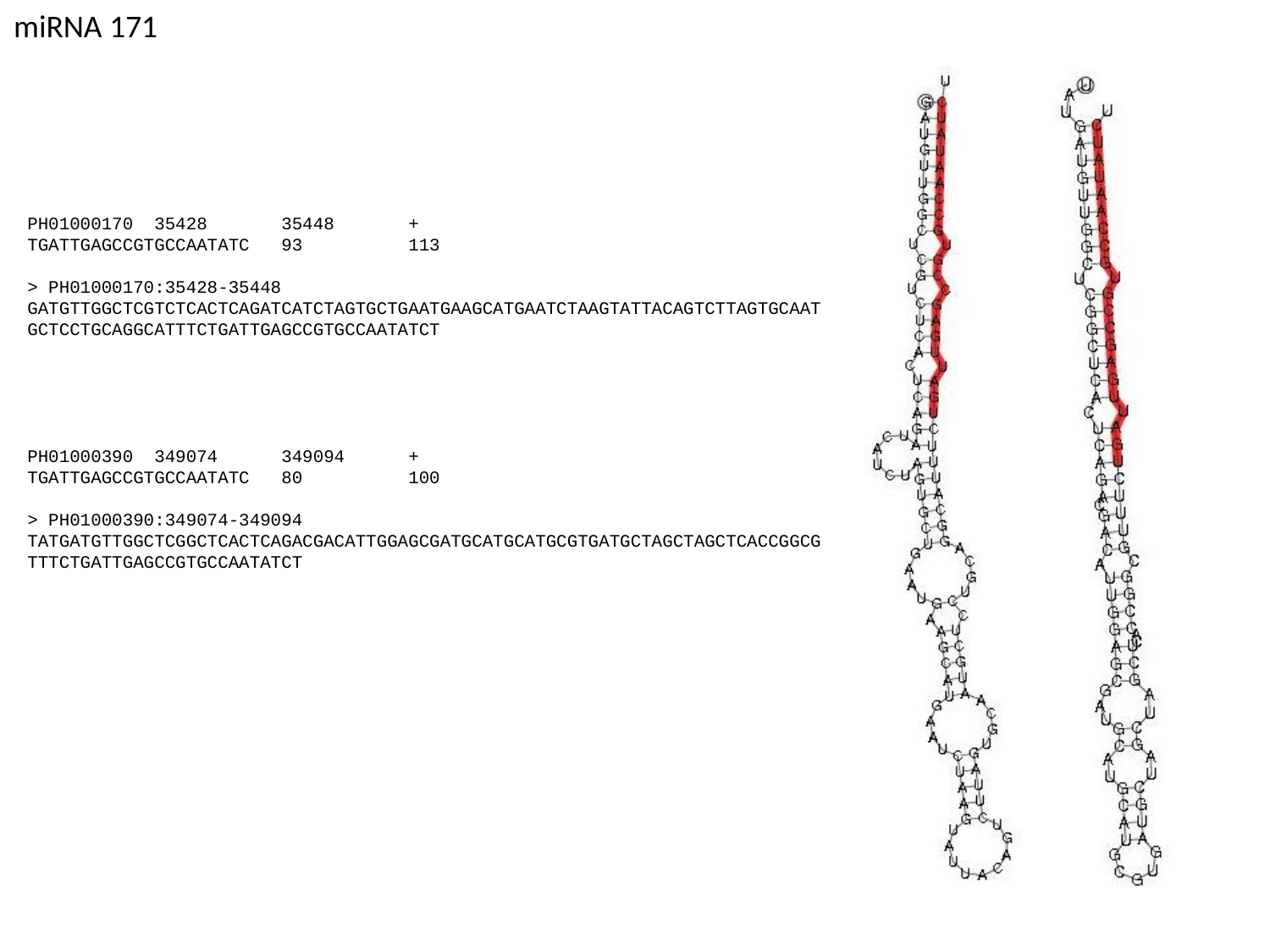

miRNA 171
PH01000170	35428	35448	+
TGATTGAGCCGTGCCAATATC 	93	113
> PH01000170:35428-35448
GATGTTGGCTCGTCTCACTCAGATCATCTAGTGCTGAATGAAGCATGAATCTAAGTATTACAGTCTTAGTGCAATGCTCCTGCAGGCATTTCTGATTGAGCCGTGCCAATATCT
PH01000390	349074	349094	+
TGATTGAGCCGTGCCAATATC 	80	100
> PH01000390:349074-349094
TATGATGTTGGCTCGGCTCACTCAGACGACATTGGAGCGATGCATGCATGCGTGATGCTAGCTAGCTCACCGGCGTTTCTGATTGAGCCGTGCCAATATCT

## Slide 37
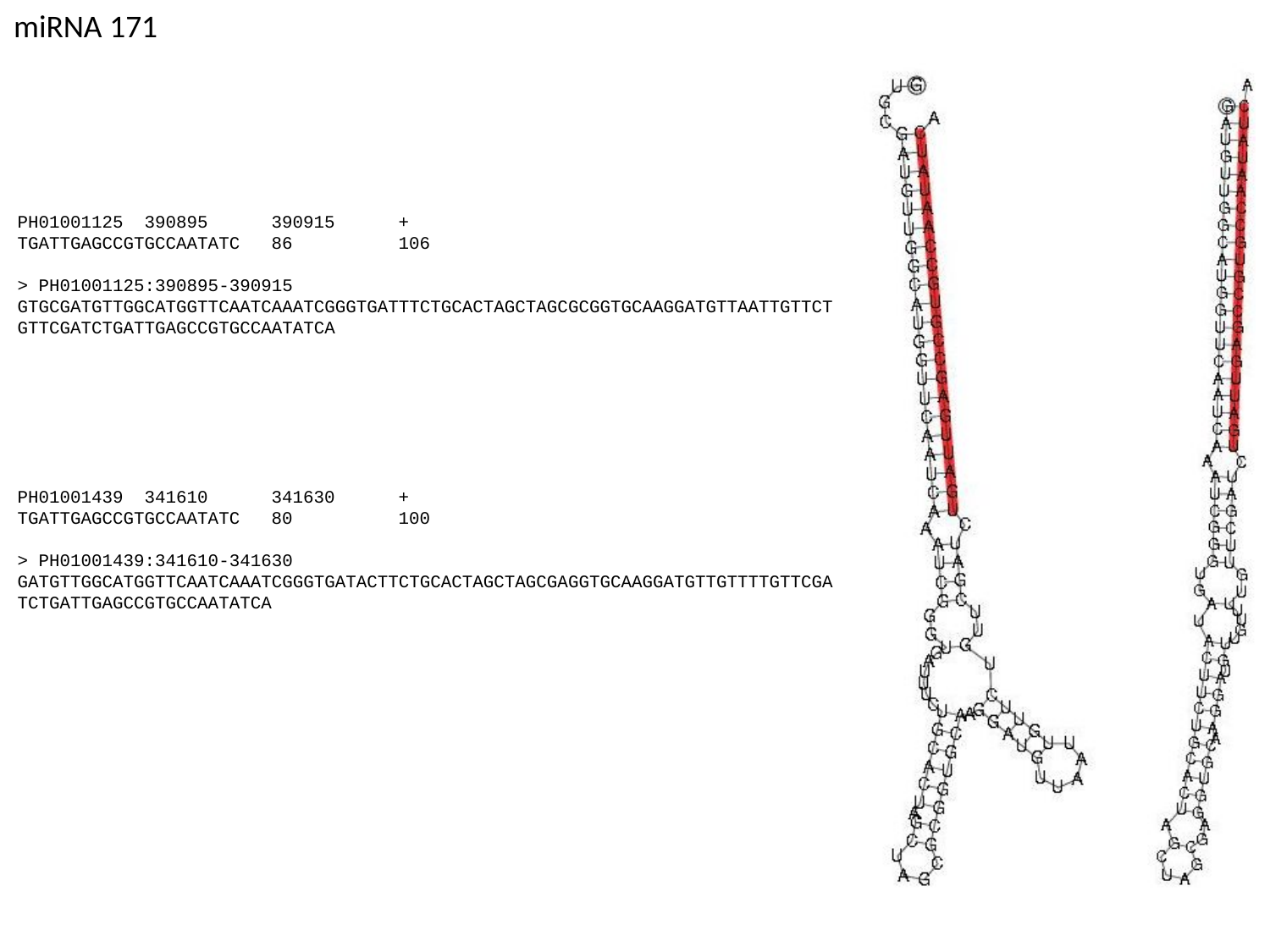

miRNA 171
PH01001125	390895	390915	+
TGATTGAGCCGTGCCAATATC 	86	106
> PH01001125:390895-390915
GTGCGATGTTGGCATGGTTCAATCAAATCGGGTGATTTCTGCACTAGCTAGCGCGGTGCAAGGATGTTAATTGTTCTGTTCGATCTGATTGAGCCGTGCCAATATCA
PH01001439	341610	341630	+
TGATTGAGCCGTGCCAATATC 	80	100
> PH01001439:341610-341630
GATGTTGGCATGGTTCAATCAAATCGGGTGATACTTCTGCACTAGCTAGCGAGGTGCAAGGATGTTGTTTTGTTCGATCTGATTGAGCCGTGCCAATATCA

## Slide 38
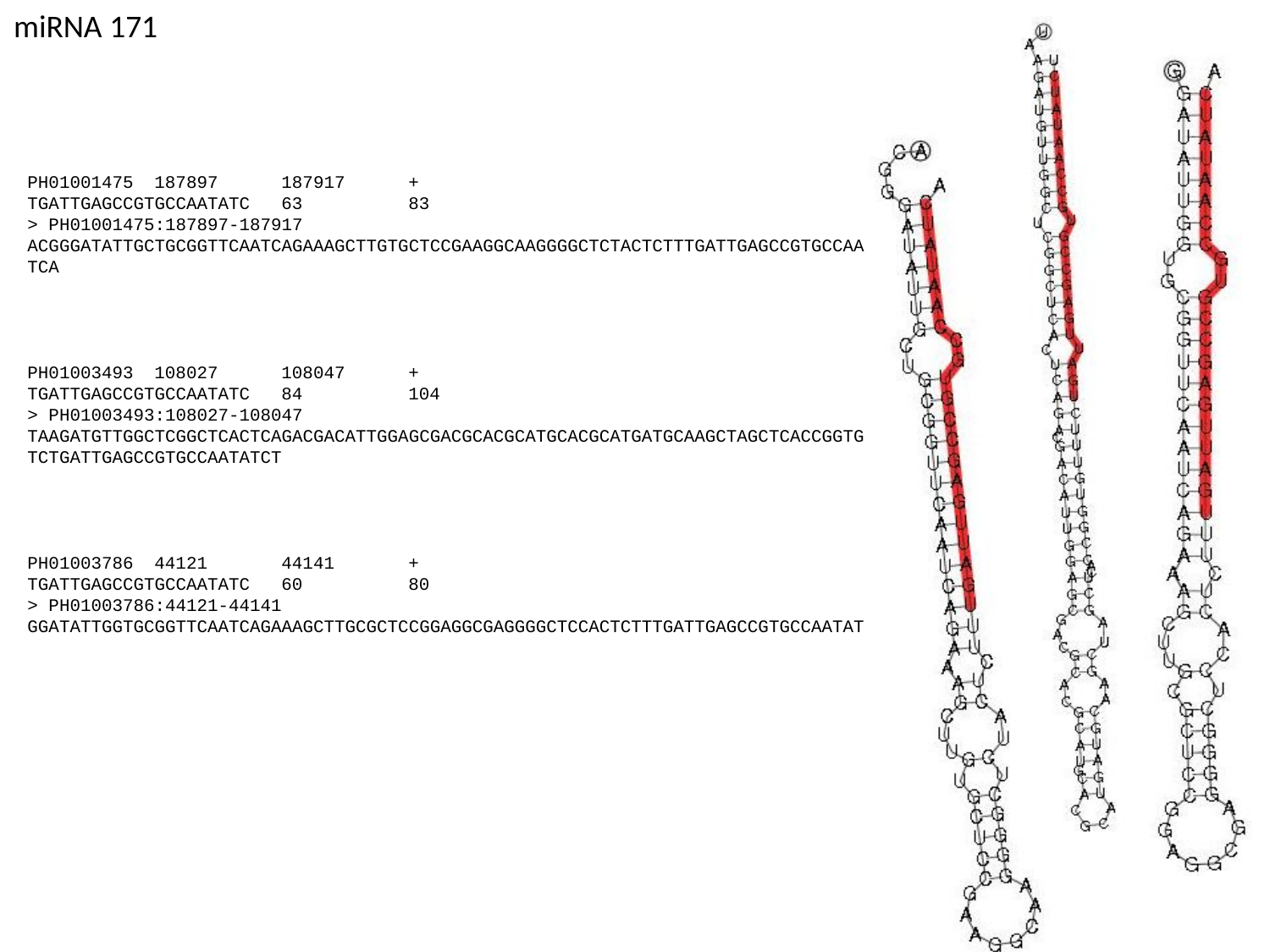

miRNA 171
PH01001475	187897	187917	+
TGATTGAGCCGTGCCAATATC 	63	83
> PH01001475:187897-187917 ACGGGATATTGCTGCGGTTCAATCAGAAAGCTTGTGCTCCGAAGGCAAGGGGCTCTACTCTTTGATTGAGCCGTGCCAATATCA
PH01003493	108027	108047	+
TGATTGAGCCGTGCCAATATC 	84	104
> PH01003493:108027-108047
TAAGATGTTGGCTCGGCTCACTCAGACGACATTGGAGCGACGCACGCATGCACGCATGATGCAAGCTAGCTCACCGGTGTTTCTGATTGAGCCGTGCCAATATCT
PH01003786	44121	44141	+
TGATTGAGCCGTGCCAATATC 	60	80
> PH01003786:44121-44141
GGATATTGGTGCGGTTCAATCAGAAAGCTTGCGCTCCGGAGGCGAGGGGCTCCACTCTTTGATTGAGCCGTGCCAATATCA

## Slide 39
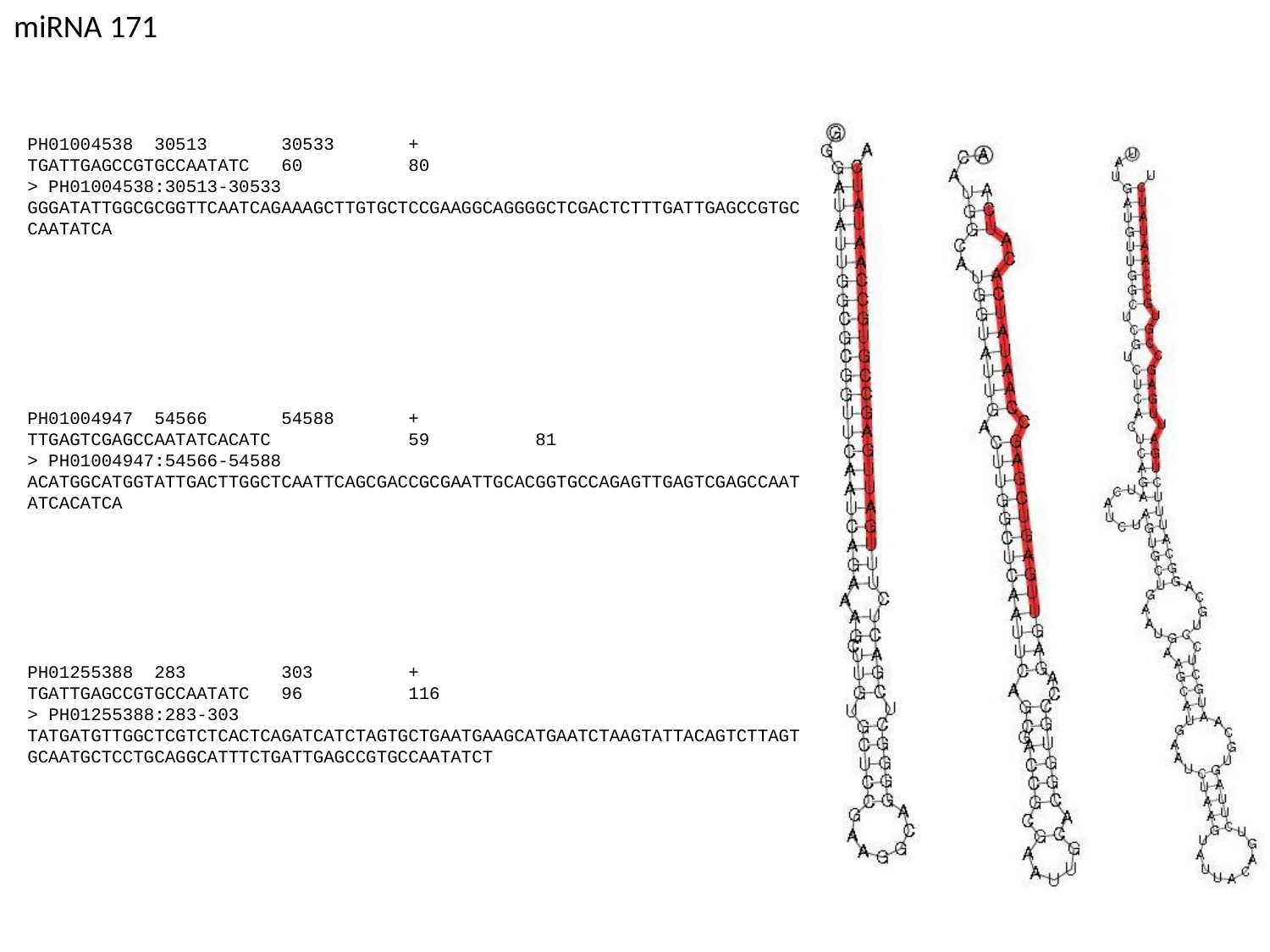

miRNA 171
PH01004538	30513	30533	+
TGATTGAGCCGTGCCAATATC 	60	80
> PH01004538:30513-30533
GGGATATTGGCGCGGTTCAATCAGAAAGCTTGTGCTCCGAAGGCAGGGGCTCGACTCTTTGATTGAGCCGTGCCAATATCA
PH01004947	54566	54588	+
TTGAGTCGAGCCAATATCACATC 	59	81
> PH01004947:54566-54588
ACATGGCATGGTATTGACTTGGCTCAATTCAGCGACCGCGAATTGCACGGTGCCAGAGTTGAGTCGAGCCAATATCACATCA
PH01255388	283	303	+
TGATTGAGCCGTGCCAATATC 	96	116
> PH01255388:283-303
TATGATGTTGGCTCGTCTCACTCAGATCATCTAGTGCTGAATGAAGCATGAATCTAAGTATTACAGTCTTAGTGCAATGCTCCTGCAGGCATTTCTGATTGAGCCGTGCCAATATCT

## Slide 40
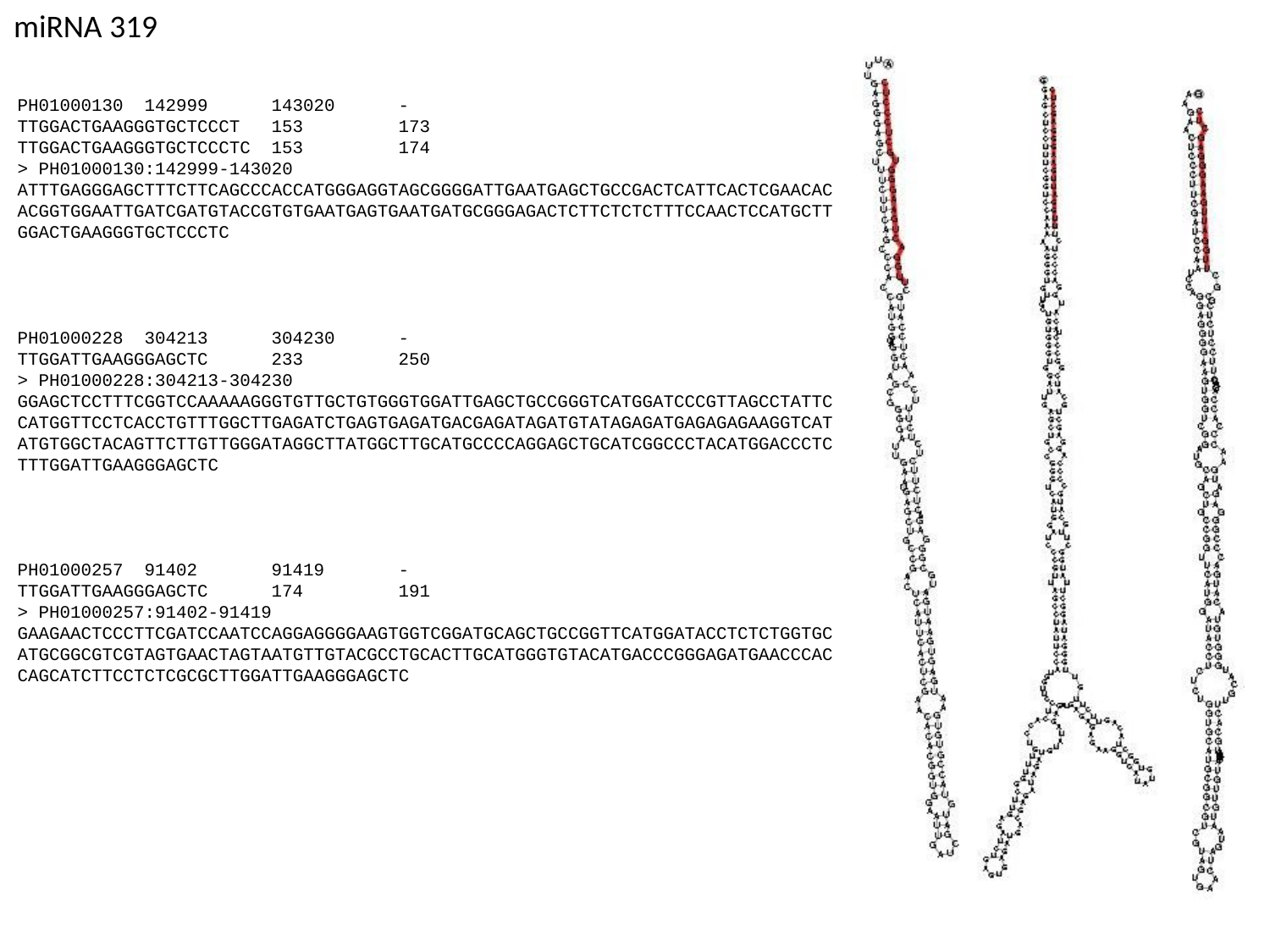

miRNA 319
PH01000130	142999	143020	-
TTGGACTGAAGGGTGCTCCCT 	153	173
TTGGACTGAAGGGTGCTCCCTC 	153	174
> PH01000130:142999-143020
ATTTGAGGGAGCTTTCTTCAGCCCACCATGGGAGGTAGCGGGGATTGAATGAGCTGCCGACTCATTCACTCGAACACACGGTGGAATTGATCGATGTACCGTGTGAATGAGTGAATGATGCGGGAGACTCTTCTCTCTTTCCAACTCCATGCTTGGACTGAAGGGTGCTCCCTC
PH01000228	304213	304230	-
TTGGATTGAAGGGAGCTC 	233	250
> PH01000228:304213-304230
GGAGCTCCTTTCGGTCCAAAAAGGGTGTTGCTGTGGGTGGATTGAGCTGCCGGGTCATGGATCCCGTTAGCCTATTCCATGGTTCCTCACCTGTTTGGCTTGAGATCTGAGTGAGATGACGAGATAGATGTATAGAGATGAGAGAGAAGGTCATATGTGGCTACAGTTCTTGTTGGGATAGGCTTATGGCTTGCATGCCCCAGGAGCTGCATCGGCCCTACATGGACCCTCTTTGGATTGAAGGGAGCTC
PH01000257	91402	91419	-
TTGGATTGAAGGGAGCTC 	174	191
> PH01000257:91402-91419
GAAGAACTCCCTTCGATCCAATCCAGGAGGGGAAGTGGTCGGATGCAGCTGCCGGTTCATGGATACCTCTCTGGTGCATGCGGCGTCGTAGTGAACTAGTAATGTTGTACGCCTGCACTTGCATGGGTGTACATGACCCGGGAGATGAACCCACCAGCATCTTCCTCTCGCGCTTGGATTGAAGGGAGCTC

## Slide 41
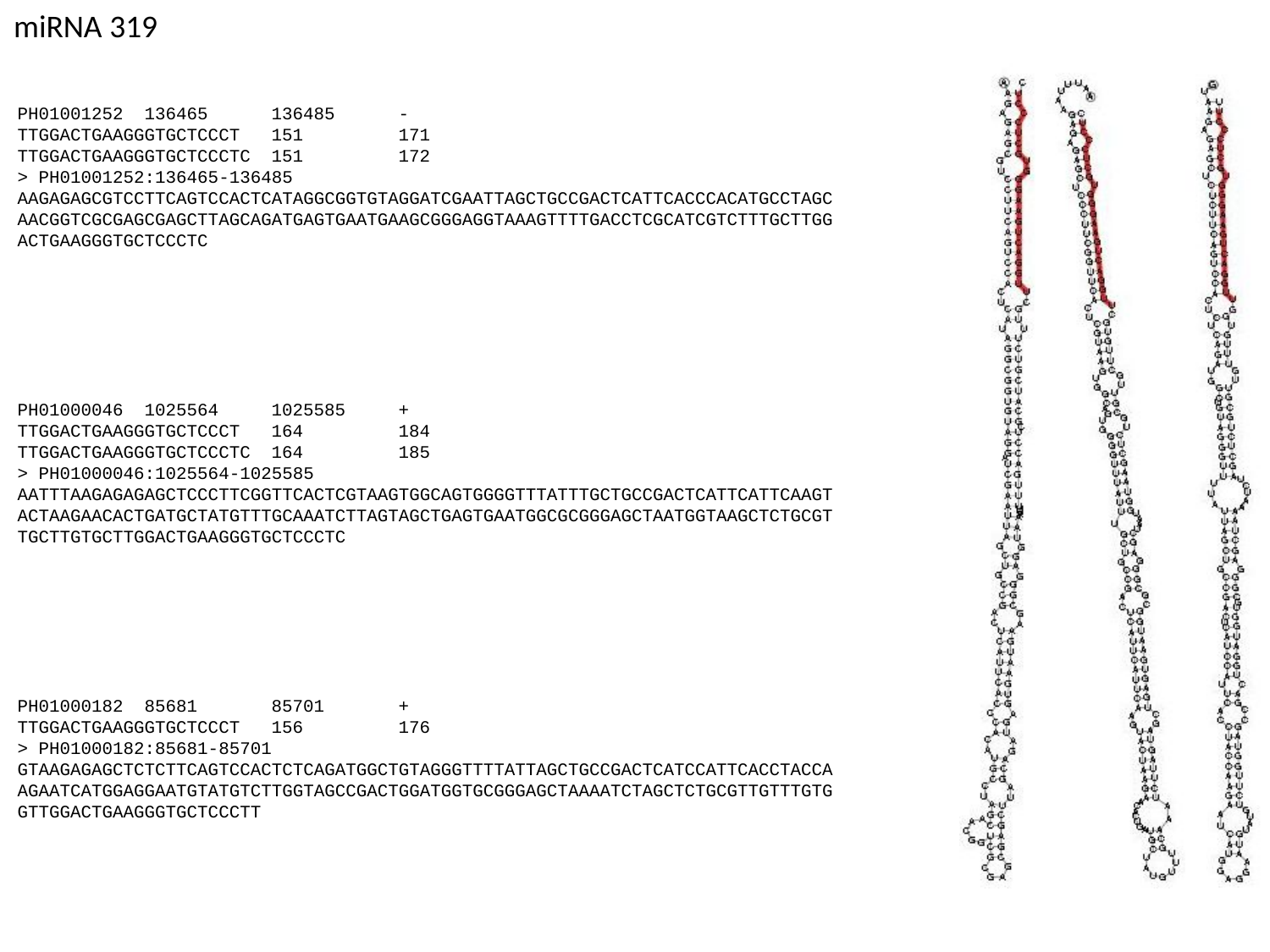

miRNA 319
PH01001252	136465	136485	-
TTGGACTGAAGGGTGCTCCCT 	151	171
TTGGACTGAAGGGTGCTCCCTC 	151	172
> PH01001252:136465-136485
AAGAGAGCGTCCTTCAGTCCACTCATAGGCGGTGTAGGATCGAATTAGCTGCCGACTCATTCACCCACATGCCTAGCAACGGTCGCGAGCGAGCTTAGCAGATGAGTGAATGAAGCGGGAGGTAAAGTTTTGACCTCGCATCGTCTTTGCTTGGACTGAAGGGTGCTCCCTC
PH01000046	1025564	1025585	+
TTGGACTGAAGGGTGCTCCCT 	164	184
TTGGACTGAAGGGTGCTCCCTC 	164	185
> PH01000046:1025564-1025585
AATTTAAGAGAGAGCTCCCTTCGGTTCACTCGTAAGTGGCAGTGGGGTTTATTTGCTGCCGACTCATTCATTCAAGTACTAAGAACACTGATGCTATGTTTGCAAATCTTAGTAGCTGAGTGAATGGCGCGGGAGCTAATGGTAAGCTCTGCGTTGCTTGTGCTTGGACTGAAGGGTGCTCCCTC
PH01000182	85681	85701	+
TTGGACTGAAGGGTGCTCCCT 	156	176
> PH01000182:85681-85701
GTAAGAGAGCTCTCTTCAGTCCACTCTCAGATGGCTGTAGGGTTTTATTAGCTGCCGACTCATCCATTCACCTACCAAGAATCATGGAGGAATGTATGTCTTGGTAGCCGACTGGATGGTGCGGGAGCTAAAATCTAGCTCTGCGTTGTTTGTGGTTGGACTGAAGGGTGCTCCCTT

## Slide 42
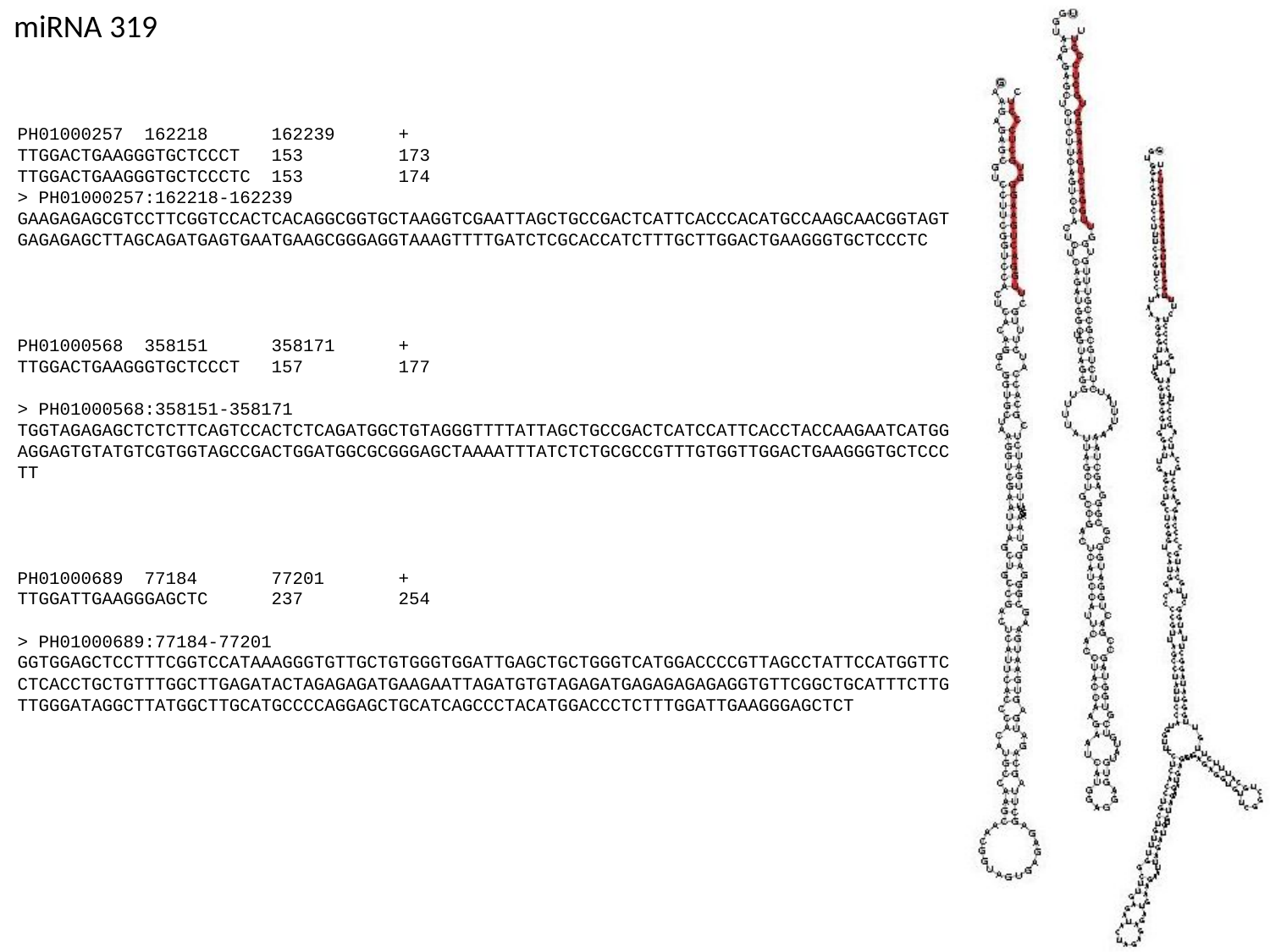

miRNA 319
PH01000257	162218	162239	+
TTGGACTGAAGGGTGCTCCCT 	153	173
TTGGACTGAAGGGTGCTCCCTC 	153	174
> PH01000257:162218-162239
GAAGAGAGCGTCCTTCGGTCCACTCACAGGCGGTGCTAAGGTCGAATTAGCTGCCGACTCATTCACCCACATGCCAAGCAACGGTAGTGAGAGAGCTTAGCAGATGAGTGAATGAAGCGGGAGGTAAAGTTTTGATCTCGCACCATCTTTGCTTGGACTGAAGGGTGCTCCCTC
PH01000568	358151	358171	+
TTGGACTGAAGGGTGCTCCCT 	157	177
> PH01000568:358151-358171
TGGTAGAGAGCTCTCTTCAGTCCACTCTCAGATGGCTGTAGGGTTTTATTAGCTGCCGACTCATCCATTCACCTACCAAGAATCATGGAGGAGTGTATGTCGTGGTAGCCGACTGGATGGCGCGGGAGCTAAAATTTATCTCTGCGCCGTTTGTGGTTGGACTGAAGGGTGCTCCCTT
PH01000689	77184	77201	+
TTGGATTGAAGGGAGCTC 	237	254
> PH01000689:77184-77201
GGTGGAGCTCCTTTCGGTCCATAAAGGGTGTTGCTGTGGGTGGATTGAGCTGCTGGGTCATGGACCCCGTTAGCCTATTCCATGGTTCCTCACCTGCTGTTTGGCTTGAGATACTAGAGAGATGAAGAATTAGATGTGTAGAGATGAGAGAGAGAGGTGTTCGGCTGCATTTCTTGTTGGGATAGGCTTATGGCTTGCATGCCCCAGGAGCTGCATCAGCCCTACATGGACCCTCTTTGGATTGAAGGGAGCTCT

## Slide 43
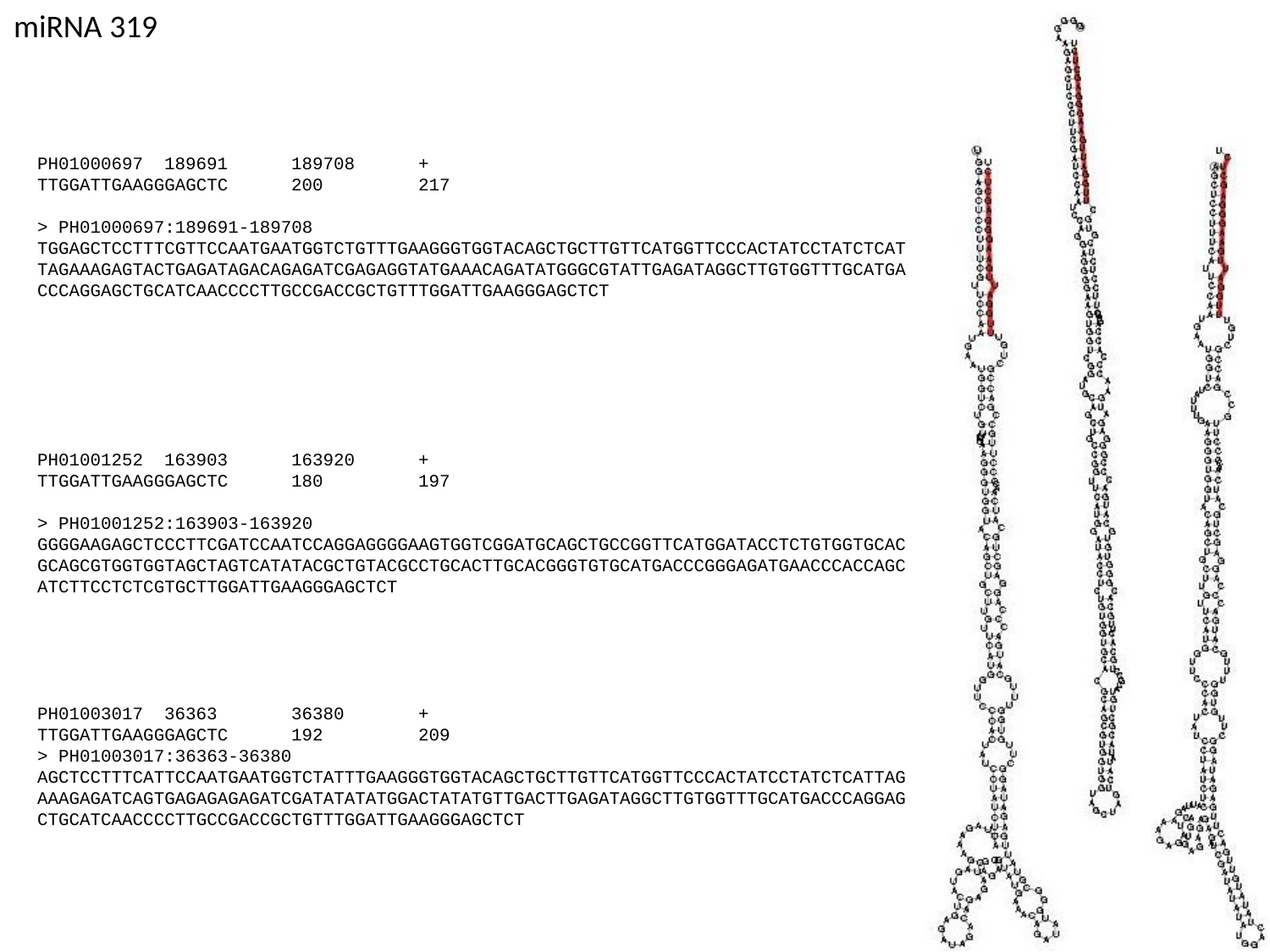

miRNA 319
PH01000697	189691	189708	+
TTGGATTGAAGGGAGCTC 	200	217
> PH01000697:189691-189708
TGGAGCTCCTTTCGTTCCAATGAATGGTCTGTTTGAAGGGTGGTACAGCTGCTTGTTCATGGTTCCCACTATCCTATCTCATTAGAAAGAGTACTGAGATAGACAGAGATCGAGAGGTATGAAACAGATATGGGCGTATTGAGATAGGCTTGTGGTTTGCATGACCCAGGAGCTGCATCAACCCCTTGCCGACCGCTGTTTGGATTGAAGGGAGCTCT
PH01001252	163903	163920	+
TTGGATTGAAGGGAGCTC 	180	197
> PH01001252:163903-163920
GGGGAAGAGCTCCCTTCGATCCAATCCAGGAGGGGAAGTGGTCGGATGCAGCTGCCGGTTCATGGATACCTCTGTGGTGCACGCAGCGTGGTGGTAGCTAGTCATATACGCTGTACGCCTGCACTTGCACGGGTGTGCATGACCCGGGAGATGAACCCACCAGCATCTTCCTCTCGTGCTTGGATTGAAGGGAGCTCT
PH01003017	36363	36380	+
TTGGATTGAAGGGAGCTC 	192	209
> PH01003017:36363-36380
AGCTCCTTTCATTCCAATGAATGGTCTATTTGAAGGGTGGTACAGCTGCTTGTTCATGGTTCCCACTATCCTATCTCATTAGAAAGAGATCAGTGAGAGAGAGATCGATATATATGGACTATATGTTGACTTGAGATAGGCTTGTGGTTTGCATGACCCAGGAGCTGCATCAACCCCTTGCCGACCGCTGTTTGGATTGAAGGGAGCTCT

## Slide 44
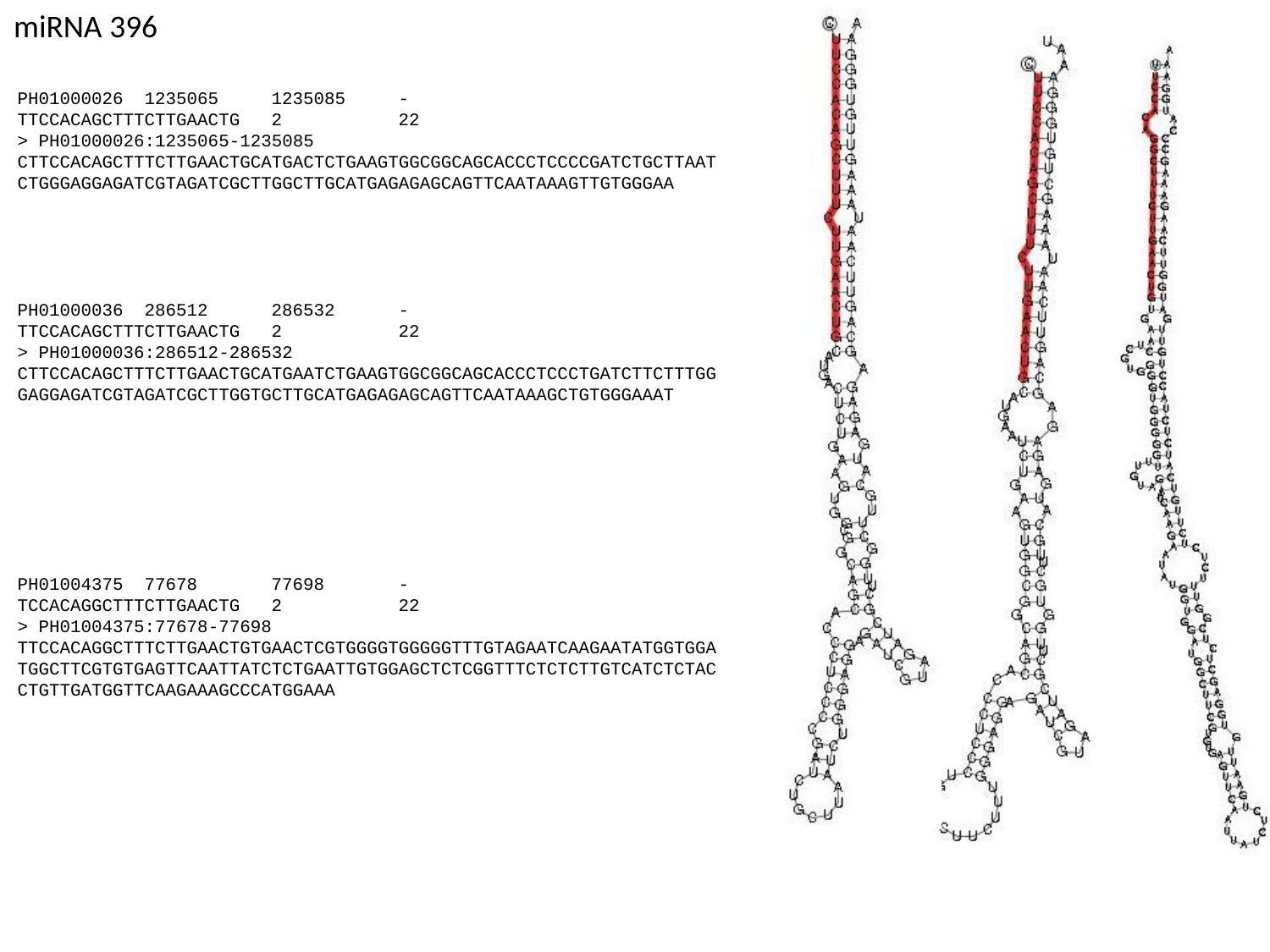

miRNA 396
PH01000026	1235065	1235085	-
TTCCACAGCTTTCTTGAACTG 	2	22
> PH01000026:1235065-1235085
CTTCCACAGCTTTCTTGAACTGCATGACTCTGAAGTGGCGGCAGCACCCTCCCCGATCTGCTTAATCTGGGAGGAGATCGTAGATCGCTTGGCTTGCATGAGAGAGCAGTTCAATAAAGTTGTGGGAA
PH01000036	286512	286532	-
TTCCACAGCTTTCTTGAACTG 	2	22
> PH01000036:286512-286532
CTTCCACAGCTTTCTTGAACTGCATGAATCTGAAGTGGCGGCAGCACCCTCCCTGATCTTCTTTGGGAGGAGATCGTAGATCGCTTGGTGCTTGCATGAGAGAGCAGTTCAATAAAGCTGTGGGAAAT
PH01004375	77678	77698	-
TCCACAGGCTTTCTTGAACTG 	2	22
> PH01004375:77678-77698
TTCCACAGGCTTTCTTGAACTGTGAACTCGTGGGGTGGGGGTTTGTAGAATCAAGAATATGGTGGATGGCTTCGTGTGAGTTCAATTATCTCTGAATTGTGGAGCTCTCGGTTTCTCTCTTGTCATCTCTACCTGTTGATGGTTCAAGAAAGCCCATGGAAA

## Slide 45
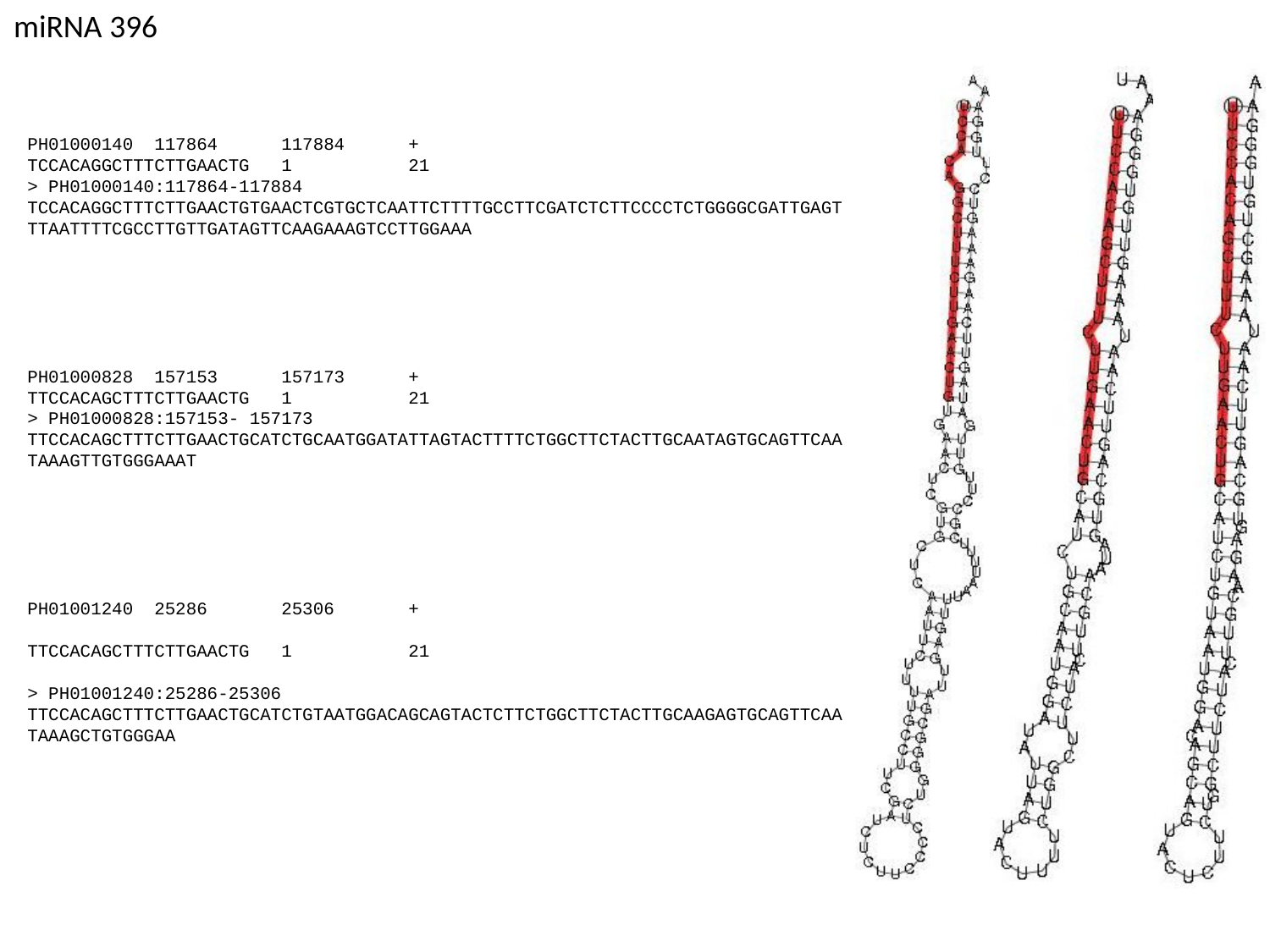

miRNA 396
PH01000140	117864	117884	+
TCCACAGGCTTTCTTGAACTG 	1	21
> PH01000140:117864-117884
TCCACAGGCTTTCTTGAACTGTGAACTCGTGCTCAATTCTTTTGCCTTCGATCTCTTCCCCTCTGGGGCGATTGAGTTTAATTTTCGCCTTGTTGATAGTTCAAGAAAGTCCTTGGAAA
PH01000828	157153	157173	+
TTCCACAGCTTTCTTGAACTG 	1	21
> PH01000828:157153- 157173
TTCCACAGCTTTCTTGAACTGCATCTGCAATGGATATTAGTACTTTTCTGGCTTCTACTTGCAATAGTGCAGTTCAATAAAGTTGTGGGAAAT
PH01001240	25286	25306	+
TTCCACAGCTTTCTTGAACTG 	1	21
> PH01001240:25286-25306
TTCCACAGCTTTCTTGAACTGCATCTGTAATGGACAGCAGTACTCTTCTGGCTTCTACTTGCAAGAGTGCAGTTCAATAAAGCTGTGGGAA

## Slide 46
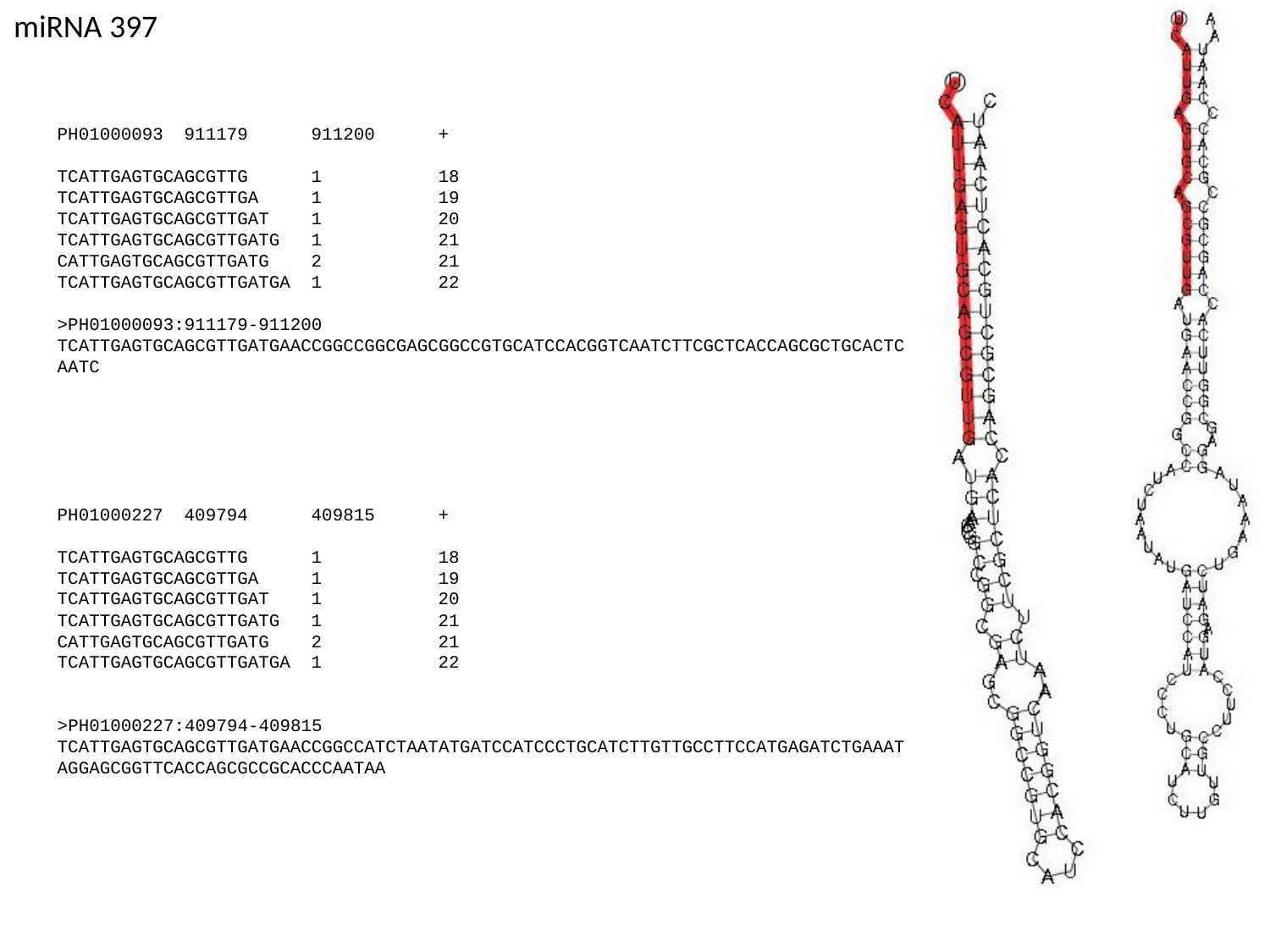

miRNA 397
PH01000093	911179	911200	+
TCATTGAGTGCAGCGTTG 	1	18
TCATTGAGTGCAGCGTTGA 	1	19
TCATTGAGTGCAGCGTTGAT 	1	20
TCATTGAGTGCAGCGTTGATG 	1	21
CATTGAGTGCAGCGTTGATG 	2	21
TCATTGAGTGCAGCGTTGATGA 	1	22
>PH01000093:911179-911200
TCATTGAGTGCAGCGTTGATGAACCGGCCGGCGAGCGGCCGTGCATCCACGGTCAATCTTCGCTCACCAGCGCTGCACTCAATC
PH01000227	409794	409815	+
TCATTGAGTGCAGCGTTG 	1	18
TCATTGAGTGCAGCGTTGA 	1	19
TCATTGAGTGCAGCGTTGAT 	1	20
TCATTGAGTGCAGCGTTGATG 	1	21
CATTGAGTGCAGCGTTGATG 	2	21
TCATTGAGTGCAGCGTTGATGA 	1	22
>PH01000227:409794-409815
TCATTGAGTGCAGCGTTGATGAACCGGCCATCTAATATGATCCATCCCTGCATCTTGTTGCCTTCCATGAGATCTGAAATAGGAGCGGTTCACCAGCGCCGCACCCAATAA

## Slide 47
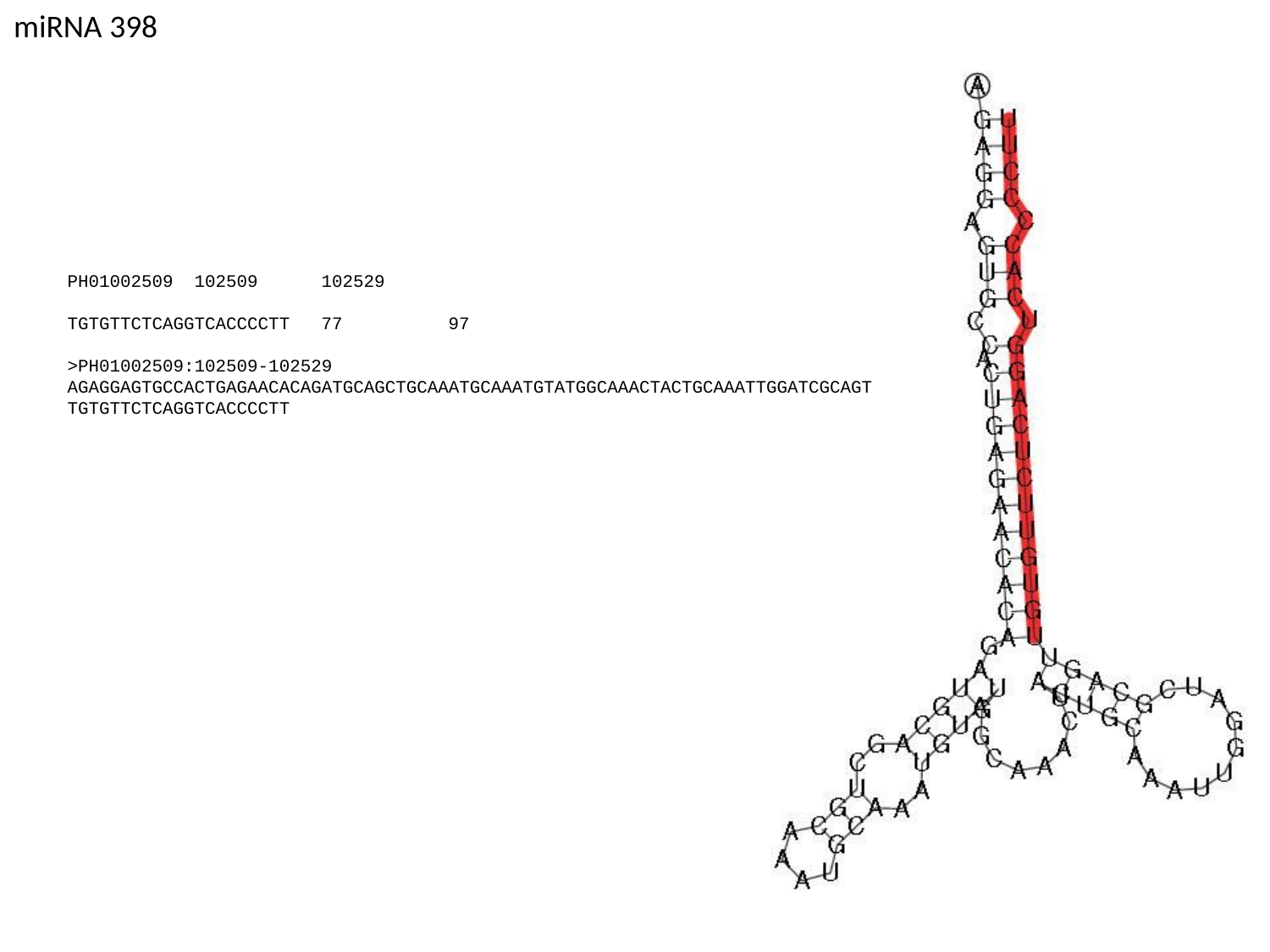

miRNA 398
PH01002509	102509	102529
TGTGTTCTCAGGTCACCCCTT 	77	97
>PH01002509:102509-102529
AGAGGAGTGCCACTGAGAACACAGATGCAGCTGCAAATGCAAATGTATGGCAAACTACTGCAAATTGGATCGCAGTTGTGTTCTCAGGTCACCCCTT

## Slide 48
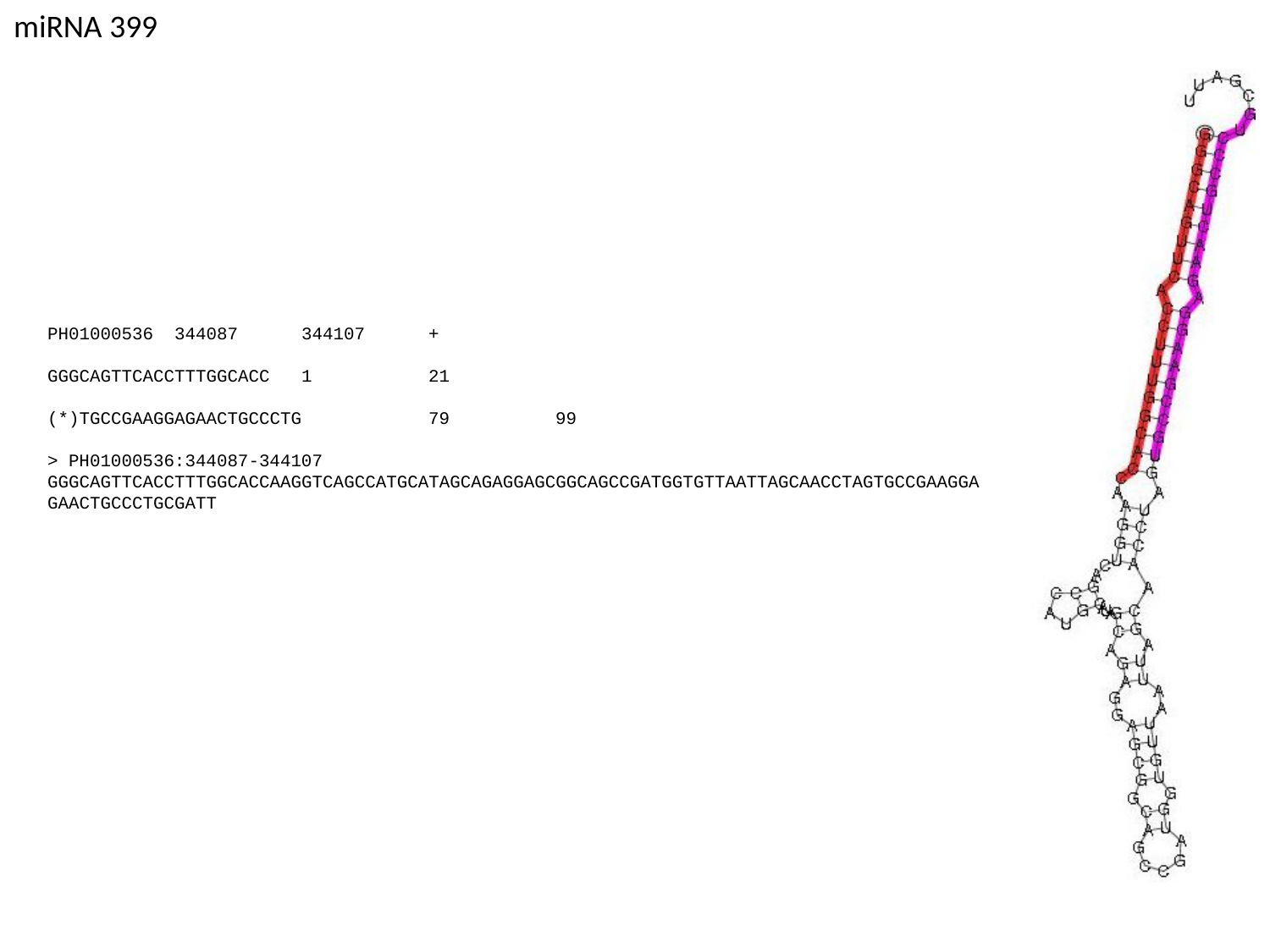

miRNA 399
PH01000536	344087	344107	+
GGGCAGTTCACCTTTGGCACC 	1	21
(*)TGCCGAAGGAGAACTGCCCTG 	79	99
> PH01000536:344087-344107
GGGCAGTTCACCTTTGGCACCAAGGTCAGCCATGCATAGCAGAGGAGCGGCAGCCGATGGTGTTAATTAGCAACCTAGTGCCGAAGGAGAACTGCCCTGCGATT

## Slide 49
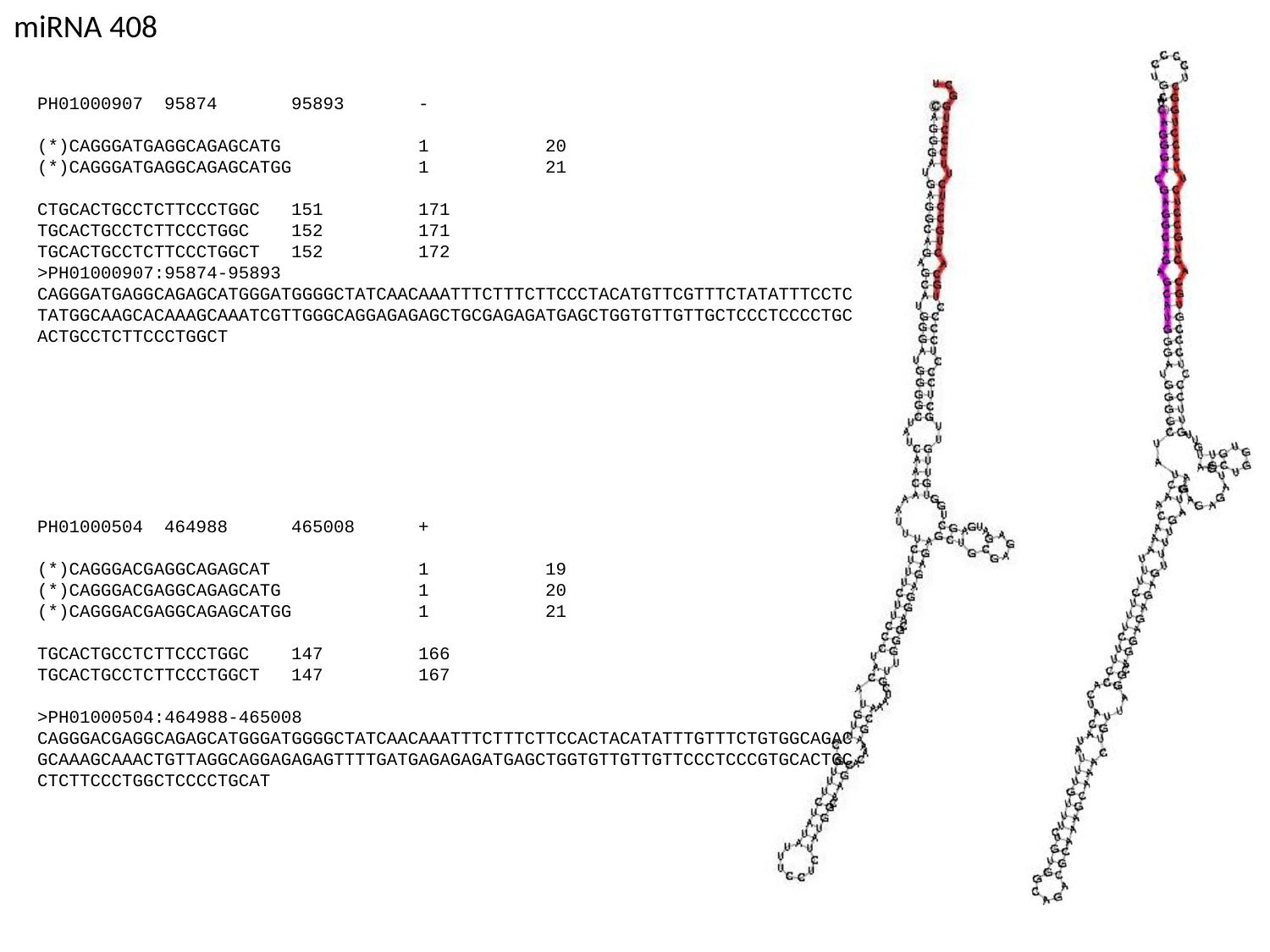

miRNA 408
PH01000907	95874	95893	-
(*)CAGGGATGAGGCAGAGCATG 	1	20
(*)CAGGGATGAGGCAGAGCATGG 	1	21
CTGCACTGCCTCTTCCCTGGC 	151	171
TGCACTGCCTCTTCCCTGGC 	152	171
TGCACTGCCTCTTCCCTGGCT 	152	172
>PH01000907:95874-95893
CAGGGATGAGGCAGAGCATGGGATGGGGCTATCAACAAATTTCTTTCTTCCCTACATGTTCGTTTCTATATTTCCTCTATGGCAAGCACAAAGCAAATCGTTGGGCAGGAGAGAGCTGCGAGAGATGAGCTGGTGTTGTTGCTCCCTCCCCTGCACTGCCTCTTCCCTGGCT
PH01000504	464988	465008	+
(*)CAGGGACGAGGCAGAGCAT 		1	19
(*)CAGGGACGAGGCAGAGCATG 	1	20
(*)CAGGGACGAGGCAGAGCATGG 	1	21
TGCACTGCCTCTTCCCTGGC 	147	166
TGCACTGCCTCTTCCCTGGCT 	147	167
>PH01000504:464988-465008
CAGGGACGAGGCAGAGCATGGGATGGGGCTATCAACAAATTTCTTTCTTCCACTACATATTTGTTTCTGTGGCAGACGCAAAGCAAACTGTTAGGCAGGAGAGAGTTTTGATGAGAGAGATGAGCTGGTGTTGTTGTTCCCTCCCGTGCACTGCCTCTTCCCTGGCTCCCCTGCAT

## Slide 50
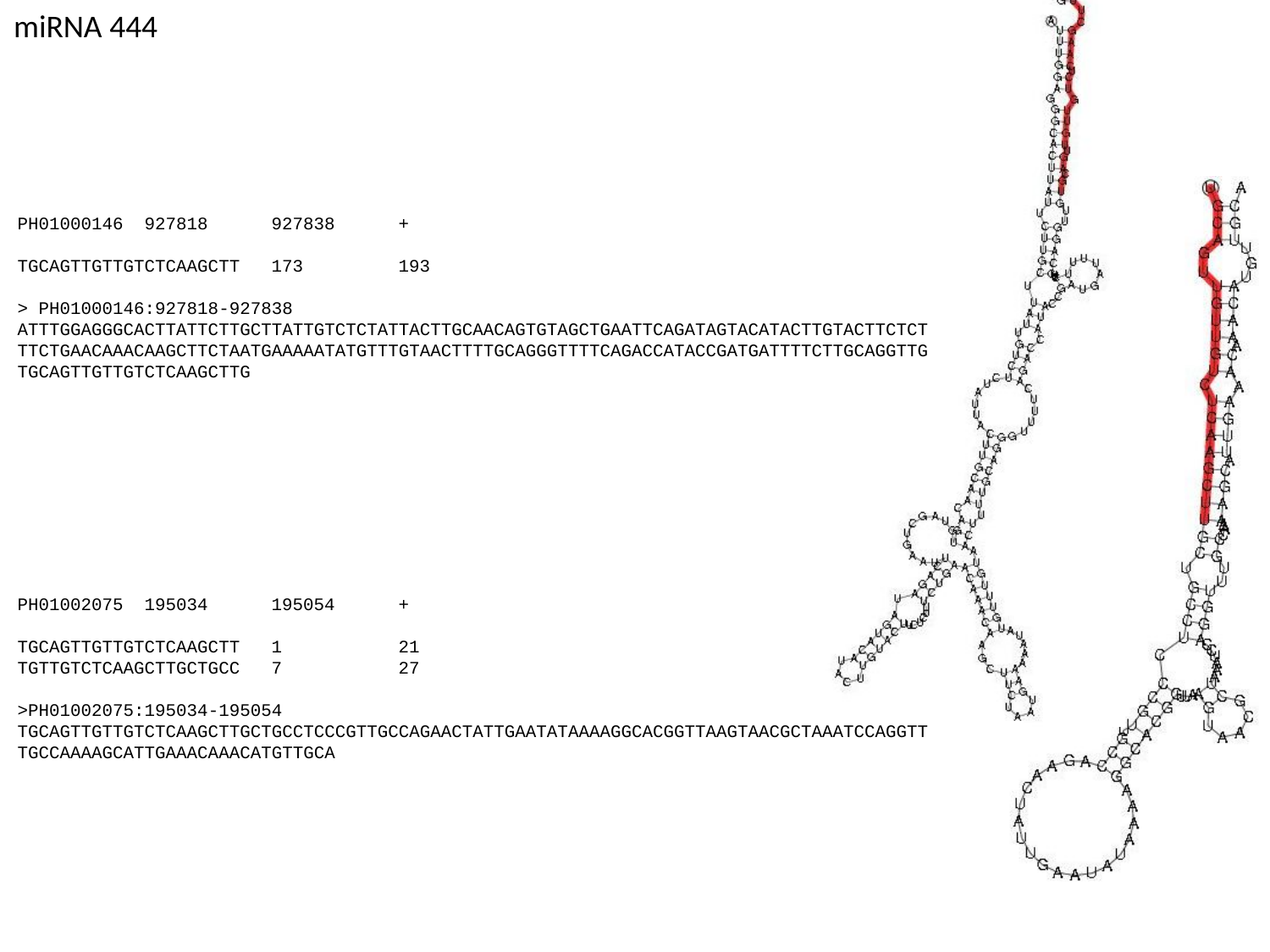

miRNA 444
PH01000146	927818	927838	+
TGCAGTTGTTGTCTCAAGCTT 	173	193
> PH01000146:927818-927838
ATTTGGAGGGCACTTATTCTTGCTTATTGTCTCTATTACTTGCAACAGTGTAGCTGAATTCAGATAGTACATACTTGTACTTCTCTTTCTGAACAAACAAGCTTCTAATGAAAAATATGTTTGTAACTTTTGCAGGGTTTTCAGACCATACCGATGATTTTCTTGCAGGTTGTGCAGTTGTTGTCTCAAGCTTG
PH01002075	195034	195054	+
TGCAGTTGTTGTCTCAAGCTT 	1	21
TGTTGTCTCAAGCTTGCTGCC 	7	27
>PH01002075:195034-195054
TGCAGTTGTTGTCTCAAGCTTGCTGCCTCCCGTTGCCAGAACTATTGAATATAAAAGGCACGGTTAAGTAACGCTAAATCCAGGTTTGCCAAAAGCATTGAAACAAACATGTTGCA

## Slide 51
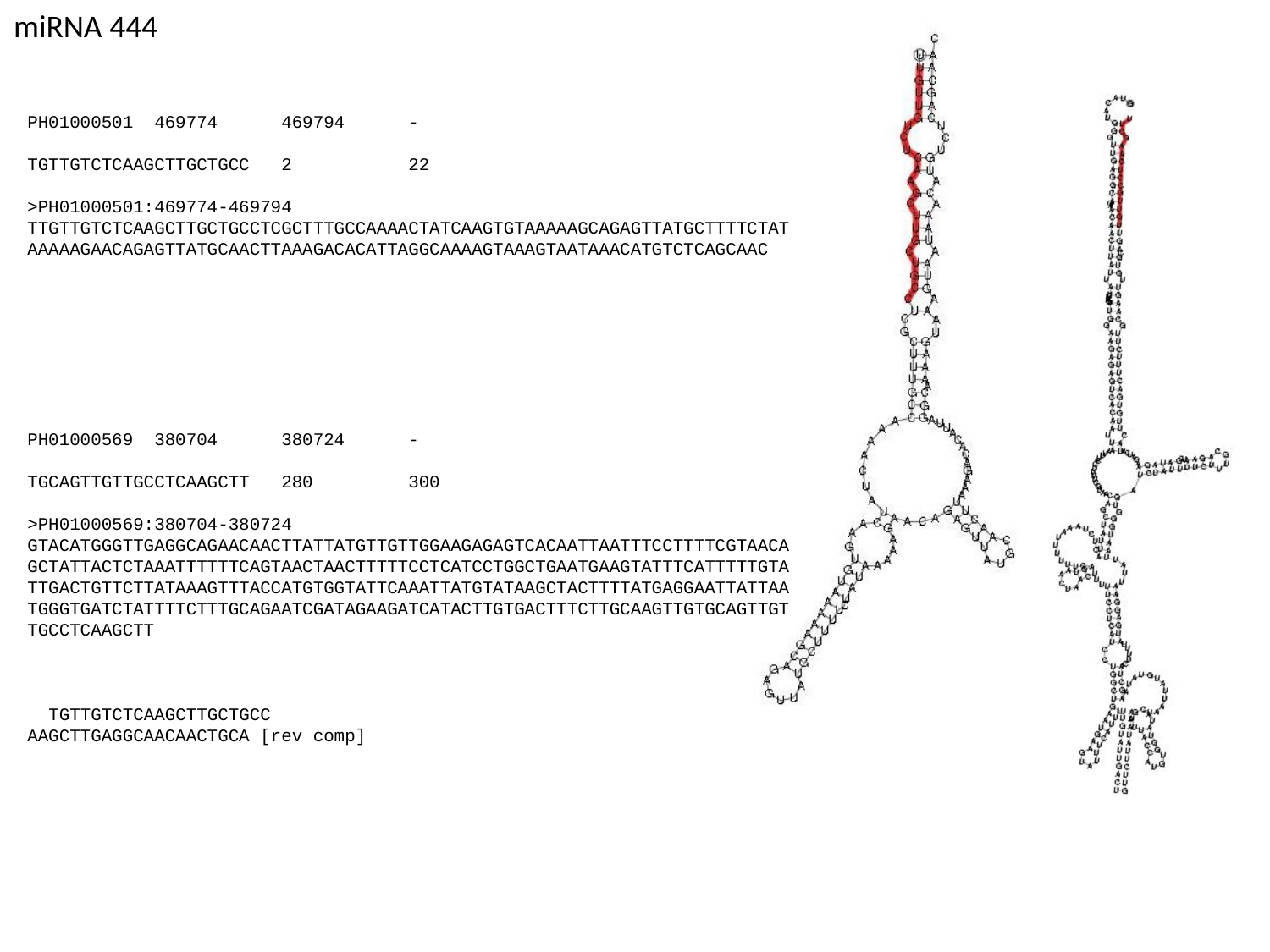

miRNA 444
PH01000501	469774	469794	-
TGTTGTCTCAAGCTTGCTGCC 	2	22
>PH01000501:469774-469794
TTGTTGTCTCAAGCTTGCTGCCTCGCTTTGCCAAAACTATCAAGTGTAAAAAGCAGAGTTATGCTTTTCTATAAAAAGAACAGAGTTATGCAACTTAAAGACACATTAGGCAAAAGTAAAGTAATAAACATGTCTCAGCAAC
PH01000569	380704	380724	-
TGCAGTTGTTGCCTCAAGCTT 	280	300
>PH01000569:380704-380724
GTACATGGGTTGAGGCAGAACAACTTATTATGTTGTTGGAAGAGAGTCACAATTAATTTCCTTTTCGTAACAGCTATTACTCTAAATTTTTTCAGTAACTAACTTTTTCCTCATCCTGGCTGAATGAAGTATTTCATTTTTGTATTGACTGTTCTTATAAAGTTTACCATGTGGTATTCAAATTATGTATAAGCTACTTTTATGAGGAATTATTAATGGGTGATCTATTTTCTTTGCAGAATCGATAGAAGATCATACTTGTGACTTTCTTGCAAGTTGTGCAGTTGTTGCCTCAAGCTT
 TGTTGTCTCAAGCTTGCTGCC
AAGCTTGAGGCAACAACTGCA [rev comp]

## Slide 52
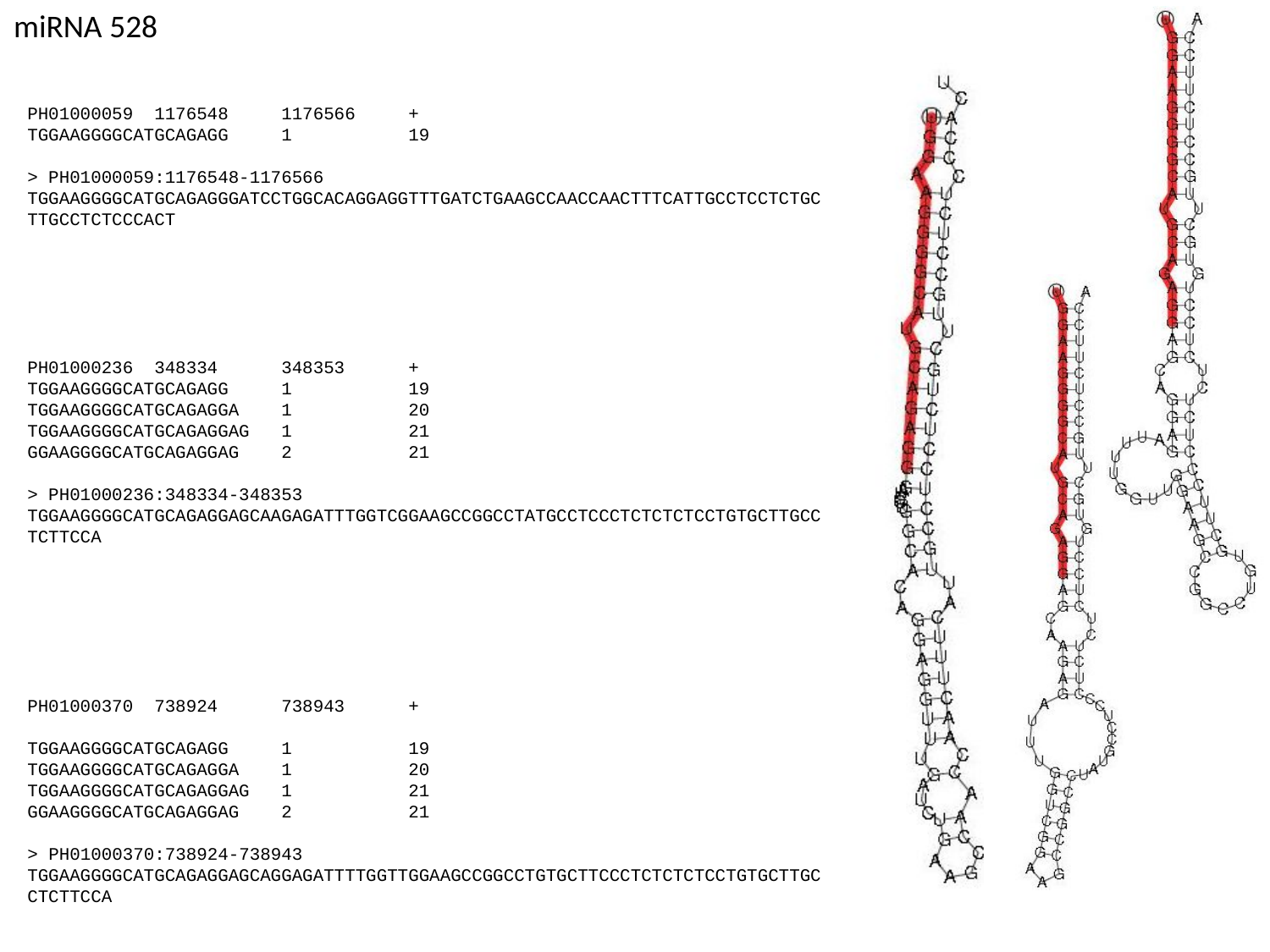

miRNA 528
PH01000059	1176548	1176566	+
TGGAAGGGGCATGCAGAGG 	1	19
> PH01000059:1176548-1176566
TGGAAGGGGCATGCAGAGGGATCCTGGCACAGGAGGTTTGATCTGAAGCCAACCAACTTTCATTGCCTCCTCTGCTTGCCTCTCCCACT
PH01000236	348334	348353	+
TGGAAGGGGCATGCAGAGG 	1	19
TGGAAGGGGCATGCAGAGGA 	1	20
TGGAAGGGGCATGCAGAGGAG 	1	21
GGAAGGGGCATGCAGAGGAG 	2	21
> PH01000236:348334-348353
TGGAAGGGGCATGCAGAGGAGCAAGAGATTTGGTCGGAAGCCGGCCTATGCCTCCCTCTCTCTCCTGTGCTTGCCTCTTCCA
PH01000370	738924	738943	+
TGGAAGGGGCATGCAGAGG 	1	19
TGGAAGGGGCATGCAGAGGA 	1	20
TGGAAGGGGCATGCAGAGGAG 	1	21
GGAAGGGGCATGCAGAGGAG 	2	21
> PH01000370:738924-738943
TGGAAGGGGCATGCAGAGGAGCAGGAGATTTTGGTTGGAAGCCGGCCTGTGCTTCCCTCTCTCTCCTGTGCTTGCCTCTTCCA

## Slide 53
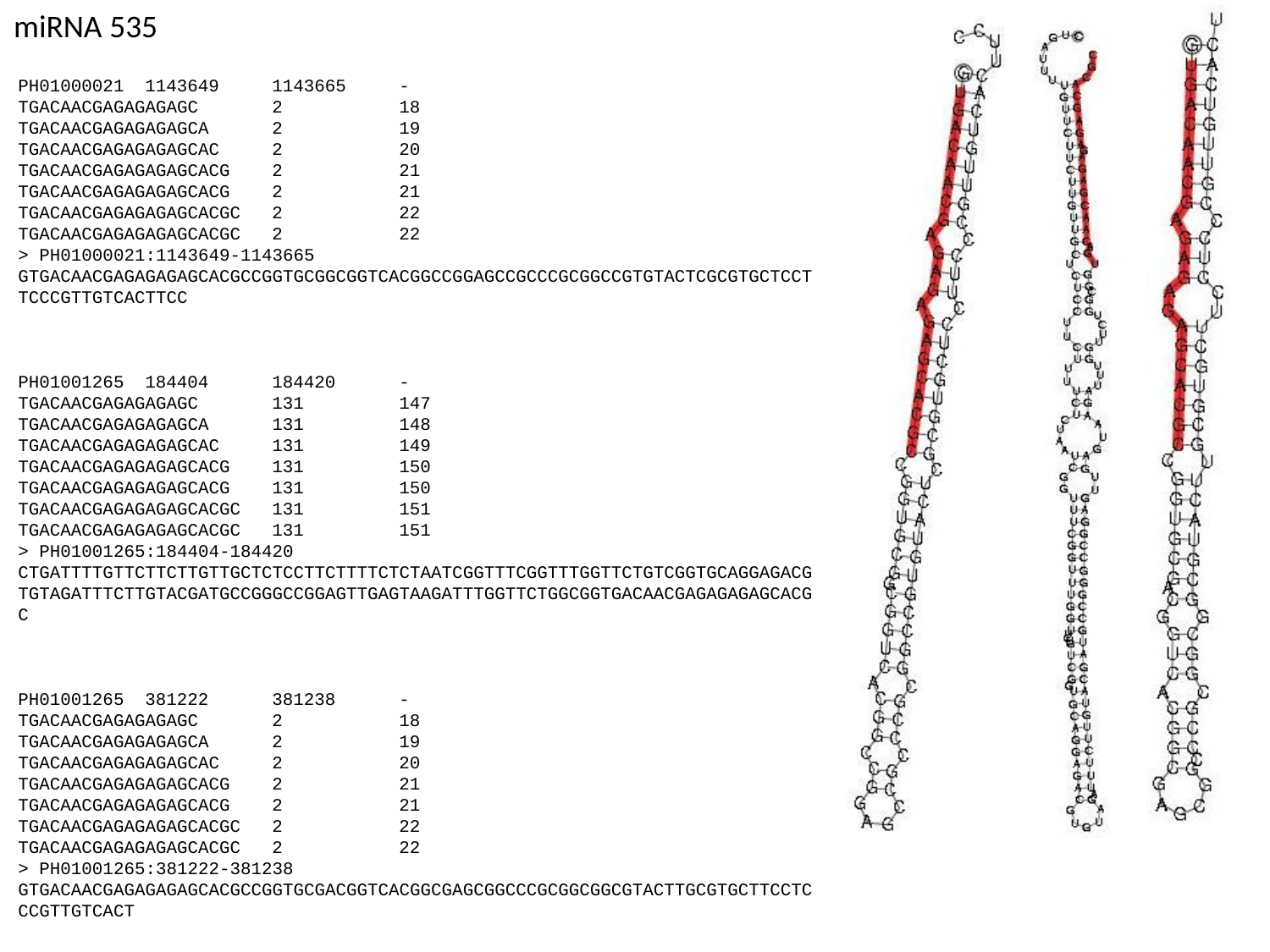

miRNA 535
PH01000021	1143649	1143665	-
TGACAACGAGAGAGAGC 	2	18
TGACAACGAGAGAGAGCA 	2	19
TGACAACGAGAGAGAGCAC 	2	20
TGACAACGAGAGAGAGCACG 	2	21
TGACAACGAGAGAGAGCACG 	2	21
TGACAACGAGAGAGAGCACGC 	2	22
TGACAACGAGAGAGAGCACGC 	2	22
> PH01000021:1143649-1143665
GTGACAACGAGAGAGAGCACGCCGGTGCGGCGGTCACGGCCGGAGCCGCCCGCGGCCGTGTACTCGCGTGCTCCTTCCCGTTGTCACTTCC
PH01001265	184404	184420	-
TGACAACGAGAGAGAGC 	131	147
TGACAACGAGAGAGAGCA 	131	148
TGACAACGAGAGAGAGCAC 	131	149
TGACAACGAGAGAGAGCACG 	131	150
TGACAACGAGAGAGAGCACG 	131	150
TGACAACGAGAGAGAGCACGC 	131	151
TGACAACGAGAGAGAGCACGC 	131	151
> PH01001265:184404-184420
CTGATTTTGTTCTTCTTGTTGCTCTCCTTCTTTTCTCTAATCGGTTTCGGTTTGGTTCTGTCGGTGCAGGAGACGTGTAGATTTCTTGTACGATGCCGGGCCGGAGTTGAGTAAGATTTGGTTCTGGCGGTGACAACGAGAGAGAGCACGC
PH01001265	381222	381238	-
TGACAACGAGAGAGAGC 	2	18
TGACAACGAGAGAGAGCA 	2	19
TGACAACGAGAGAGAGCAC 	2	20
TGACAACGAGAGAGAGCACG 	2	21
TGACAACGAGAGAGAGCACG 	2	21
TGACAACGAGAGAGAGCACGC 	2	22
TGACAACGAGAGAGAGCACGC 	2	22
> PH01001265:381222-381238
GTGACAACGAGAGAGAGCACGCCGGTGCGACGGTCACGGCGAGCGGCCCGCGGCGGCGTACTTGCGTGCTTCCTCCCGTTGTCACT

## Slide 54
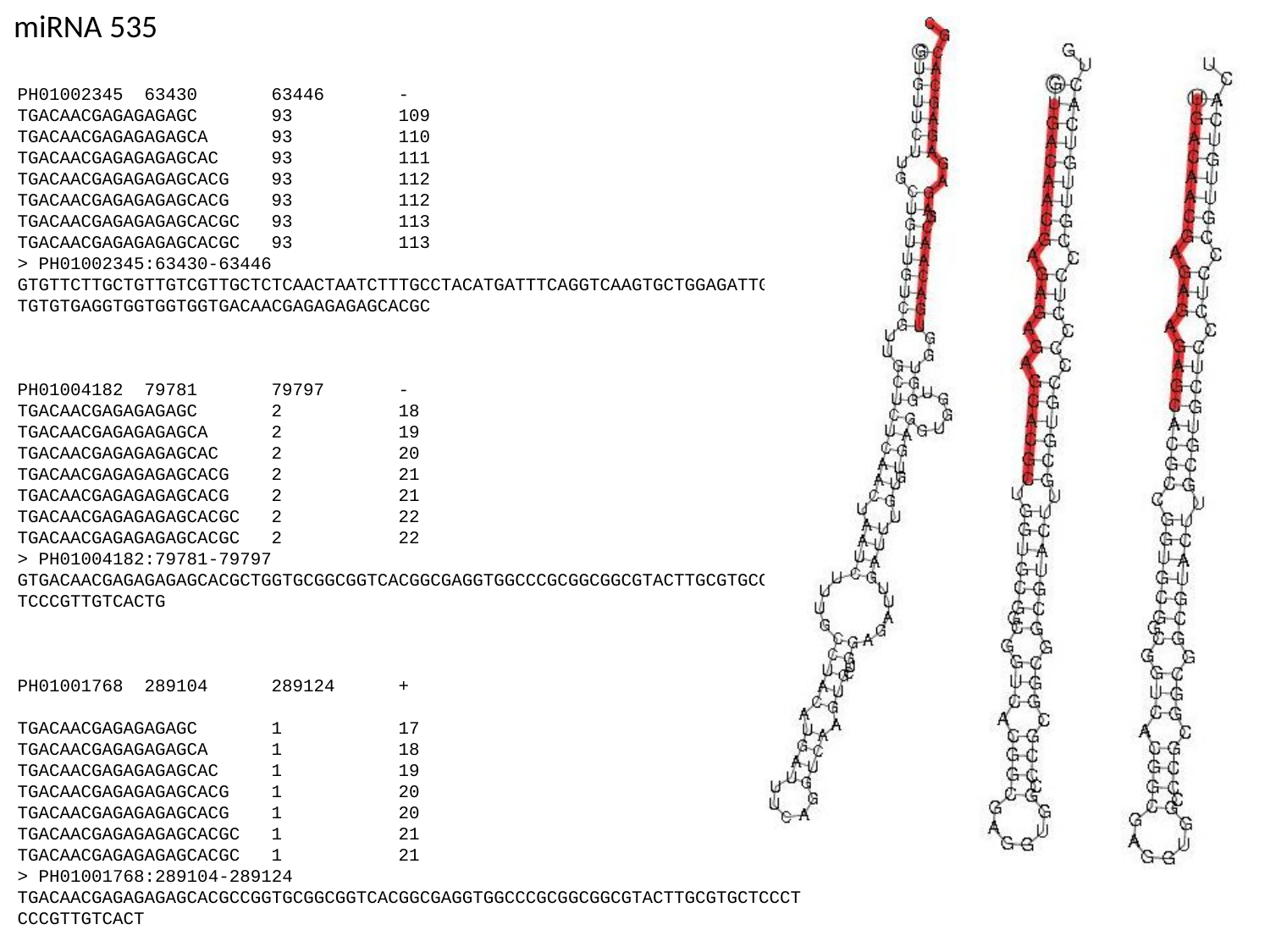

miRNA 535
PH01002345	63430	63446	-
TGACAACGAGAGAGAGC 	93	109
TGACAACGAGAGAGAGCA 	93	110
TGACAACGAGAGAGAGCAC 	93	111
TGACAACGAGAGAGAGCACG 	93	112
TGACAACGAGAGAGAGCACG 	93	112
TGACAACGAGAGAGAGCACGC 	93	113
TGACAACGAGAGAGAGCACGC 	93	113
> PH01002345:63430-63446
GTGTTCTTGCTGTTGTCGTTGCTCTCAACTAATCTTTGCCTACATGATTTCAGGTCAAGTGCTGGAGATTGATTTGTGTGAGGTGGTGGTGGTGACAACGAGAGAGAGCACGC
PH01004182	79781	79797	-
TGACAACGAGAGAGAGC 	2	18
TGACAACGAGAGAGAGCA 	2	19
TGACAACGAGAGAGAGCAC 	2	20
TGACAACGAGAGAGAGCACG 	2	21
TGACAACGAGAGAGAGCACG 	2	21
TGACAACGAGAGAGAGCACGC 	2	22
TGACAACGAGAGAGAGCACGC 	2	22
> PH01004182:79781-79797
GTGACAACGAGAGAGAGCACGCTGGTGCGGCGGTCACGGCGAGGTGGCCCGCGGCGGCGTACTTGCGTGCCCCCTCCCGTTGTCACTG
PH01001768	289104	289124	+
TGACAACGAGAGAGAGC 	1	17
TGACAACGAGAGAGAGCA 	1	18
TGACAACGAGAGAGAGCAC 	1	19
TGACAACGAGAGAGAGCACG 	1	20
TGACAACGAGAGAGAGCACG 	1	20
TGACAACGAGAGAGAGCACGC 	1	21
TGACAACGAGAGAGAGCACGC 	1	21
> PH01001768:289104-289124
TGACAACGAGAGAGAGCACGCCGGTGCGGCGGTCACGGCGAGGTGGCCCGCGGCGGCGTACTTGCGTGCTCCCTCCCGTTGTCACT

## Slide 55
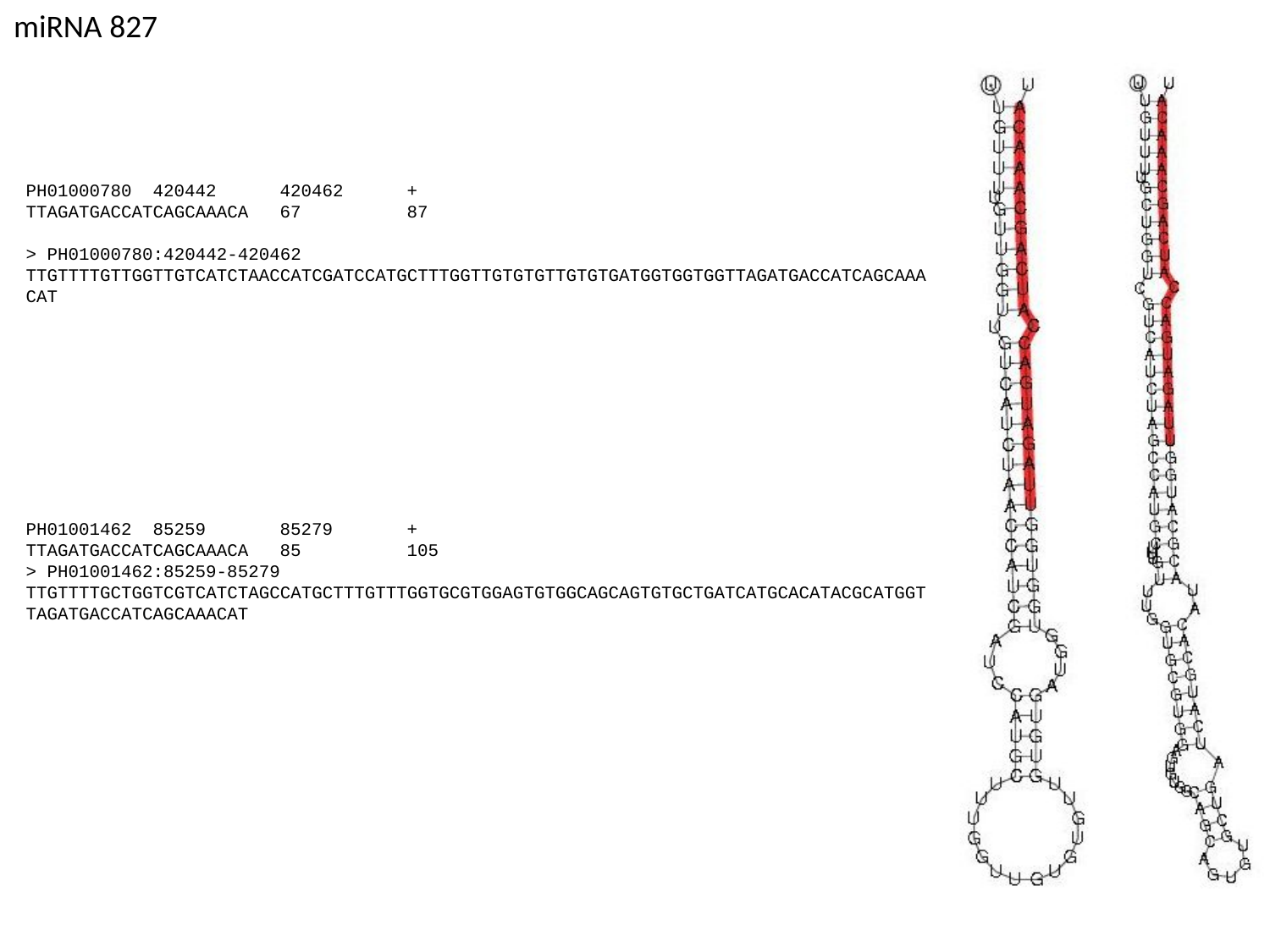

miRNA 827
PH01000780	420442	420462	+
TTAGATGACCATCAGCAAACA 	67	87
> PH01000780:420442-420462
TTGTTTTGTTGGTTGTCATCTAACCATCGATCCATGCTTTGGTTGTGTGTTGTGTGATGGTGGTGGTTAGATGACCATCAGCAAACAT
PH01001462	85259	85279	+
TTAGATGACCATCAGCAAACA 	85	105
> PH01001462:85259-85279
TTGTTTTGCTGGTCGTCATCTAGCCATGCTTTGTTTGGTGCGTGGAGTGTGGCAGCAGTGTGCTGATCATGCACATACGCATGGTTAGATGACCATCAGCAAACAT

## Slide 56
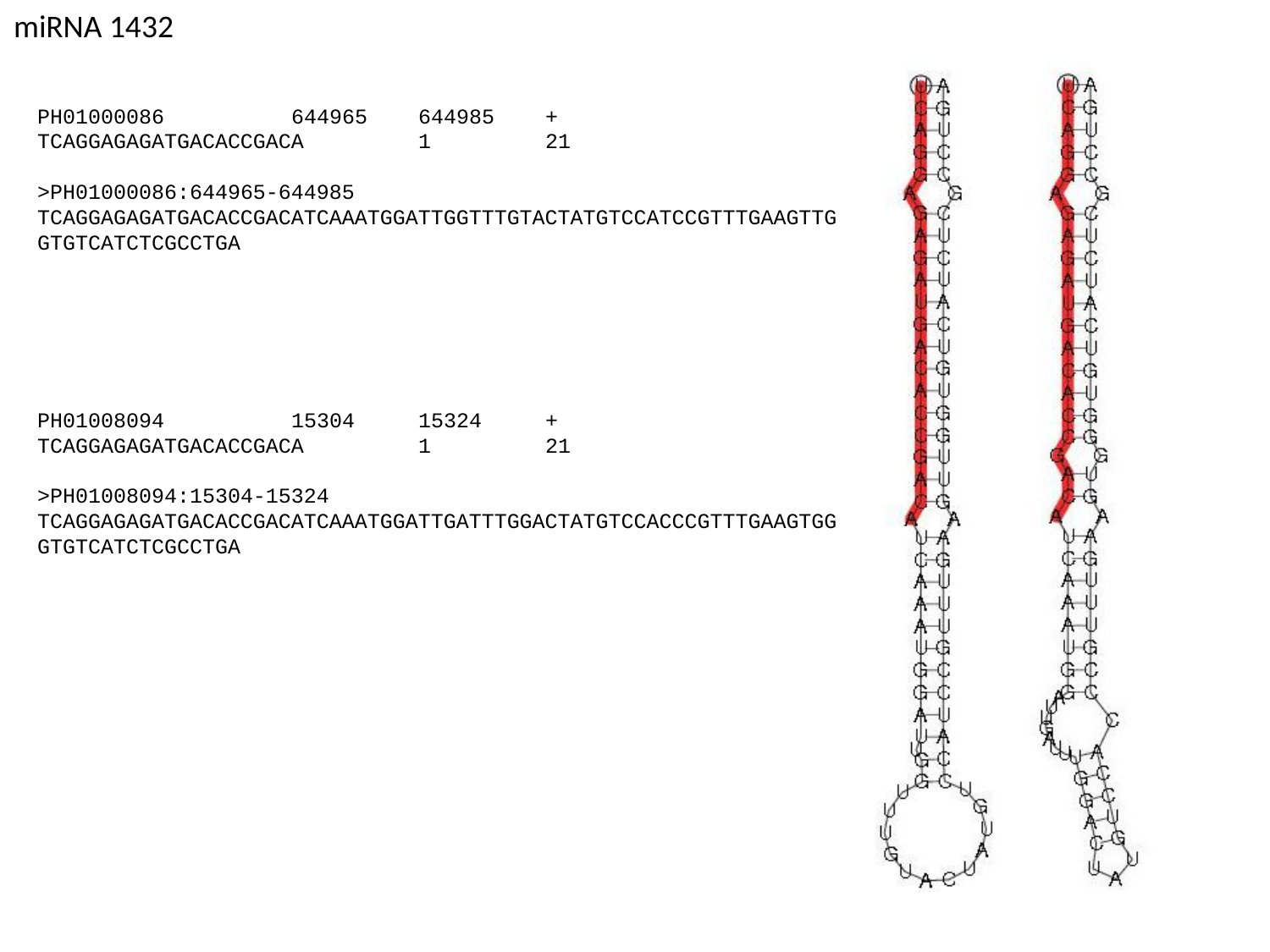

miRNA 1432
PH01000086	644965	644985	+
TCAGGAGAGATGACACCGACA	1	21
>PH01000086:644965-644985
TCAGGAGAGATGACACCGACATCAAATGGATTGGTTTGTACTATGTCCATCCGTTTGAAGTTGGTGTCATCTCGCCTGA
PH01008094	15304	15324	+
TCAGGAGAGATGACACCGACA	1	21
>PH01008094:15304-15324
TCAGGAGAGATGACACCGACATCAAATGGATTGATTTGGACTATGTCCACCCGTTTGAAGTGGGTGTCATCTCGCCTGA

## Slide 57
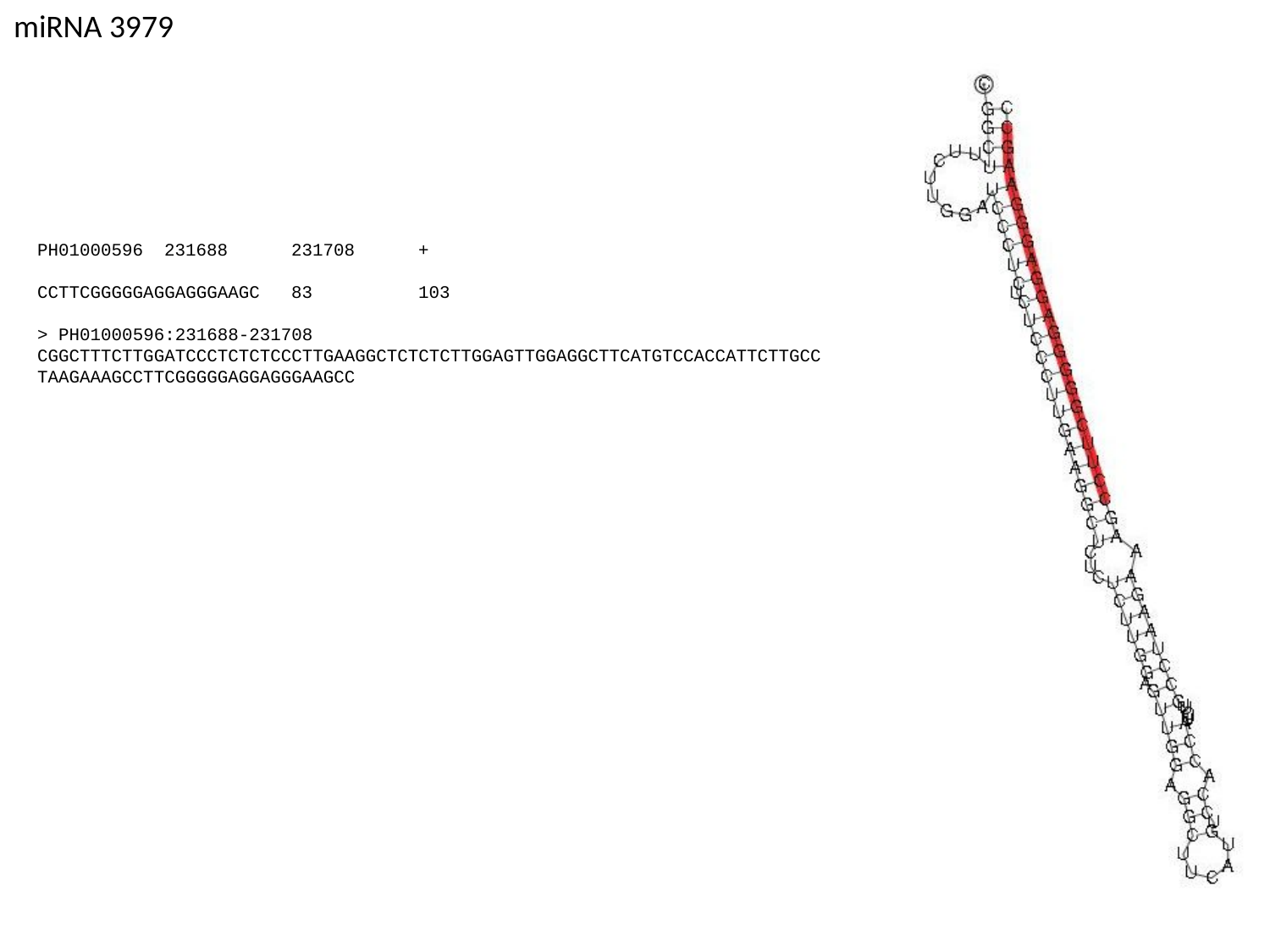

miRNA 3979
PH01000596	231688	231708	+
CCTTCGGGGGAGGAGGGAAGC 	83	103
> PH01000596:231688-231708
CGGCTTTCTTGGATCCCTCTCTCCCTTGAAGGCTCTCTCTTGGAGTTGGAGGCTTCATGTCCACCATTCTTGCCTAAGAAAGCCTTCGGGGGAGGAGGGAAGCC

## Slide 58
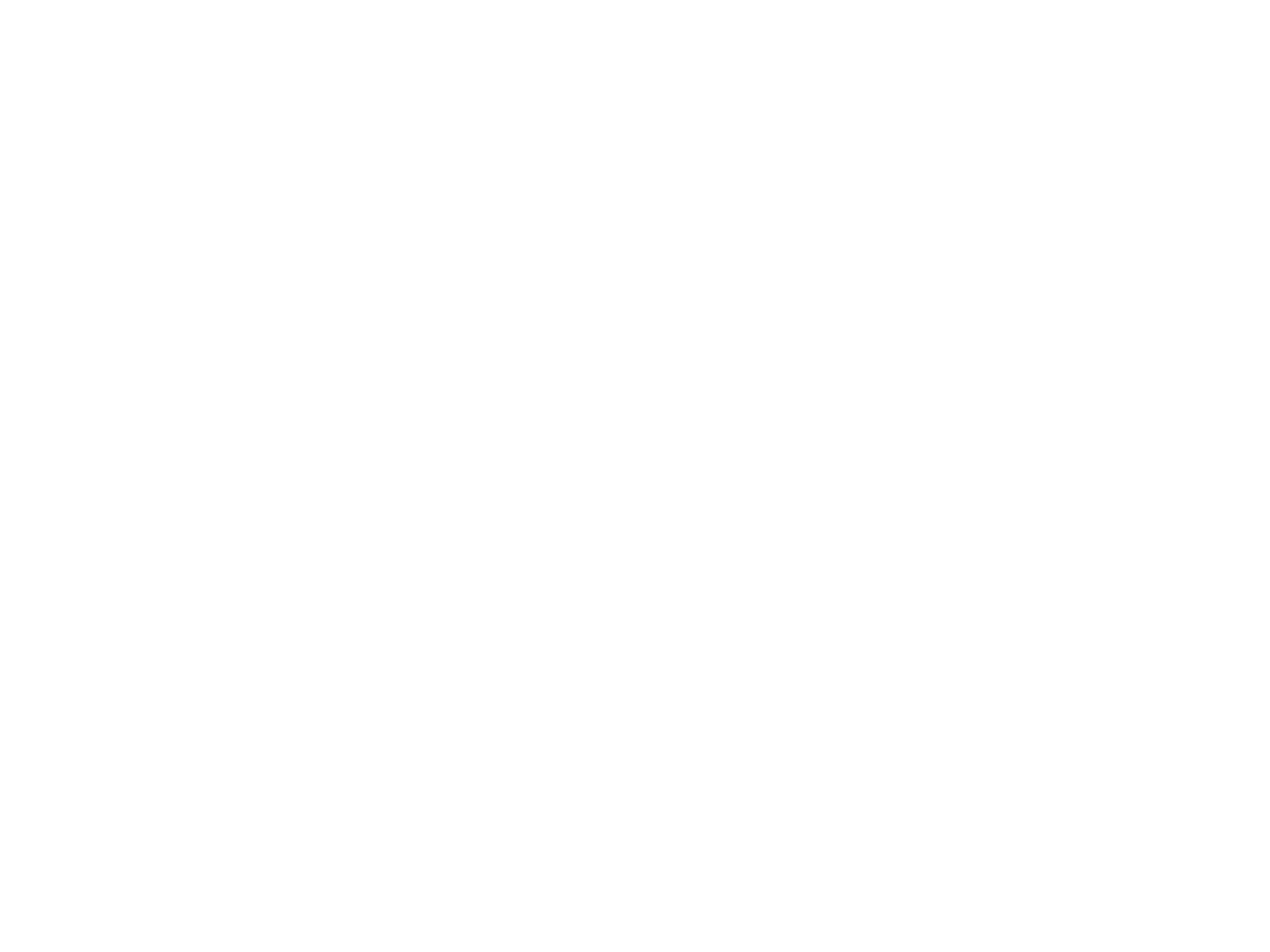

Supplement: Figure S2 — Predicted hairpin structures for all the identified known miRNAs and other general information. (PPTX) [file pone.0103590.s002.pptx]
