# Supplementary material for: Small RNA Profile in Moso Bamboo Root and Leaf Obtained by High Definition Adapters
Source: PLoS One. 2014 Jul 31;9(7):e103590. doi: 10.1371/journal.pone.0103590 (PMC4117519; doi:10.1371/journal.pone.0103590)
Supplement: Figure S3 — Predicted hairpin structures for all the predicted new miRNAs and other general information. (PPTX) [file pone.0103590.s003.pptx]

## Slide 1
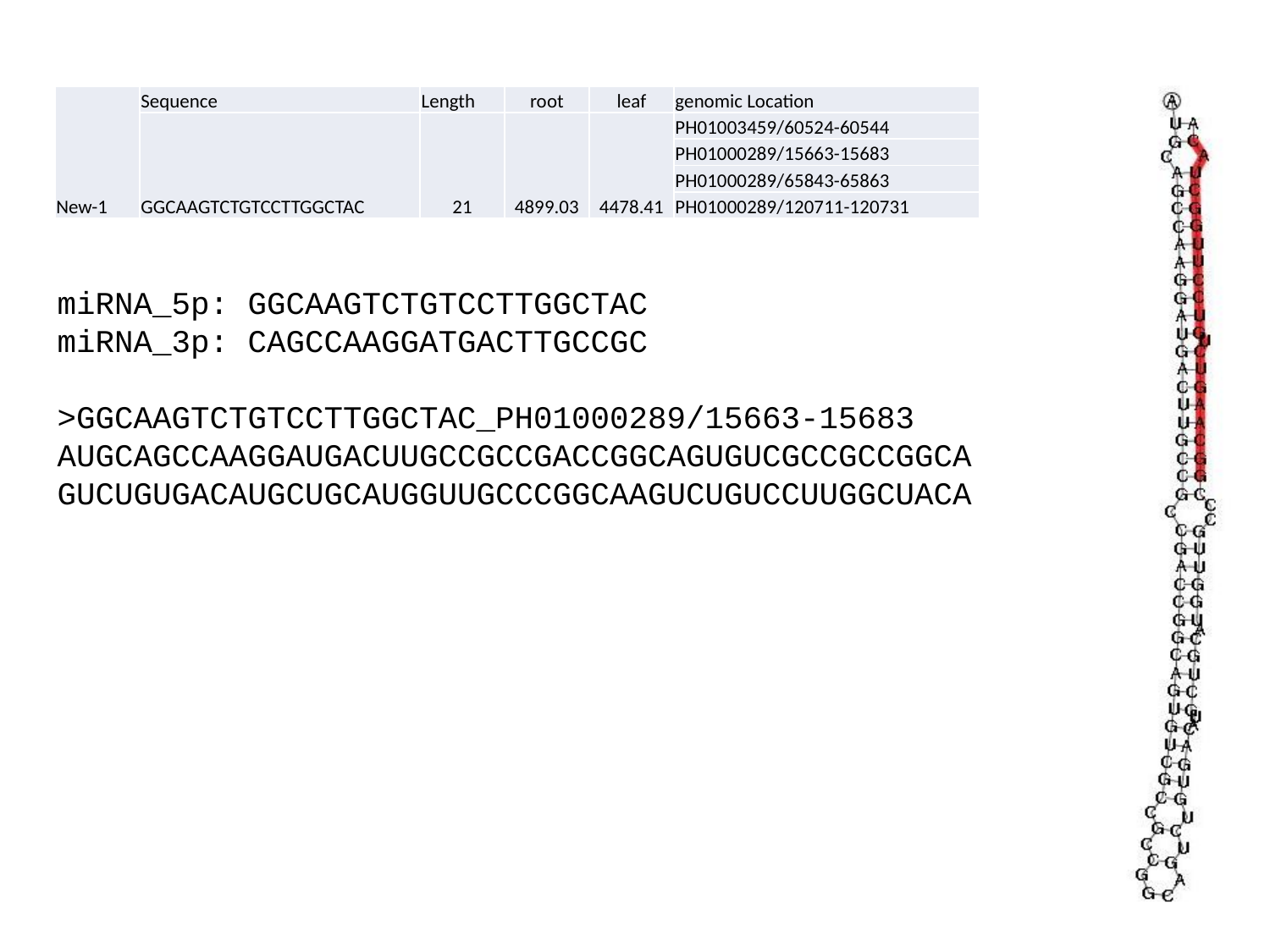

| New-1 | Sequence | Length | root | leaf | genomic Location |
| --- | --- | --- | --- | --- | --- |
| | GGCAAGTCTGTCCTTGGCTAC | 21 | 4899.03 | 4478.41 | PH01003459/60524-60544 |
| | | | | | PH01000289/15663-15683 |
| | | | | | PH01000289/65843-65863 |
| | | | | | PH01000289/120711-120731 |
miRNA_5p: GGCAAGTCTGTCCTTGGCTAC
miRNA_3p: CAGCCAAGGATGACTTGCCGC
>GGCAAGTCTGTCCTTGGCTAC_PH01000289/15663-15683
AUGCAGCCAAGGAUGACUUGCCGCCGACCGGCAGUGUCGCCGCCGGCAGUCUGUGACAUGCUGCAUGGUUGCCCGGCAAGUCUGUCCUUGGCUACA

## Slide 2
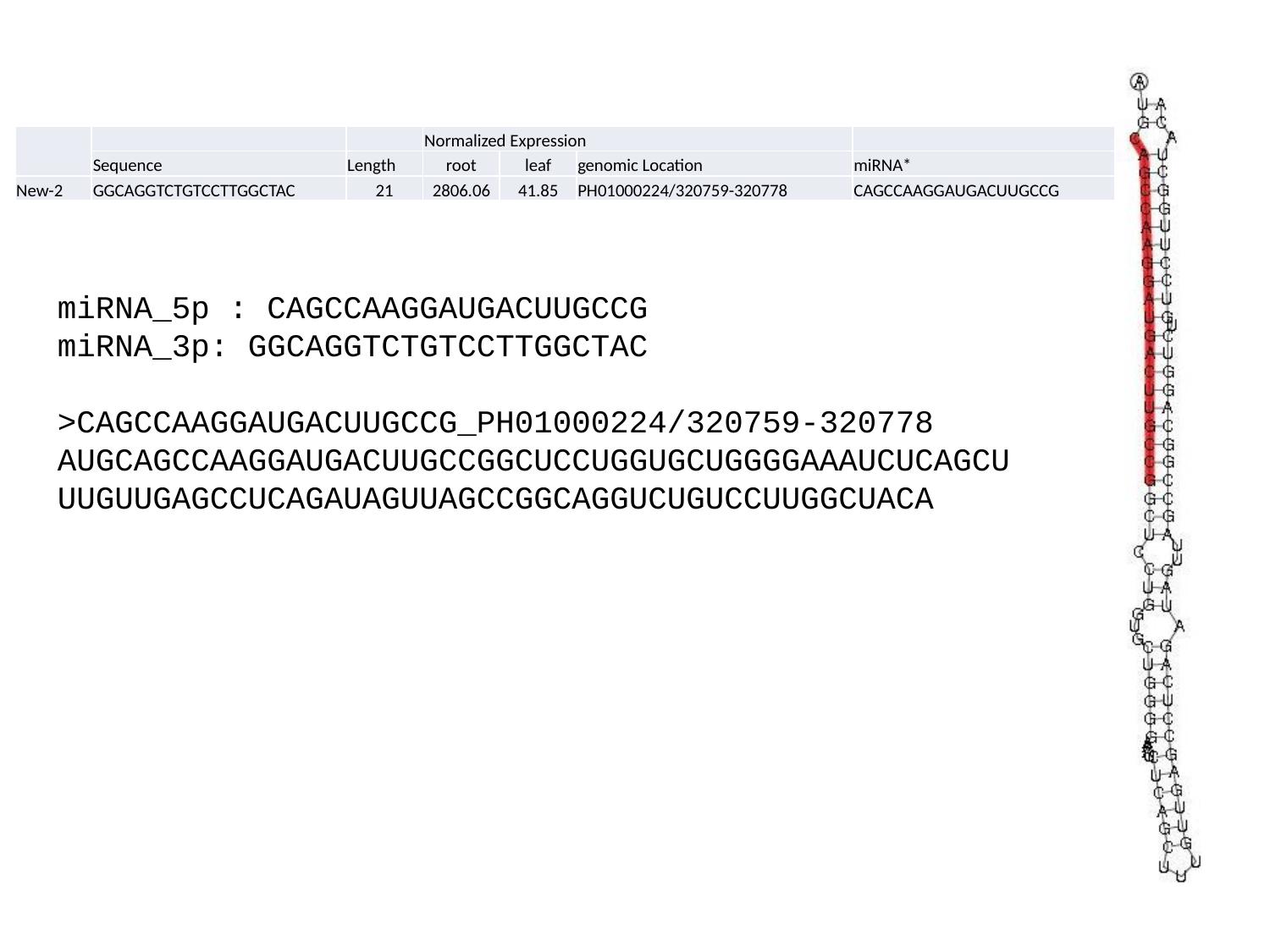

| | | | Normalized Expression | | | |
| --- | --- | --- | --- | --- | --- | --- |
| | Sequence | Length | root | leaf | genomic Location | miRNA\* |
| New-2 | GGCAGGTCTGTCCTTGGCTAC | 21 | 2806.06 | 41.85 | PH01000224/320759-320778 | CAGCCAAGGAUGACUUGCCG |
miRNA_5p : CAGCCAAGGAUGACUUGCCG
miRNA_3p: GGCAGGTCTGTCCTTGGCTAC
>CAGCCAAGGAUGACUUGCCG_PH01000224/320759-320778
AUGCAGCCAAGGAUGACUUGCCGGCUCCUGGUGCUGGGGAAAUCUCAGCUUUGUUGAGCCUCAGAUAGUUAGCCGGCAGGUCUGUCCUUGGCUACA

## Slide 3
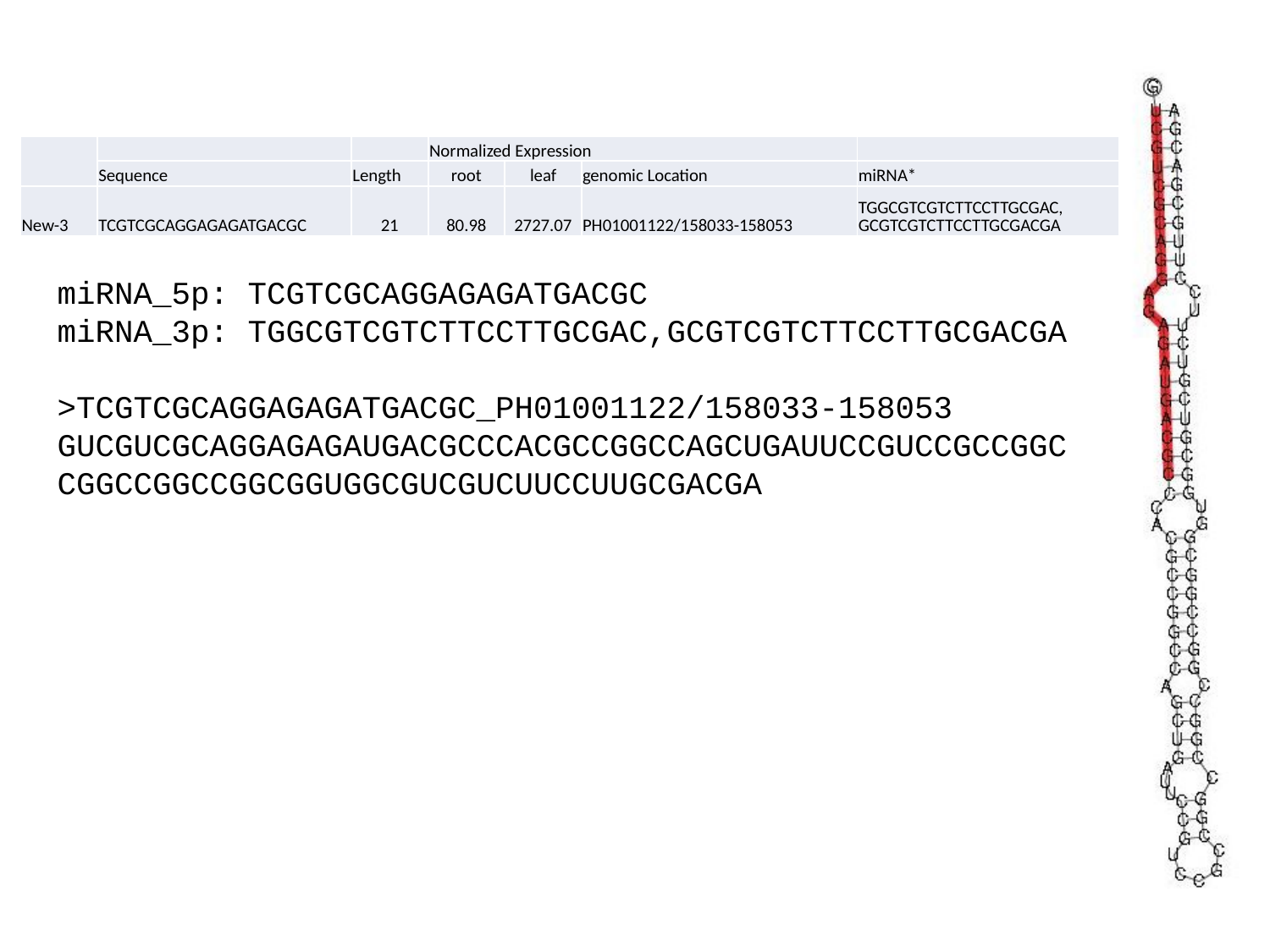

| | | | Normalized Expression | | | |
| --- | --- | --- | --- | --- | --- | --- |
| | Sequence | Length | root | leaf | genomic Location | miRNA\* |
| New-3 | TCGTCGCAGGAGAGATGACGC | 21 | 80.98 | 2727.07 | PH01001122/158033-158053 | TGGCGTCGTCTTCCTTGCGAC, GCGTCGTCTTCCTTGCGACGA |
miRNA_5p: TCGTCGCAGGAGAGATGACGC
miRNA_3p: TGGCGTCGTCTTCCTTGCGAC,GCGTCGTCTTCCTTGCGACGA
>TCGTCGCAGGAGAGATGACGC_PH01001122/158033-158053
GUCGUCGCAGGAGAGAUGACGCCCACGCCGGCCAGCUGAUUCCGUCCGCCGGCCGGCCGGCCGGCGGUGGCGUCGUCUUCCUUGCGACGA

## Slide 4
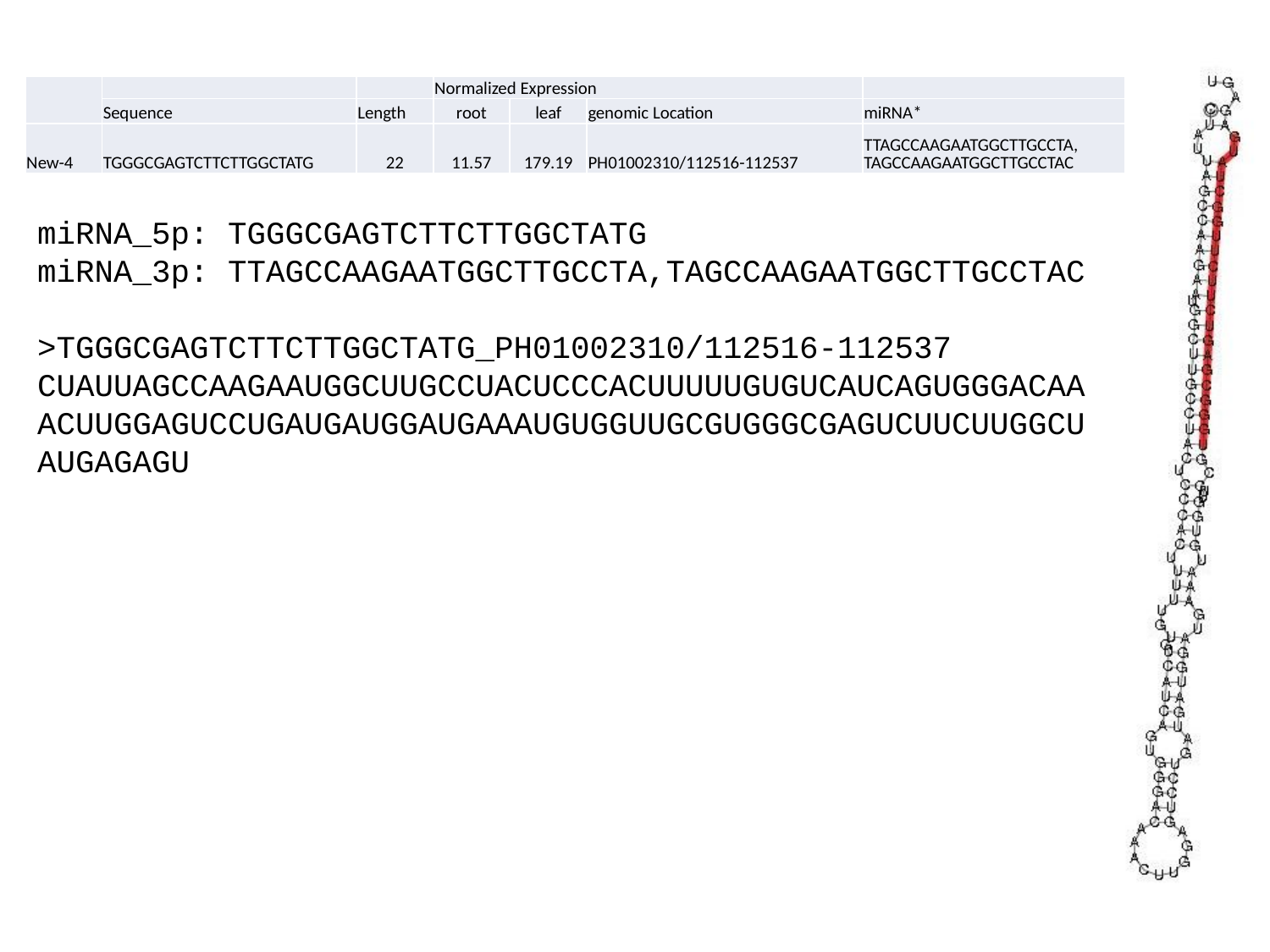

| | | | Normalized Expression | | | |
| --- | --- | --- | --- | --- | --- | --- |
| | Sequence | Length | root | leaf | genomic Location | miRNA\* |
| New-4 | TGGGCGAGTCTTCTTGGCTATG | 22 | 11.57 | 179.19 | PH01002310/112516-112537 | TTAGCCAAGAATGGCTTGCCTA, TAGCCAAGAATGGCTTGCCTAC |
miRNA_5p: TGGGCGAGTCTTCTTGGCTATG
miRNA_3p: TTAGCCAAGAATGGCTTGCCTA,TAGCCAAGAATGGCTTGCCTAC
>TGGGCGAGTCTTCTTGGCTATG_PH01002310/112516-112537
CUAUUAGCCAAGAAUGGCUUGCCUACUCCCACUUUUUGUGUCAUCAGUGGGACAAACUUGGAGUCCUGAUGAUGGAUGAAAUGUGGUUGCGUGGGCGAGUCUUCUUGGCUAUGAGAGU

## Slide 5
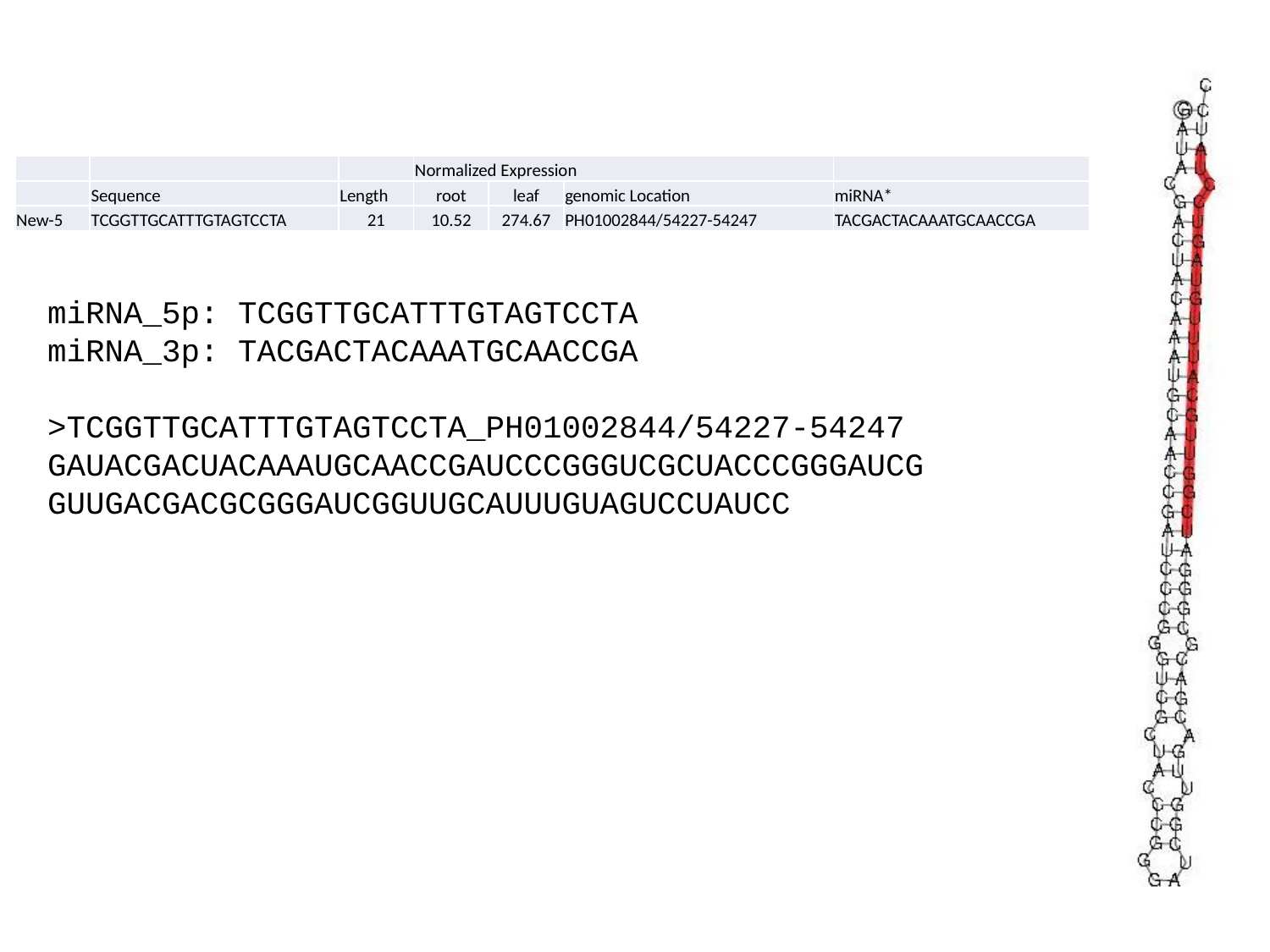

| | | | Normalized Expression | | | |
| --- | --- | --- | --- | --- | --- | --- |
| | Sequence | Length | root | leaf | genomic Location | miRNA\* |
| New-5 | TCGGTTGCATTTGTAGTCCTA | 21 | 10.52 | 274.67 | PH01002844/54227-54247 | TACGACTACAAATGCAACCGA |
miRNA_5p: TCGGTTGCATTTGTAGTCCTA
miRNA_3p: TACGACTACAAATGCAACCGA
>TCGGTTGCATTTGTAGTCCTA_PH01002844/54227-54247
GAUACGACUACAAAUGCAACCGAUCCCGGGUCGCUACCCGGGAUCGGUUGACGACGCGGGAUCGGUUGCAUUUGUAGUCCUAUCC

## Slide 6
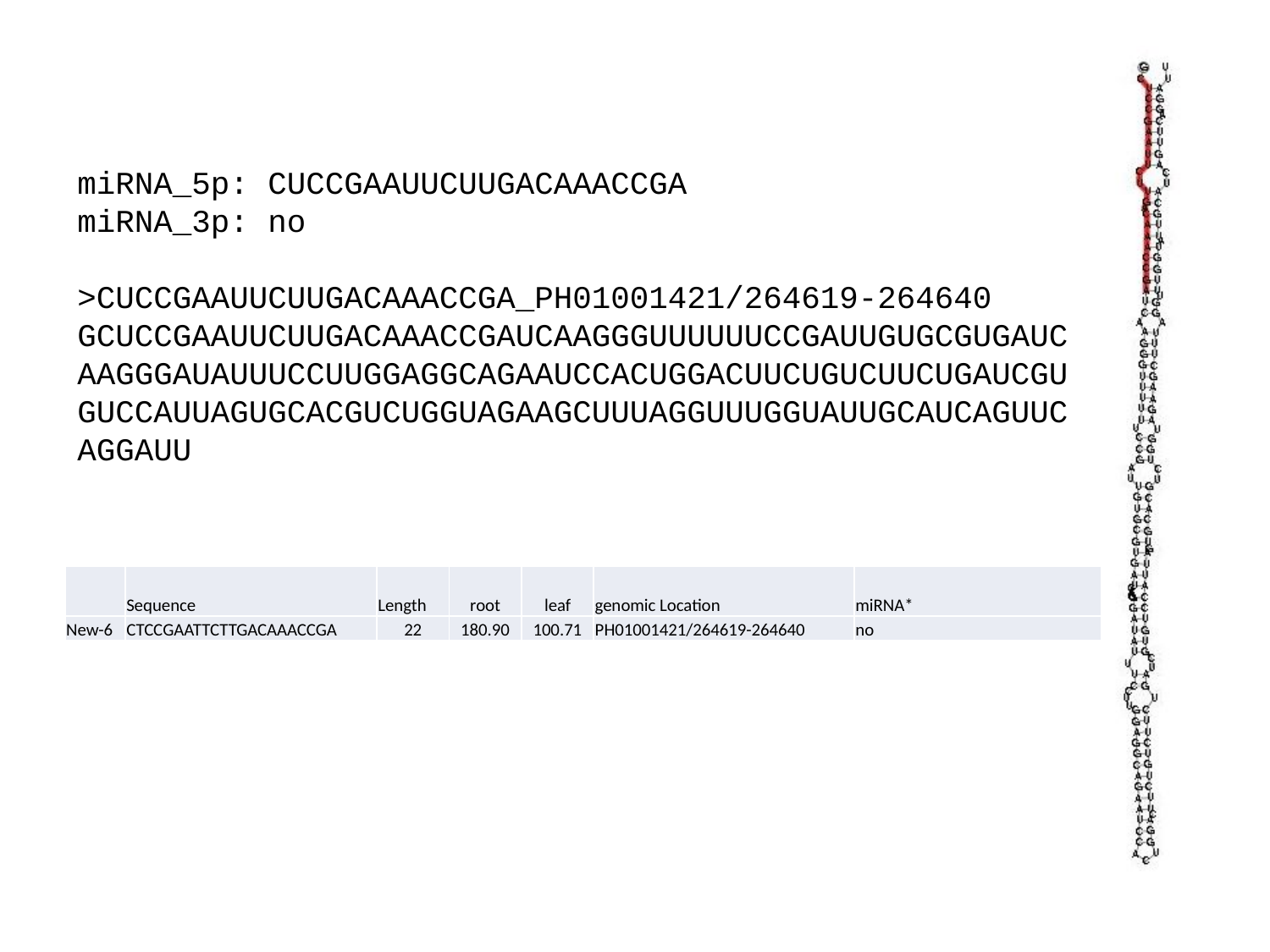

miRNA_5p: CUCCGAAUUCUUGACAAACCGA
miRNA_3p: no
>CUCCGAAUUCUUGACAAACCGA_PH01001421/264619-264640
GCUCCGAAUUCUUGACAAACCGAUCAAGGGUUUUUUCCGAUUGUGCGUGAUCAAGGGAUAUUUCCUUGGAGGCAGAAUCCACUGGACUUCUGUCUUCUGAUCGUGUCCAUUAGUGCACGUCUGGUAGAAGCUUUAGGUUUGGUAUUGCAUCAGUUCAGGAUU
| | Sequence | Length | root | leaf | genomic Location | miRNA\* |
| --- | --- | --- | --- | --- | --- | --- |
| New-6 | CTCCGAATTCTTGACAAACCGA | 22 | 180.90 | 100.71 | PH01001421/264619-264640 | no |

## Slide 7
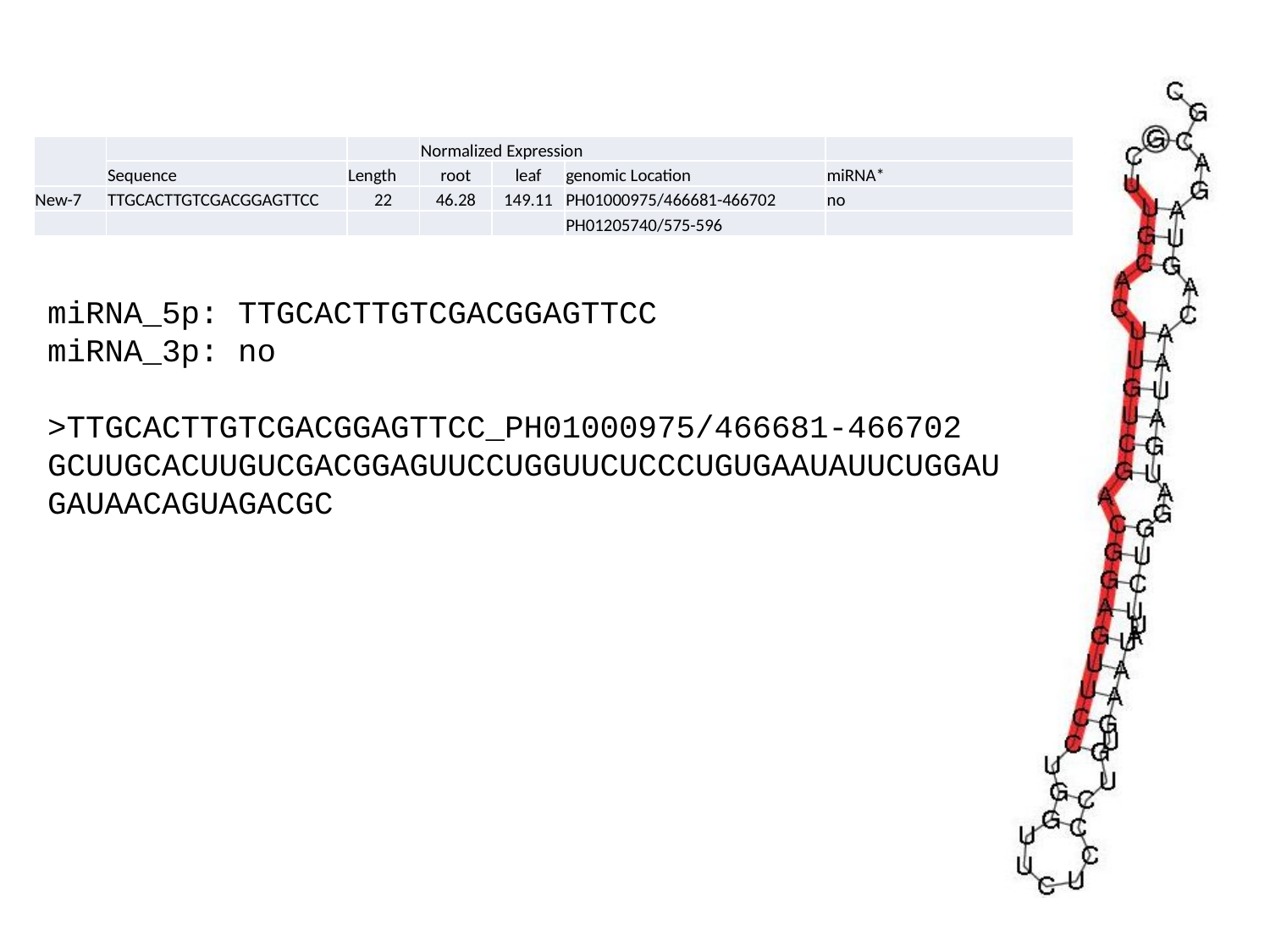

| | | | Normalized Expression | | | |
| --- | --- | --- | --- | --- | --- | --- |
| | Sequence | Length | root | leaf | genomic Location | miRNA\* |
| New-7 | TTGCACTTGTCGACGGAGTTCC | 22 | 46.28 | 149.11 | PH01000975/466681-466702 | no |
| | | | | | PH01205740/575-596 | |
miRNA_5p: TTGCACTTGTCGACGGAGTTCC
miRNA_3p: no
>TTGCACTTGTCGACGGAGTTCC_PH01000975/466681-466702
GCUUGCACUUGUCGACGGAGUUCCUGGUUCUCCCUGUGAAUAUUCUGGAUGAUAACAGUAGACGC
